# Topology and structure of an engineered human cohesin complex bound to Pds5B

**DOI:** 10.1038/ncomms12523

**Published:** 2016-08-23

**Authors:** Michael T. Hons, Pim J. Huis in ‘t Veld, Jan Kaesler, Pascaline Rombaut, Alexander Schleiffer, Franz Herzog, Holger Stark, Jan-Michael Peters

**Affiliations:** 1 Department of Structural Dynamics, Max Planck Institute for Biophysical Chemistry, Am Fassberg 11, Göttingen 37077, Germany; 2 Research Institute of Molecular Pathology (IMP), Dr Bohr-Gasse 7, Vienna 1030, Austria; 3 Department of Biochemistry, Gene Center, Ludwig-Maximilian University, Feodor-Lynen-Strasse 25, Munich 81377, Germany

## Abstract

The cohesin subunits Smc1, Smc3 and Scc1 form large tripartite rings which mediate sister chromatid cohesion and chromatin structure. These are thought to entrap DNA with the help of the associated proteins SA1/2 and Pds5A/B. Structural information is available for parts of cohesin, but analyses of entire cohesin complexes are limited by their flexibility. Here we generated a more rigid ‘bonsai' cohesin by truncating the coiled coils of Smc1 and Smc3 and used single-particle electron microscopy, chemical crosslinking-mass spectrometry and *in silico* modelling to generate three-dimensional models of cohesin bound to Pds5B. The HEAT-repeat protein Pds5B forms a curved structure around the nucleotide-binding domains of Smc1 and Smc3 and bridges the Smc3-Scc1 and SA1-Scc1 interfaces. These results indicate that Pds5B forms an integral part of the cohesin ring by contacting all other cohesin subunits, a property that may reflect the complex role of Pds5 proteins in controlling cohesin–DNA interactions.

## Abstract (web summary)

Cohesin is a ring-shaped protein complex that structures chromatin and mediates sister chromatid cohesion. Here the authors rigidify cohesin using engineered Smc1 and Smc3 and generated 3D models showing how Pds5B forms an integral part of the cohesin ring.

## Graphical abstract

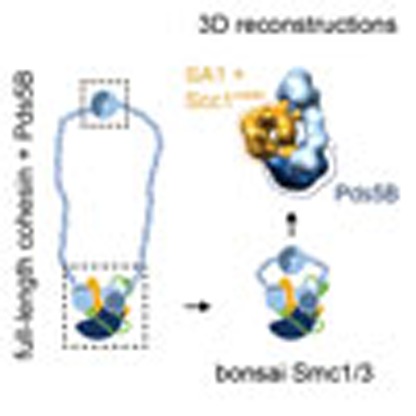

Cohesin is a protein complex that physically connects replicated DNA molecules until they are separated in anaphase^1^,^2^,^3^. This cohesion is essential for bi-orientation of sister chromatids on the spindle and is thus required for proper chromosome segregation. Cohesin also has important functions in mediating higher-order chromatin structure, gene regulation and DNA damage repair^4^ and is related to other ‘structural maintenance of chromosomes' (SMC) complexes such as condensin in both eukaryotes and bacteria^5^. Malfunctioning of cohesin can result in rare genetic diseases (cohesinopathies)^6^ and is thought to be a major cause of trisomy 21 and spontaneous human abortions^7^. Mutations in cohesin genes have also been found in human cancers^8^, with the cohesin subunit gene *STAG2* (also known as SA2) being one of only 12 human genes which are significantly mutated in >4 cancer types^9^. Understanding the structure of cohesin and how this is used to perform cohesin's various functions on DNA is therefore of great importance.

Biochemical experiments^10^,^11^ and electron microscopy (EM)^12^,^13^ revealed that cohesin complexes form ring-shaped structures. Experiments with budding yeast mini-chromosomes indicate that cohesin mediates cohesion by entrapping sister DNA strands inside this ring structure^14^,^15^. In human cells, cohesin complexes can be composed of up to seven subunits (Smc1, Smc3, Scc1, SA1 or SA2, Pds5A or Pds5B, Wapl, sororin). Only three of these, Smc1, Smc3 and Scc1 (also known as Rad21 or Mcd1), are required to generate molecular rings. These are formed at one end by hetero-dimerization of the long coiled coil subunits Smc1 and Smc3 via their hinge regions, and at the other end by the ‘closing' (greek ‘kleisin'^16^) function of Scc1, which connects the nucleotide-binding domain (NBD) of Smc1 with the NBD-proximal coiled coil region of Smc3 (refs 13, 14, 17). The latter interface between Smc3 and Scc1 is thought to function as a regulatable ‘DNA gate', which can be opened so that DNA can pass through^13^,^14^,^18^,^19^,^20^. Gate opening requires Wapl (refs 21, 22) and possibly its binding partner Pds5 (refs 23, 24), which in vertebrates exists in two isoforms, Pds5A and Pds5B (ref. 25). Pds5 proteins bind to Scc1 directly, whereas Wapl interacts with Pds5 proteins and SA1 or SA2 (refs 13, 23, 24). Because Wapl can release cohesin from DNA, cohesin has to be stabilized on DNA once it has generated cohesive structures during DNA replication, so that cohesion can be maintained throughout G2-phase and early mitosis. This is achieved by acetylation of Smc3 (refs 23, 26, 27). In vertebrates, this modification leads to recruitment of sororin^28^,^29^, which inhibits Wapl and thereby prevents cohesin release from DNA^29^. In early mitosis, sororin bound to cohesin on chromosomes arms is inactivated by phosphorylation^30^,^31^, which together with SA2 phosphorylation^32^ leads to cohesin release by Wapl and partial loss of cohesion between chromosome arms. At centromeres, sororin-bound cohesin is protected by protein phosphatase 2A bound to Sgo1 (ref. 33), and these Wapl-resistant complexes are removed from chromosomes in metaphase by the protease separase^34^,^35^. Like Wapl, separase opens the cohesin ring, but in this case by cleaving the Scc1 subunit.

Structural information has been obtained for the three ring-forming subunit interfaces of cohesin (Smc1–Smc3, Smc1–Scc1, Scc1–Smc3)^14^,^17^,^36^, for parts of Scc1, SA2 and Wapl (refs 37, 38, 39, 40) and structures have been predicted for the Huntingtin-Elongation factor 3-A subunit-TOR (HEAT) repeats of Pds5 proteins^13^. After submission of this manuscript, crystal structures of Pds5 proteins bound to fragments of Scc1 (refs 41, 42) or Wapl and sororin^43^ have also been reported. However, little is known about how Pds5 proteins interact with entire cohesin complexes. Addressing this question is important as Pds5 proteins have essential functions which are not understood at the mechanistic level. These include positive roles in establishment and/or maintenance of cohesion^25^,^44^,^45^,^46^,^47^,^48^ such as Smc3 acetylation^49^,^50^,^51^ and recruitment of sororin^29^, but also negative effects on cohesion^52^ such as release of cohesin from DNA^23^,^24^.

Likewise, little is known about cohesin's overall architecture, which so far has only been visualized by rotary shadowing EM^12^,^13^. These studies analysed cohesin complexes composed of Smc1, Smc3, Scc1 and either SA1 or SA2, isolated either from HeLa cells or *Xenopus* eggs^12^ or assembled from recombinant human subunits^13^. Although ring-shaped cohesin complexes could be seen in these experiments, they also revealed a high degree of structural flexibility, observed in the 50-nm-long coiled coil regions of Smc1 and Smc3. Because such flexibility is incompatible with single-particle EM techniques based on image averaging, we generated structurally more rigid ‘bonsai' cohesin by truncating the coiled coils of Smc1 and Smc3, following an approach previously used for the kinetochore Ndc80 complex^53^. We obtained low-resolution three-dimensional (3D) structures of bonsai trimers (Smc1^B^, Smc3^B^, Scc1) bound to either SA1, or Pds5B or both, and used these together with crosslinking-mass spectrometry to analyse how SA1 and Pds5B interact with cohesin. The results of these experiments imply that Pds5B is not simply associated with the cohesin ring only via binding to Scc1, but forms an integral part of the ring by forming contacts also with Smc1, Smc3 and SA1. This structural information may help to explain the numerous functions that have been attributed to Pds5 proteins.

## Results

### Analysis of full-length human cohesin by EM

**Figure 1:**
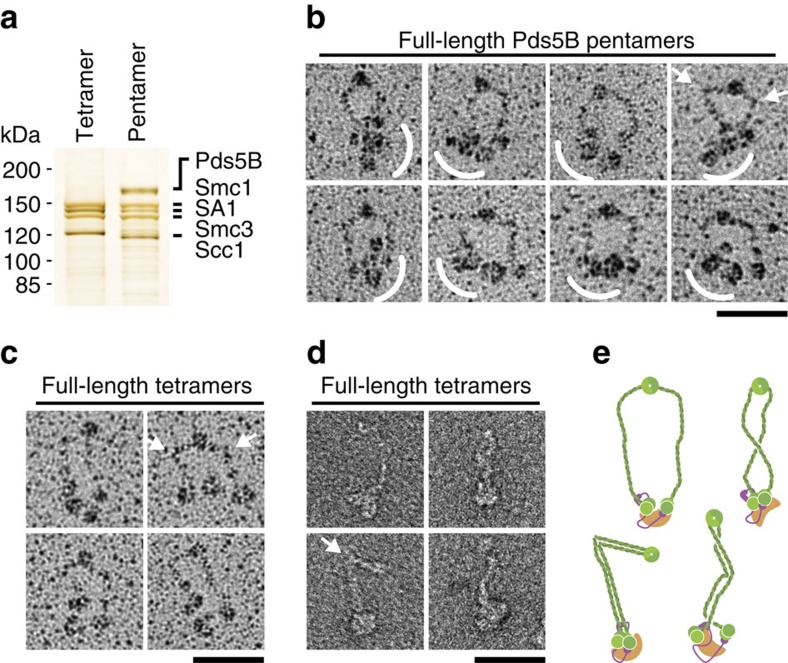
EM analysis of full-length cohesin complexes. (**a**) Pds5B was bound to full-length cohesin tetramers and analysed by silver staining after SDS–PAGE and by low-angle metal shadowing after glycerol spraying. Scc1 in the tetrameric complex contains three copies of a protease recognition site, resulting in an added molecular weight of 2.8 kDa. (**b**) The presence of the 165 kDa Pds5B resulted in an additional density near the NBDs of Smc1 and Smc3 (curved white line). The arrows indicate kinks in the coiled coils of Smc1 and Smc3. An image gallery (*n*=196) is shown in Supplementary Fig. 1. (**c**) Four representative micrographs of metal shadowed full-length cohesin (Scc1^GFP–TEV^) were selected from Supplementary Fig. 1D of ref. 13 (**d**,**e**) Micrographs of full-length cohesin complexes stained with uranyl formate and schematic representations of their putative ring-like and rod-like conformations. See Supplementary Fig. 2 for further information. Scale bars, 50 nm. Uncropped gels are shown in Supplementary Fig. 15.

We observed previously that human recombinant cohesin complexes containing full-length Smc1, Smc3, Scc1 and SA1 (500 kDa) can be visualized by rotary shadowing EM as ring-shaped complexes of variable shape^13^, closely resembling cohesin purified from human or amphibian cells and analysed by the same technique^12^. To obtain insight into how Pds5 proteins interact with cohesin, we bound recombinant purified human Pds5B to cohesin tetramers prepared as above and analysed these complexes by SDS–PAGE and silver staining (Fig. 1a) and rotary shadowing EM (Fig. 1b and Supplementary Fig. 1). Compared with cohesin tetramers (Fig. 1c), an additional density was observed in Pds5B-containing complexes in close vicinity to the NBDs of Smc1 and Smc3. Otherwise these complexes showed the ring conformation characteristic for cohesin tetramers, often with kinks in the coiled coil regions of Smc1 and Smc3. These observations are consistent with Pds5B binding to Scc1, as previously observed in biochemical experiments^13^,^23^,^24^, and indicate that Pds5B does not cause conformational changes in cohesin detectable by rotary shadowing EM.

Because rotary shadowed specimens cannot be used for determining 3D structures, we tested if cohesin complexes could be analysed by negative staining EM. For this purpose, we purified tetrameric complexes composed of Smc1, Smc3, Scc1 and SA1 by glycerol density gradient centrifugation and stained them with uranyl formate (Fig. 1d and Supplementary Fig. 2). Also under these conditions structures were seen that are reminiscent of cohesin's ‘open' ring conformation, although the thin coiled coils of Smc1 and Smc3 (diameter 1 nm) are more difficult to visualize by negative staining than by rotary shadowing. Unexpectedly, however, negatively stained cohesin complexes often had a different, rod-shaped conformation in which the coiled coils of Smc1 and Smc3 appeared connected (Fig. 1d,e and Supplementary Fig. 2), resembling a conformation also observed for bacterial SMC complexes and human condensin^12^,^54^. In some cases, these rods were not straight but kinked, possibly reflecting the kinks that have also been observed by rotary shadowing in the ring-like conformation of cohesin^12^,^13^. Together with the globular domain visible at one end of the rods, presumably representing the NBDs, Scc1 and SA1, these kinked rod-shaped complexes resemble the symbols typically used for musical notes. More work will be required to understand why rotary shadowed and negatively stained cohesin complexes appear in different conformations and which of these reflects the structure of cohesin under physiological conditions, but we note that the existence of rod-shaped cohesin conformations may be biologically relevant as crosslinking-mass spectrometry performed with cohesin in solution revealed numerous contacts between the coiled coils of Smc1 and Smc3 (refs 13, 55), and because crystallographic analysis of bacterial condensin has unequivocally shown that the coiled coils in this complex can exist in direct juxtaposition^56^.

### Generation and characterization of ‘bonsai' cohesin

**Figure 2:**
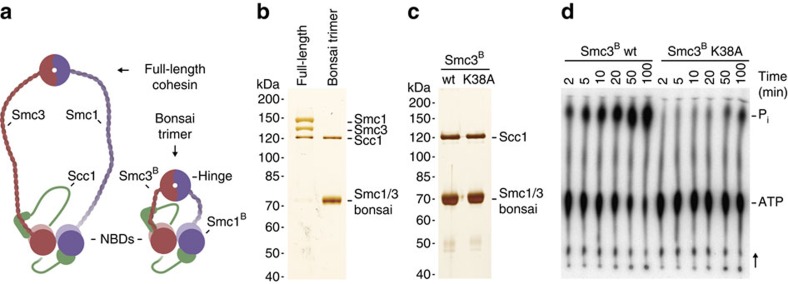
Design and characterization of bonsai cohesin. (**a**) Schematic representation of full-length and bonsai cohesin. (**b**,**c**) Full-length and bonsai trimers (Smc3^B^ or Smc3^B-K38A^) were purified and analysed by silver staining after SDS–PAGE. (**d**) Reaction mixtures including radiolabelled γ-[^32^P]-ATP and Smc3^B^ or Smc3^B-K38A^ bonsai cohesin were incubated for the times indicated. Thin layer chromatography was used to separate γ-[^32^P]-ATP and the released [^32^P]-P_*i*_. The single point mutation largely abolished bonsai cohesin's ATPase activity.

The visualization of cohesin by negative staining revealed a high degree of flexibility of full-length cohesin, as seen by rotary shadowing, precluding structure determination by single-particle analyses. We therefore engineered ‘bonsai' versions of Smc1 and Smc3 that largely lack their coiled coil regions (Fig. 2a and Supplementary Fig. 3), inspired by the previous finding that truncating the coiled coils of the Ndc80 complex enabled its structure determination^53^. Bonsai SMCs (Smc1^B^ and Smc3^B^; approximately half the size of their full-length equivalents) were co-expressed with Scc1 in Baculovirus-infected insect cells and purified as stoichiometric trimers (Fig. 2b). Importantly, these assemblies still contain the hinge domains with which Smc1 and Smc3 interact with low-nanomolar affinity^36^. Bonsai trimers containing wild-type NBDs, but not those containing a mutation in the ATP-binding region of Smc3^B^ (K38A), were able to hydrolyse ATP, indicating that the coiled coil truncations do not prevent NBD interactions in bonsai cohesin (Fig. 2c,d). The ATPase rate of bonsai cohesin is comparable to that of full-length cohesin (data not shown). Differential scanning fluorimetry showed that the presence of the slowly hydrolyzable ATP-analogue ATPyS, as well as a near physiological pH improved the stability of bonsai cohesin (Supplementary Fig. 4). Samples for single-particle EM were prepared accordingly.

### A 3D model of tetrameric bonsai cohesin containing SA1

**Figure 3:**
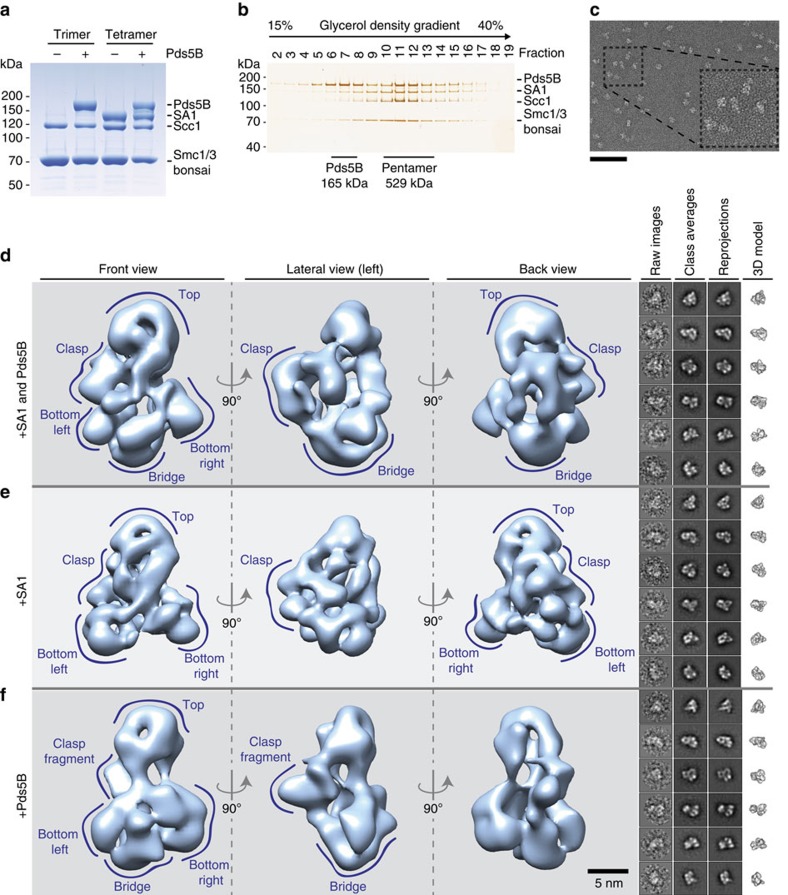
Structure of three different bonsai cohesin complexes. (**a**) The binding of Pds5B to trimeric and tetrameric bonsai cohesin yielded four distinct complexes. (**b**) Pds5B-bound bonsai cohesin was sedimented on a glycerol gradient. The presence of Pds5B caused a shift in migration. Free Pds5B sediments in fractions 6–8. See Supplementary Figs 5 and 6 for more information. (**c**) Representative electron micrograph of GraFix prepared Pds5B-bound bonsai cohesin. Scale bar, 100 nm. (**d**–**f**) Three distinct perspectives were selected to illustrate the architecture of the calculated 3D reconstructions. Raw images and class averages were compared with reprojections of the calculated models in six representative orientations. Similar regions are indicated in the respective 3D reconstructions, as top, bottom left, bottom right, clasp and bridge. See the main text and Supplementary Figs 7–10 for more information. Scale bar, 5 nm.

To obtain insight into cohesin's structure, we co-expressed the bonsai trimers characterized above with SA1 and purified tetrameric complexes. Recombinant Pds5B could be bound to these complexes, resulting in four different complexes for further structural analysis (Fig. 3a). Pds5B did not bind Smc1^B^–Smc3^B^ dimers, indicating that Scc1 is required for the interaction of Pds5B with bonsai cohesin, as observed for full-length complexes^13^ (Supplementary Fig. 5). Complexes were purified by glycerol density gradient centrifugation, either in the absence of glutaraldehyde to enable sample analysis and quality control by SDS–PAGE and silver staining (Fig. 3b and Supplementary Fig. 6) or following the GraFix method^57^ (that is, in the presence of low glutaraldehyde concentrations to preserve structural integrity of cohesin) for negative staining EM (Fig. 3c and Supplementary Fig. 7). The presence of Pds5B increased the sedimentation of these complexes, implying that many of them interacted with Pds5B stably enough to allow their purification (Supplementary Fig. 6).

We used single-particle analysis to calculate models of tetrameric bonsai cohesin (Smc1^B^, Smc3^B^, Scc1 and SA1; 367 kDa) and pentameric bonsai cohesin (Smc1^B^, Smc3^B^, Scc1, SA1 and Pds5B; 533 kDa) (Supplementary Fig. 7 and Fig. 3d, respectively). Calculations were performed independently to prevent any model bias. This resulted in two models with comparable features: the alleged front side contains three fragments (‘top', ‘bottom left', ‘bottom right') with comparable dimensions that are separated by regions with lower density. The fourth fragment (termed ‘clasp') is considerably larger and is positioned at the back (Fig. 3d and Supplementary Fig. 7). An additional density (termed ‘bridge') in the pentamer was observed near the NBDs of Smc1^B^ and Smc3^B^, possibly revealing the position of Pds5B (Fig. 3d). How we interpret the structures with respect to the location of subunits and domains will be described below.

Computational sorting of the bonsai pentamer data set for the presence of this putative Pds5B density identified a fraction of images where this density was missing (Supplementary Fig. 8). Further processing of this subset (10,582 particle images) resulted in a 3D model with the characteristic top, clasp, bottom left and bottom right densities (Fig. 3e), resembling the independently calculated 3D model of tetrameric cohesin (Smc1^B^, Smc3^B^, Scc1, SA1) (Supplementary Fig. 7). The model was determined at a resolution of 28 Å (FSC 0.5 criterion) (Supplementary Fig. 9). We conclude that the pentameric bonsai cohesin sample contained a Pds5B-bound and a Pds5B-free population, presumably because Pds5B had dissociated from some cohesin complexes during sample preparation. This is consistent with the observation that Pds5B was present in two ‘peaks' in glycerol density gradient fractions, only one of which also contained cohesin (Fig. 3b), and with results from fluorescence recovery after photobleaching (FRAP) experiments which indicate that Pds5 proteins interact dynamically with cohesin *in vivo*^58^. In both 3D models (Fig. 3d,e), the globular bottom left and bottom right regions are ∼10nm apart and connected to the top region via an elongated density. The dimension and the central cavity of the top region are consistent with the crystal structure of the mouse Smc1–Smc3 hinge domain^36^ (Supplementary Fig. 10). This top region represents ∼15% of the total density of the Smc1^B^–Smc3^B^–Scc1–SA1 model (367 kDa), corresponding well to the molecular mass of the hinge domain (49 kDa). We therefore attribute the top region to the Smc1–Smc3 hinge (Supplementary Fig. 10) and the bottom densities to the NBD of Smc3 bound to the N terminus of Scc1 (Smc3^NBD^–Scc1^N^) and to the NBD of Smc1 bound to the C terminus of Scc1 (Smc1^NBD^–Scc1^C^; Fig. 3e). According to this interpretation, the elongated density that connects the hinge and the NBD domains represents the residual coiled coil regions of Smc1^B^ and Smc3^B^. For reasons that are explained below, we assume that the clasp represents SA1 bound to the central region of Scc1 (Scc1^middle^). At the present resolution, we cannot distinguish which bottom density represents Smc3^NBD^–Scc1^N^ and Smc1^NBD^–Scc1^C^. The 10-nm distance between the bottom densities implies that ATPyS did not trap bonsai cohesin in a NBD engaged, despite its stabilizing effect on the complex.

### Pds5B curves around the NBDs of Smc1 and Smc3

**Figure 4:**
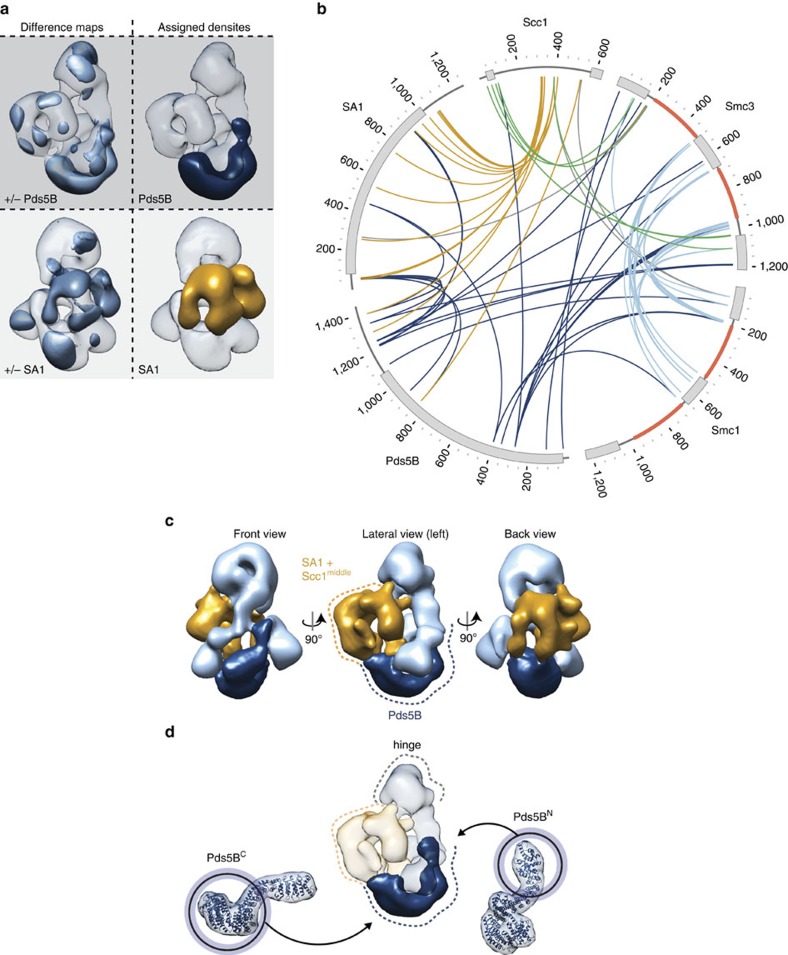
Density assignment and topology of engineered cohesin complexes. (**a**) Difference maps and assigned densities for Pds5B (left lateral view) and SA1 (back view). See also Supplementary Fig. 11. (**b**) A Circos plot of the 102 intermolecular crosslinks that were identified in bonsai cohesin bound to Pds5B. The truncated regions in Smc1^B^ and Smc3^B^ are shown in orange. Grey boxes illustrate NBDs and hinge domains of Smc1 and Smc3, alpha-helical regions in Scc1 and the alpha-helical repeats of SA1 and Pds5B. Crosslinks are coloured light blue (Smc1^B^–Smc3^B^), green (Scc1^N^–Smc3^B^), gold (Scc1middle-SA1/Pds5B), dark blue (other Pds5B) or grey (others). (**c**) The model of Pds5-bound bonsai cohesin shown in three different orientations. The curved density near the bottom fragments (dark blue) corresponds to Pds5B and the clasp-like density in the back (gold) to SA1 and Scc1^middle^. (**d**) Comparison of a downfiltered structure of human Pds5B structure (PDB: 5HDT)^43^ to Pds5B-bound tetrameric bonsai cohesin. Pds5B bound to cohesin appears to be highly curved.

To analyse the structure of bonsai cohesin bound to Pds5B, 10,416 images of pentamers were used to generate a 3D model with a determined resolution of 35 Å (FSC 0.5) (Fig. 3d and Supplementary Fig. 9). Compared with the model of tetrameric bonsai cohesin without Pds5B (Fig. 3e and Supplementary Fig. 7), a prominent additional density (bridge) is evident below the bottom left and bottom right densities (Fig. 3d, Fig. 4a,c and Supplementary Fig. 8, Supplementary Fig. 10). This density reappeared when reconstructions were calculated using a reference model from which this density had been manually removed (Supplementary Fig. 8), confirming that it represents a density that is specifically present in cohesin pentamers. We therefore assume that this density represents Pds5B. Consistent with recently determined structures of Pds5 from three different organisms^41^,^42^,^43^, the Pds5B density has an elongated shape with an overall length of 17 nm.

### SA1 and parts of Scc1 form cohesin's backside clasp

If the density assignment of Pds5B, the hinge domain, and the NBDs is correct, the clasp-like density that resides behind the residual coiled coils of Smc1 and Smc3 corresponds to SA1 (146 kDa) and Scc1^middle^. To test this, we analysed the structure of bonsai cohesin complexes lacking SA1. We were unable to obtain 3D models of bonsai trimers (Smc1^B^–Smc3^B^–Scc1; 222 kDa), possibly because of the relatively small size and pseudo-symmetrical structure of this complex. However, we were able to analyse images of these trimers when they were bound to Pds5B (387 kDa). Processing was again performed *de novo* to avoid reference bias of previous models. A 35 Å (FSC 0.5) model was calculated using images of 4,027 particles (Fig. 3f and Supplementary Fig. 9). Consistent with the assignment described above, these complexes also contained a top region (corresponding to the Smc1–Smc3 hinge), the bottom densities (representing Smc3^NBD^–Scc1^N^ and Smc1^NBD^–Scc1^C^) and the curved bridge density assigned to Pds5B. In contrast, less density was observed in the region which in SA1-containing complexes is occupied by the clasp-like structure, although a smaller density was still observed there (Figs 3f and 4a,c and Supplementary Figs 10 and 11). The majority of the clasp-like density is thus formed by SA1, whereas the central region of Scc1, and possibly also fragments of the residual coiled coils and Pds5B, contribute to the clasp fragment that remains present in the absence of SA1. This interpretation attributes a central position to Scc1^middle^, consistent with Scc1 bridging the NBDs of Smc1 and Smc3 and recruiting both SA1 and Pds5B to cohesin. Since parts of SA1 and Pds5 are in direct proximity and both proteins bind Scc1, it is likely that their exact positions and conformations are interdependent. This could account for differences between the various complexes, such as change in the position of Pds5B or a twist of the top region that is observed in the absence of SA1 (Supplementary Fig. 10). However, the biological relevance of such effects remains unclear, as there is no evidence for the existence of cohesin complexes lacking SA1 (or SA2) *in vivo*.

### Proximity map and topology of bonsai cohesin bound to Pds5B

To further validate and interpret our 3D models, we used chemical crosslinking followed by mass spectrometry and generated a map of distance constraints with 102 inter- and 244 intramolecular crosslinks (Fig. 4b and Supplementary Figs 13 and 14; and Supplementary Tables 1 and 2). Increased protein amounts and optimized crosslinking conditions improved the coverage of the proximity map compared with a previously determined map of full-length cohesin in which 53 intermolecular crosslinks were identified, only 19 of these in regions also present in the bonsai–Pds5 complex^13^.

Crosslinks between the hinge domains and the residual coiled coil regions of Smc1^B^ (197, 200, 500, 540 and 561) and Smc3^B^ (492, 493, 503, 673, 680, 977, 978, 984, 997 and 999) accounted for 39 of the 102 intermolecular crosslinks (light blue, Fig. 4b and Supplementary Table 1). The proximity of these residues (all numbered as in full-length human cohesin) is further supported by an extensive network of intramolecular crosslinks (Supplementary Fig. 13 and Supplementary Table 2) and consistent with the attribution of the residual coiled coil regions to the solvent-accessible X-shaped density of our model. Pds5B^K282^ crosslinks extensively with multiple of these residues, indicating that it points towards the residual coiled coils of bonsai cohesin.

Crosslinks between lysines 185, 188, 1,034 and 1,038 of Smc3^B^ and lysines 50, 72 and 86 of Scc1^N^ (green, Fig. 4b) are in agreement with a crystal structure of the yeast Smc3–Scc1^N^ interface^14^ and reveal that the pairing of Smc3 with Scc1^N^ is retained in the residual coiled coil fragment of Smc3^B^. Crosslinks between Scc1^317–406^ and SA1 (gold, Fig. 4b) indicate extensive contacts between Scc1^middle^ and SA1. Spatial constraints imposed by these crosslinks are largely consistent with the structure of human SA2 in complex with a Scc1^281–420^ fragment^38^, suggesting that SA1 and SA2 bind Scc1 in a comparable manner.

Interestingly, residues of SA1 that contact Scc1^317–406^ are far apart in sequence. Together with a network of intramolecular crosslinks, this suggests that the SA1 regions around residues 48–57, 261–273, 453, 549–558, 759, 910–916, 969–979, 1,019–1,028, 1,071–1,087 and 1,168–1,177 are all in close proximity in bonsai cohesin. The distance between these regions in the structure of the equivalent SA2-Scc1^middle^(ref. 38) exceeds the spatial constraints of our crosslinker by almost 100 Å. However, the putative proximity of these SA1 regions when bound to cohesin is consistent with the clasp-like conformation of SA1 in our model and with their positions near the flexible regions that connect HEAT repeats in SA2 (ref. 38), indicating that these crosslinks may also reflect physiologically relevant interactions.

The majority of inter- and intramolecular crosslinks that involve Pds5B were observed in its C-terminal part. Residues in this region of Pds5B (845, 925, 1,213, 1,219, 1,244, 1,344 and 1,397) are proximal to Scc1 (335, 387, 527) and SA1 (48, 49, 52, 57, 453, 626, 910 and 1,086), but also to residues in the NBD of Smc3^B^ (157, 1,190 and 1,194) (dark blue, Fig. 4b). This suggests that the charged C-terminal tail of Pds5B, which is predicted to be unstructured and was previously shown to be dispensable for binding to cohesin^13^, is flexible and resides close to both the SA1–Scc1^middle^ region and to the NBD of Smc3^B^.

While Pds5B^K282^ crosslinks with the residual coiled coils, more N-terminal residues of Pds5B (25, 36, 74, 115 and 121) seem to reside near residues 26 and 114 of Smc3^B^ and near the Smc3^B^–Scc1^N^ DNA exit gate. This proximity is further supported by mutations in this region of Pds5 in budding yeast that were identified to functionally cooperate with the acetylation of Smc3 in controlling the release of cohesin from DNA^23^. Since one end of the elongated Pds5B density contacts an NBD density in our model, we attribute this density to the N-terminal region of Pds5B, the NBD of Smc3^B^ and Scc1^N.^ and speculate that more C-terminal HEAT repeats of Pds5B curve around the NBD region towards the SA1–Scc1^middle^ interaction interface (Fig. 4d). A comparison of the recently solved structure of human Pds5B^43^, reported after we obtained and submitted the EM structure of bonsai cohesin bound to Pds5B, supports our assignment of Pds5B's N- and C-terminal regions of Pds5B. Interestingly, Pds5B seems to be curved to a higher degree when bound to cohesin than in isolation (Fig. 4d). Based on our proximity map and the assignment of the N- and C-terminal regions of Pds5, we propose that the density defined as ‘bottom right' contains Smc3^NDB^–Scc1^N^ whereas Smc1^NBD^–Scc1^C^ account for the density defined as ‘bottom left'. Although Pds5B curves around the NBDs of Smc1^B^ and Smc3^B^, the presence of Pds5B did not alter the ATPase activity of Smc1^B^–Smc3^B^–Scc1 under our assay conditions (Supplementary Fig. 12).

### *In silico* analysis of Smc1 and Smc3

**Figure 5:**
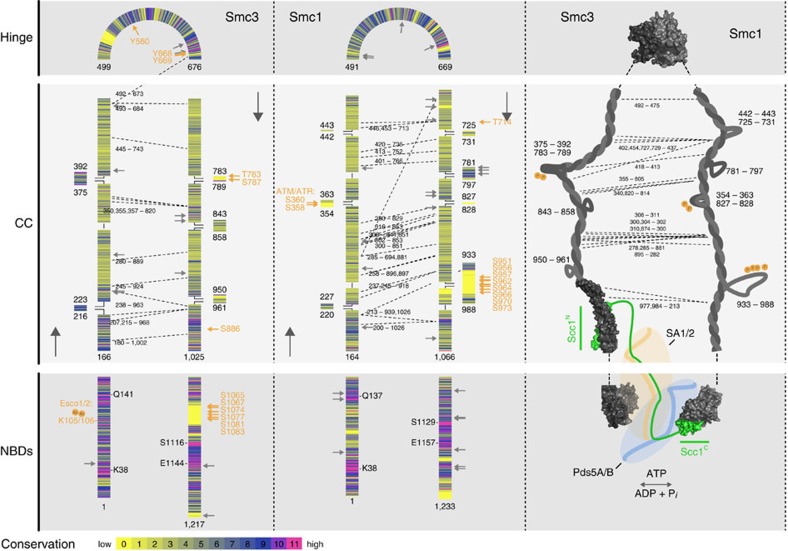
*In silico* analysis of the coiled coils of Smc1 and Smc3. SMC proteins contain nucleotide-binding domains (NBDs), anti-parallel coiled coils and a hinge domain. Multiple sequence alignments, coiled coil prediction algorithms, available crystal structures and crosslinks (dashed lines; ref. 13) were used to predict the register and architecture of cohesin's coiled coils. The borders of predicted interruptions of the coiled coils, as well as the conservation of every residue are indicated. Residues that are directly involved in the binding and hydrolysis of ATP and known phosphorylation targets^63^ (orange arrows) are indicated. Grey arrows mark residues that are mutated in CdLS patients. Predictions of the coiled coil structure and crystal structures are combined in the illustration of cohesin in the right panel. The intermolecular crosslinks indicate points of proximity between the coiled coils of Smc1 and Smc3.

Because little structural information is available about the coiled coils of Smc1 and Smc3, we analysed these regions of cohesin by *in silico* modelling. Previous work revealed that the coiled coils are formed intra-molecularly by two anti-parallel strands^11^ and that their length of almost 50 nm, as well as the position of several coiled coil-interrupting regions are conserved^59^,^60^,^61^. Compared with other coiled coil-containing proteins, the sequence of cohesin is remarkably conserved among metazoans but not among all eukaryotes^62^. Here we analysed the sequences of Smc1 and Smc3 and predicted interruptions based on sequence alignments, coiled coil prediction algorithms, available high-resolution structural information^14^,^17^,^36^ and proximity information from intramolecular crosslinks^13^ (Fig. 5). As expected, residues that are absolutely conserved in metazoans, insects, plants and fungi mainly reside in the proximity of cohesin's NBDs. A number of highly conserved residues and regions are also found in the hinge domains of Smc1 and Smc3. Within the coiled coils, Smc3^166–210^ and Smc3^976–1,025^ are well-conserved, indicating the importance of the interaction between Smc3 and Scc1^N^. Micrographs of full-length cohesin showed characteristic coiled coil disruptions in the hinge-proximal halves of the coiled coils of Smc1 and Smc3 (Fig. 1b and Supplementary Fig. 1)^12^,^13^. Based on our analysis, we expect that the non-coiled coil regions Smc3^783–789^ and Smc3^375–392^ account for a kink in the Smc3 coiled coil region. Our analysis also highlights the conservation of the non-coiled coil region Smc1^781–797^ and of residues that flank the non-coiled coil region Smc1^933–988^. Interestingly, Smc1^933–988^, as well as Smc3^1,065–1,083^, a remarkably non-conserved region within the NBD of Smc3, have previously been identified in proteomic experiments to contain numerous phosphorylation sites^63^, implying that some functions of cohesin might be regulated via post-translational modifications in these regions. Nonsense mutations and small in-frame deletions of 40 residues in Smc1 and Smc3 that have been identified in Cornelia de Lange Syndrome patients showed no obvious bias to conserved residues or regions (grey arrows, Fig. 5).

## Discussion

It is now clear that structural and mechanistic insight into how cohesin interacts with DNA will be essential for understanding the 3D organization, cohesion and repair mechanisms of eukaryotic genomes and their correct spatiotemporal expression patterns. Understanding cohesin functions will likewise be of great importance for revealing the molecular cause of several human diseases and syndromes, some as widespread as trisomy 21 and spontaneous abortions^7^. Great progress has been made in obtaining structural insight into cohesin subunit interactions and domains^14^,^17^,^36^,^37^,^38^,^39^,^40^,^41^,^42^,^43^, but the analysis of entire cohesin complexes has been limited by their inherent flexibility. To overcome this limitation, we have generated and structurally analysed engineered human cohesin. Our ability to obtain 3D models of these ‘bonsai' complexes indicates that they are structurally more rigid than full-length cohesin. Further optimization of their design might pave the way for high-resolution single-particle EM, and similar approaches could be used for other SMC complexes.

Interestingly, our 3D models revealed that the Scc1-binding subunit SA1, which in rotary shadowed specimens can be seen attached to the ring and lying either inside or outside of it^13^, is located ‘behind' Smc1^B^ and Smc3^B^ in the 3D model of bonsai cohesin, in close vicinity to their hinge and NBDs. The vicinity of SA1 to the NBDs may well be of functional relevance as previous observations have implicated orthologues and paralogues of SA1 in release of cohesin from DNA, which is thought to occur via dissociation of Scc1 from Smc3 (refs 13, 14, 18, 19, 20). In budding yeast, the SA1 orthologue Scc3 is required for release of cohesin from DNA^23^, and in human cells mitotic phosphorylation of the SA1 paralogue SA2 is necessary for Wapl-mediated release of cohesin from chromosome arms^29^,^32^. The relevance of SA1's proximity to the hinge is more difficult to interpret as it could have simply been induced by truncation of the coiled coils in bonsai cohesin. However, fluorescence resonance energy transfer (FRET) assays have detected interactions between the hinge and NBD-proximal subunits (Scc1 and Pds5)^64^, and by crosslinking-mass spectrometry we could also detect interactions of SA1 with hinge-proximal coiled coil regions in full-length cohesin^13^. SA1–hinge interactions may therefore also occur in wild-type cohesin and could regulate its functions.

The position in which SA1 and its binding partner Scc1 are typically assumed to be located, that is, directly between or next to the NBDs (depending on whether these are ATP bound and thus associated with each other or not), is occupied in our 3D model of bonsai cohesin by Pds5B. Consistent with the existence of numerous HEAT repeats in Pds5B, the density that represents Pds5B has an elongated and curved appearance. Interestingly, the location of this density is not only consistent with it binding to Scc1 (ref. 24), but the Pds5B density also directly contacts SA1 and Scc1^middle^, as well as the NBDs of Smc1^B^ and Smc3^B^. These contacts may be relevant for the observed positive and negative roles of Pds5 proteins in cohesion and raise the interesting possibility that these subunits could have direct effects on cohesin's ATP-binding and hydrolysing activities, which has been implicated in ring opening and cohesin loading. Our observation that Pds5B alone does not detectably alter the ATPase activity of cohesin does not exclude this possibility as the presence of other molecules (Wapl, DNA, the cohesin-loading complex) or post-translational modifications may be required to reveal such an effect.

Direct physical interactions between Pds5B and Scc1, SA1 and Smc1^B^ and Smc3^B^ were also detected by crosslinking-mass spectrometry. This proximity map implies that the unstructured C-terminal tail of Pds5B contacts both SA1 and Scc1 in the region where SA1 clasps around Scc1^middle^, despite the fact that this part of Pds5B is not essential for cohesin binding^13^. In budding yeast, point mutations in the N-terminal region of Pds5 prevent release of cohesin from DNA^23^. Interestingly, the corresponding N-terminal part of Pds5B appears to be closer to the Smc3–Scc1^N^ interface, raising the possibility that this part of Pds5 proteins regulates cohesin–DNA interactions either by influencing opening and closure of the DNA gate between Smc3 and Scc1, or—as discussed above—by modulating cohesin's ATPase activity. In support of the former possibility, it has been reported recently that Pds5B can inhibit closure of cohesin's exit gate *in vitro*^43^. The proximity between Pds5B and the NBD of Smc3 could also explain why Pds5 proteins are required for Smc3 acetylation^49^,^50^,^51^. In any of these cases, our observation that Pds5B occupies a similar bridge (‘kleisin')-like position between the NBDs of Smc1 and Smc3 as Scc1 implies that Pds5 proteins may control cohesin–DNA interactions by directly regulating cohesin ring opening.

Before engineering bonsai cohesin complexes, we also explored if full-length cohesin could be used for 3D structure determination by negative staining EM. Unexpectedly, under these conditions cohesin was often observed in a closed rod-shaped conformation in which the coiled coils of Smc1 and Smc3 were juxtaposed, as opposed to their clear separation in the open ring conformation that is typically seen by rotary shadowing^12^,^13^. We suspect that this rod conformation is not simply a sample preparation or staining artefact, as our previous crosslinking-mass spectrometry experiments of full-length cohesin in solution had also detected numerous close contacts between the coiled coils, and because similar rod-shaped conformations have previously been observed for eukaryotic and bacterial condensin complexes^12^,^54^,^56^. Interestingly, for the Smc–ScpAB complex in *Bacillus subtilis* it has recently been observed that ATP-dependent binding of DNA to the hinge can change the closed rod conformation to an open ring conformation^56^, and this change has been proposed to be required for topological entrapment of DNA inside all SMC complexes^56^,^65^. Our observation that human cohesin can exist in a closed rod conformation supports this hypothesis.

## Methods

### Design of bonsai cohesin

PCR and classical restriction ligation were used to replace the fragments 201–498 and 676–977 of Smc1 and 205–491 and 686–956 of Smc3 with linker sequences PG, SR, GT and AR, respectively (representing 5′-CCCGGGTCTAGAGGTACC-3′ and 5′-GCGCGC-3′ recognition sequences of the used restriction enzymes XmaI, XbaI, KpnI and BssHII) to generate Smc1^B^ and Smc3^B^. These fragments were combined with Scc1 and SA1 onto the multibac vector pFL using the multiplication module^13^,^66^ to generate constructs encoding bonsai dimers (Smc1^B-10HIS^, Smc3^B-FLAG^), bonsai trimers (Smc1^B^, Smc3^B-FLAG^, Scc1^10HIS^), bonsai tetramers (Smc1^B^, Smc3^B-FLAG^, Scc1^10HIS^SA1) and full-length tetramers (Smc1, Smc3^FLAG^, Scc1^10HIS^, SA1). The K38A mutation in Smc3^B^ was introduced by site-directed mutagenesis. A detailed description of sequence analysis procedures is included in the Supplementary Methods and Supplementary Tables 3 and 4.

### Protein purification and characterization

Baculoviruses were generated and used to express proteins in *sf9* cells^67^. All constructs were purified using tandem metal-chelate-affinity and FLAG-affinity purification as described^13^. In brief, cell lysates were generated by dounce homogenization and centrifugation in buffer A (25 mM KH_2_PO_4_ pH 7.5, 500 mM KCl, 5% v/v glycerol, 2 mM MgCl_2_) supplemented with 20 mM imidazole, 20 mM β-Mercaptoethanol, 0.05% v/v Tween-20, 0.5 mg ml^−1^ PMSF and complete protease inhibitor (Roche). Cleared lysates were incubated with Ni-NTA agarose beads (Qiagen) for 90 min. Beads were washed with buffer A+20 mM imidazole, 20 mM β-Mercaptoethanol and 0.05% v/v Tween-20 and eluted with buffer A+0,01% v/v Tween-20 and 200 mM imidazole. Eluates were incubated with anti-FLAG M2 agarose beads (Sigma) and washed with buffer B (25 mM Hepes pH 7.5, 150 mM NaCl, 5% v/v glycerol, 2 mM MgCl_2_)+0.01% v/v Tween-20. Complexes were eluted in buffer B+0.5 mg ml^−1^ FLAG peptide+183 μM ATPγS (Jena Biosciences).

Lysates of *sf9* cells expressing Pds5B^10HIS^ were generated by dounce homogenization and centrifugation in buffer C (25 mM Tris pH 7.5, 200 mM NaCl, 5% v/v glycerol) supplemented with 20 mM imidazole, 20 mM β-Mercaptoethanol, 0.05% v/v Tween-20, 0.5 mg ml^−1^ PMSF and complete protease inhibitor. Cleared lysates were incubated with Ni-NTA agarose beads for 90 min. Beads were washed with buffer C+20 mM imidazole, 20 mM β-Mercaptoethanol and 0.05% v/v Tween-20 and eluted with buffer C+0.01% v/v Tween-20 and 200 mM imidazole. Imidazole was removed by subsequent dilution in buffer C followed by concentration or by further purification by size-exclusion chromatography on a Superdex 200 10/300 or 16/600 column (GE Healthcare). Freeze–thawed Pds5B was bound to FLAG-immobilized cohesin by incubation at a large molar excess. BSA was added at 0.5 mg ml^−1^ to promote specific binding and monitor sufficient washing between Pds5B binding and elution from the FLAG beads. The Pds5B sample that was used to compare binding to bonsai dimers, trimers and tetramers (Supplementary Fig. 5) contained a very substoichiomteric amount of Wapl.

Purified protein complexes were analysed by SDS–PAGE on 7.5% or 4–12% polyacrylamide Tris-Glycine gels followed silver staining or coomassie staining. Antibodies against Smc1 (Bethyl laboratories), Smc3 (ref. 13) and Pds5B^25^ were used for analysis by immunoblotting. Detailed descriptions of ATPase assays, differential scanning fluorimetry and crosslinking followed by mass spectrometry are included in the Supplementary Methods.

### Low-angle metal coating and EM

Glycerol spraying, low-angle metal coating and EM of full-length cohesin complexes was performed as described^13^,^68^. In brief, FLAG-peptide eluates were diluted twofold with spraying buffer (200 mM ammonium acetate and 55% glycerol), sprayed onto a freshly cleaved mica and mounted in a MED020 high-vacuum evaporator (Baltec). After vacuum drying, a Platinum layer of 1-nm and a 7-nm Carbon support layer were subsequently evaporated onto the rotating specimen at respective angles of 5–6° and 45–60°. Pt/C replicas were released from the mica on water, captured by freshly glow-discharged 400-mesh Pd/Cu grids (Plano), plasma cleaned and visualized by transmission EM using a LaB_6_ equipped Tecnai 20 (FEI) operated at an accelerating voltage of 80 kV. Micrographs were recorded at a nominal magnification of 50,000 and a resolution of 2.18 Å per pixel. Well-spread cohesin particles with distinguishable coiled coils were selected from raw micrographs and analysed by ImageJ.

### Density gradients

FLAG-peptide eluates containing cohesin (100–200 μl) were applied on continuous 15–40% (v/v) glycerol gradients (25 mM Hepes pH 7.5, 150 mM NaCl, 2 mM MgCl_2_ 183 μM ATPγS) with a buffered 10% glycerol cushion (100 μl). When indicated, mild stabilization was accomplished by a modified GraFix protocol^57^ with 0.1% (v/v) glutaraldehyde in the 40% glycerol fraction and 1 mM *N*-(*p*-Maleimidophenyl)isocyanate (PMPI) in DMSO in the 15% glycerol fraction. Centrifugation was performed in a TH660 (Sorvall) or a SW60Ti (Beckmann) rotor at 45,000 r.p.m. for 16 hours. To reduce sample heterogeneity, the centrifuge temperature was lowered from 4 °C to −10 °C briefly after the maximum speed was reached. Samples shown in Fig. 1d, Supplementary Figs 2, 6 and 7 were sedimented at 4 °C. Fractions (150 μl) were collected manually from top-to-bottom by pipetting or semi-automatically from bottom-to-top with a needle coupled to a P1 pump and a Frac-920 fraction collector (GE Heatlhcare). Aspartate pH 7.9 was added at a final concentration of 10 mM to collected GraFix fractions to quench the crosslinking.

### Negative staining and EM

Protein complexes were adsorbed to a continuous carbon film and stained with freshly prepared uranyl formate (2% w/v in double-distilled water) on Quantifoil R 3.5/1 grids. Bonsai tetramer images were recorded at a Titan Krios (FEI) operated at 80 kV equipped with a 4K4K CCD detector (Eagle detector; magnification: 37,000; twofold binning, estimated 1.2 Å per pixel). All other images were recorded with a CM200 (Philips) FEG microscope operated at 160 kV and equipped with a 4K × 4K CCD camera (Tietz Video Systems; magnification: 88,000; twofold binning; 2.51 Å per pixel). The defocus was adjusted manually between 1 and 3 μm.

### Image processing

Particles were selected semi-automatically or automatically using cowPicker (in-house developed software planned for release to the wider community in the near future; B. Busche and H.S., unpublished data) yielding different sets of images for each complex (bonsai tetramer, ∼52,000 particle images; Pds5-bound bonsai tetramer, ∼113,000 particle images; Pds5-bound bonsai trimer, ∼82,000 particle images). Initial CTF correction was performed using cowEyes (M. Lüttich and H.S., unpublished data) and non-particle images and images with unsatisfying contrast and CTF features were removed (with remaining ∼33,500 particle images for bonsai tetramer; ∼50,000 particle images for Pds5-bound bonsai tetramer and ∼34,000 particle images for Pds5-bound bonsai trimer). Images were two times downsampled, filtered, normalized and used for 2D classification. Initial models were obtained using a resampling approach of 2D class averages with a subsequent 3D MSA and classification as described^69^,^70^ or by a maximum-likelihood approach implemented in Simple Prime^71^. Initial models were refined using in-house developed cowEyes and 3D classification was performed in Relion^72^. Density validation was performed by removing the density in UCSF Chimera^73^ and supervised classification^74^. Different densities of the obtained 3D models were cross-validated by 3D classification in Relion or by projection matching with distinct input 3D models and distinct classified subsets of images (Supplementary Fig. 8). Reference-free recovery of densities was judged as sign of authenticity of these densities. Particle images were sorted for the presence (Pds5B-bound bonsai tetramer, ∼19,000 particle images; Pds5-bound bonsai trimer, ∼8,000 particle images) or absence (Pds5B-free bonsai tetramers, ∼31,000 particle images) of Pds5 and used to generate improved 3D models (bonsai tetramer 3D model, 10,582 particle images; Pds5B-bound bonsai trimer 3D model, 4,027 particle images; Pds5B-bound bonsai tetramer 3D model, 10,416 particle images). Output models of Relion refinement or 3D classification were subjected to projection matching to obtain 2D class averages for comparison to reprojections. By difference mapping, the main differences between the distinct complexes (+/−Pds5B and +/−SA1) were identified and assigned to Pds5B and SA1, respectively. To do so, 3D models were aligned, equally downfiltered, normalized and subtracted. To define the absolute handedness, a random conical tilt approach^75^ on negatively stained sample and cryo prepared sample was tested but failed possibly due to particle heterogeneity, pseudo symmetry and particle size. By comparison and fitting with the available X-ray models, we propose the handedness of the 3D models as illustrated.

### Data availability

Density maps were deposited in the EM Data Bank under the accession codes EMD-4029 (+SA1, +Pds5B), EMD-4030 (+SA1) and EMD-4031 (+Pds5B). Raw mass spectrometry data are available upon request. The software suite used for particle picking and analysis is still in the development phase and will be released in the near future. The authors declare that all other data supporting the findings of this study are available within the article and its Supplementary Information Files.

## Additional information

**How to cite this article:** Hons, M. T. *et al.* Topology and structure of an engineered human cohesin complex bound to Pds5B. *Nat. Commun.* 7:12523 doi: 10.1038/ncomms12523 (2016).

## Supplementary Material

Supplementary Information Supplementary Figures 1-15, Supplementary Tables 1-4, Supplementary Methods and Supplementary References
