# Supplementary material for: Topology and structure of an engineered human cohesin complex bound to Pds5B
Source: Nat Commun. 2016 Aug 23;7:12523. doi: 10.1038/ncomms12523 (PMC4996973; doi:10.1038/ncomms12523)
Supplement: Supplementary Information — Supplementary Figures 1-15, Supplementary Tables 1-4, Supplementary Methods and Supplementary References [file ncomms12523-s1.pdf]

a

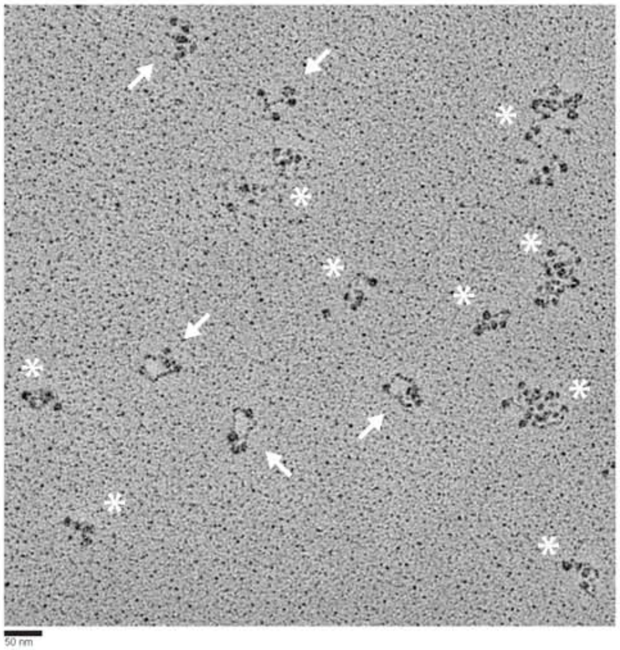

b

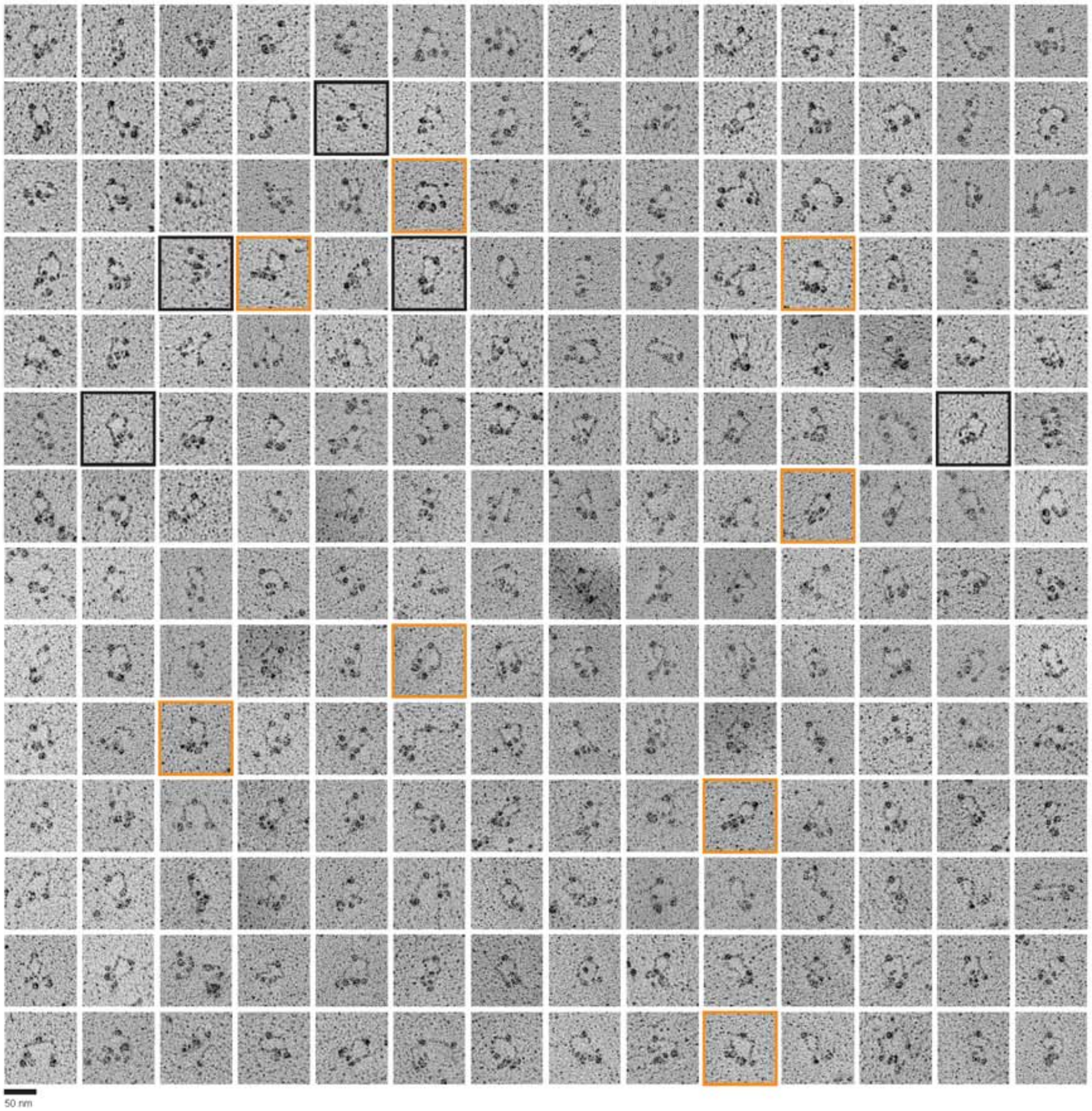

Supplementary Figure 1 Overview and image gallery of full-length Pds5 bound complexes.

(**a**) Full-length cohesin complexes bound to Pds5 were analyzed by electron microscopy after low-angle rotary metal shadowing following glycerol spraying. This representative overview contains complexes that aggregated or lacked clearly recognizable coiled coils (white asterisks) as well as well-spread complexes that were selected for further analysis (white arrows). (**b**) An image gallery showing 196 full-length Pds5 bound cohesin complexes. Selected complexes were highlighted to indicate their presence in **Supplementary Fig. 1a** (black) or in **Fig. 1b** (orange). Scale bars 50 nm.

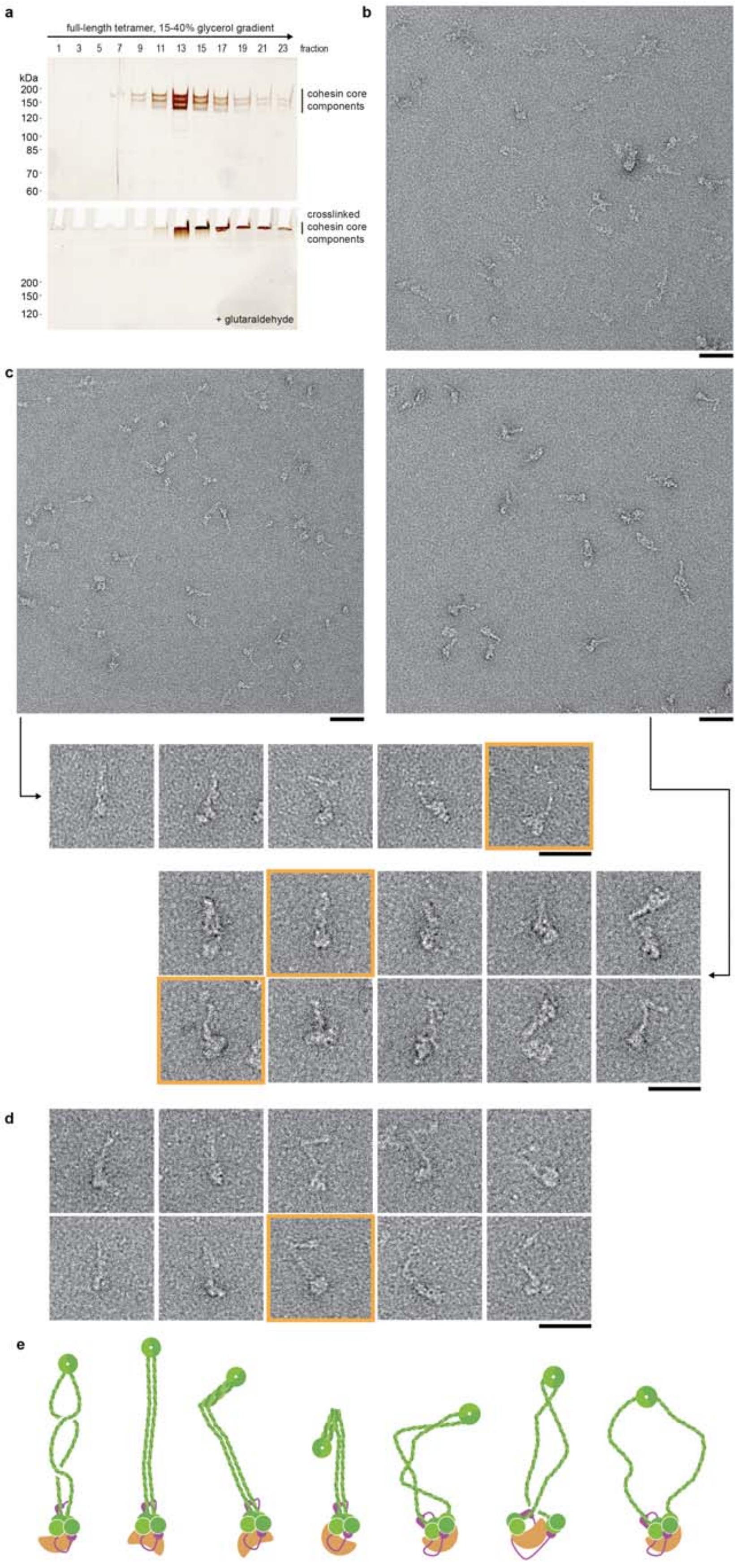
a
full-length tetramer, 15-40% glycerol gradient
1 3 5 7 9 11 13 15 17 19 21 23 fraction
kDa
200
150
120
100
85
70
60
cohesin core components
crosslinked cohesin core components
200
150
120
+ glutaraldehyde
b
c
d
e

Supplementary Figure 2 Negative staining of full-length cohesin complexes.

(a) Fractions of full-length cohesin complexes that were sedimented on a density gradient were collected and analyzed by silver staining after SDS PAGE. The GraFix preparation resulted in intermolecular crosslinks and prevented cohesin to enter the SDS-PAGE gel. (**b**) Peak fractions of sedimented full-length cohesin were inspected by negative staining EM. Rod-like particles were observed, but the amount of detail was lower than for GraFix prepared cohesin. (**c**) Two representative micrographs of GraFix prepared full-length cohesin are shown. A heterogeneous mixture of rod-like particles, particles with a notable kink, as well as collapsed or broken particles were visible. All scale bars 50 nm. (**d**) Magnification of 5 particles that were selected from the left micrograph in panel c. Micrographs boxed in orange are shown in **Fig. 1d.** (**e**) Magnification of 10 particles that were selected from the right micrograph in panel c. (**f**) 10 other selected GraFix prepared cohesin molecules. All scale bars 50 nm. (**g**) Schematic representations of possible appearances of full-length cohesin. Smc1 and Smc3 are shown in green, Scc1 in purple and SA1/2 in orange.

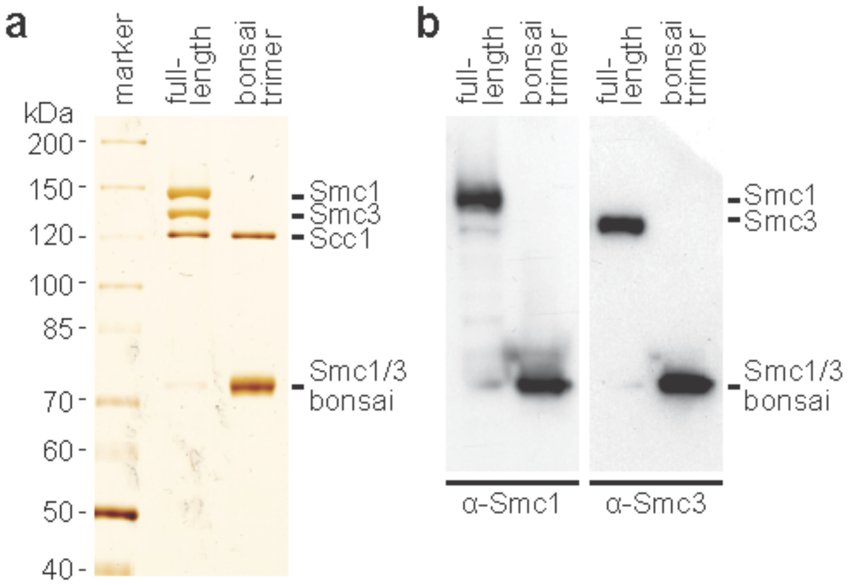

Supplementary Figure 3 Immunoblotting analysis of bonsai cohesin

(**a, b**) Bonsai cohesin trimers (Smc1^{B}-Smc3$^{B\text{-}FLAG}$-Scc1^{HIS}) were compared with full-length trimers (Smc1-Smc3^{FLAG}-Scc1^{HIS}). The migration of Smc1^{B} and Smc3$^{B\text{-}FLAG}$ is indistinguishable, but the presence of both proteins could be demonstrated using antibodies specific for Smc1 or Smc3.

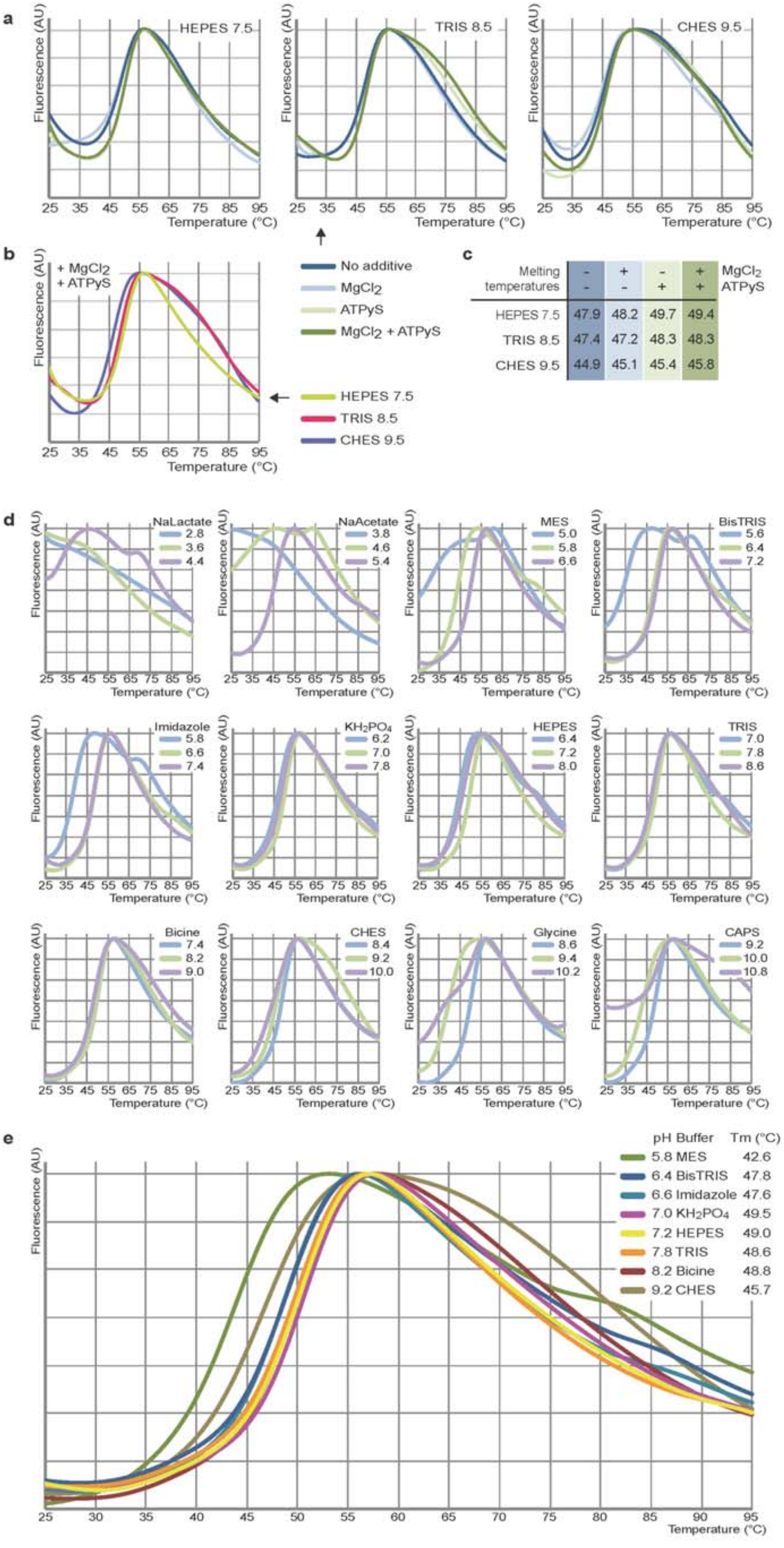
a
Fluorescence (AU)
HEPES 7.5
TRIS 8.5
CHES 9.5
25 35 45 55 65 75 85 95
Temperature (°C)
b
+ MgCl2
+ ATPyS
No additive
MgCl2
ATPyS
MgCl2 + ATPyS
HEPES 7.5
TRIS 8.5
CHES 9.5
c
Melting temperatures
MgCl2
ATPyS
HEPES 7.5 47.9 48.2 49.7 49.4
TRIS 8.5 47.4 47.2 48.3 48.3
CHES 9.5 44.9 45.1 45.4 45.8
d
NaLactate 2.8 3.6 4.4
NaAcetate 3.8 4.6 5.4
MES 5.0 5.8 6.6
BisTRIS 5.6 6.4 7.2
Imidazole 5.8 6.6 7.4
KH2PO4 6.2 7.0 7.8
HEPES 6.4 7.2 8.0
TRIS 7.0 7.8 8.6
Bicine 7.4 8.2 9.0
CHES 8.4 9.2 10.0
Glycine 8.6 9.4 10.2
CAPS 9.2 10.0 10.8
e
pH Buffer Tm (°C)
5.8 MES 42.6
6.4 BisTRIS 47.8
6.6 Imidazole 47.6
7.0 KH2PO4 49.5
7.2 HEPES 49.0
7.8 TRIS 48.6
8.2 Bicine 48.8
9.2 CHES 45.7
25 30 35 40 45 50 55 60 65 70 75 80 85 90 95
Temperature (°C)

Supplementary Figure 4 Thermal unfolding analysis of bonsai cohesin.

(**a, b**) The unfolding of bonsai cohesin upon increased temperatures was monitored by the increase in fluorescent signal. The presence of ATPyS had a stabilizing effect on bonsai cohesin, as indicated by the delayed unfolding The complex is more stable in a Hepes-buffered solution at pH 7.5 than in Tris- or CHES-buffered solutions at pH 8.5 or 9.5. (**c**) The transition point of the melting curve determined to find the melting temperature. (**d**) The unfolding of bonsai cohesin was tested in a range of different buffers. ATPyS and Mg^{2+} were present in all conditions. (**e**) A number curves from panel d were selected for a side-by-side comparison.

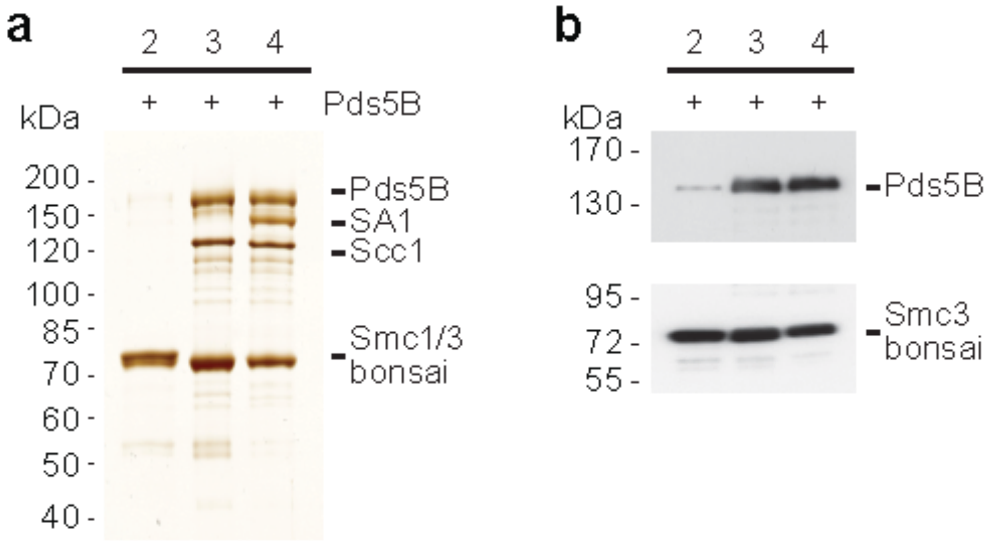

Supplementary Figure 5 Bonsai cohesin that contains Scc1 can recruit Pds5B.

(**a, b**) Bonsai cohesin dimers (2; Smc1$^{B\text{-}HIS}$-Smc3$^{B\text{-}FLAG}$), trimers (3; Smc1^{B}-Smc3$^{B\text{-}FLAG}$-Scc1^{HIS}) and tetramers (4; Smc1^{B}-Smc3$^{B\text{-}FLAG}$-Scc1-SA1^{HIS}) were immobilized on beads through the FLAG-tag on Smc3^{B} and were incubated with Pds5B. After extensive washing, material bound to the beads was analyzed by silver staining and immunoblotting after SDS-PAGE. Smc1$^{B\text{-}HIS}$ migrates slightly above Smc1^{B} and Smc3$^{B\text{-}FLAG}$.

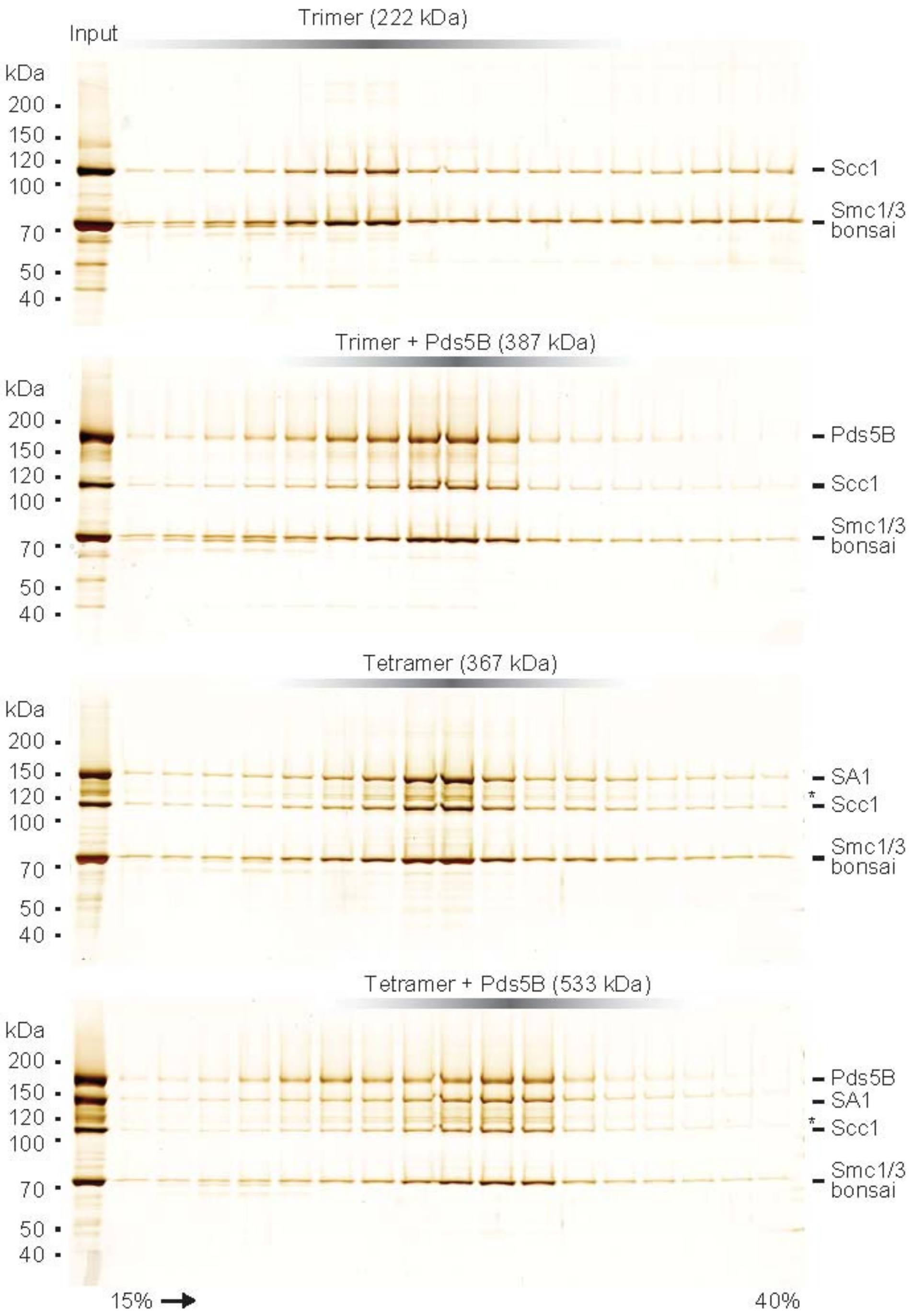

Supplementary Figure 6 Sedimentation comparison of several bonsai assemblies.

All four distinct bonsai cohesin complexes were separated by sedimentation on a continuous 15% to 40% glycerol gradient. Collected fractions were analyzed by silver staining after SDS-PAGE.

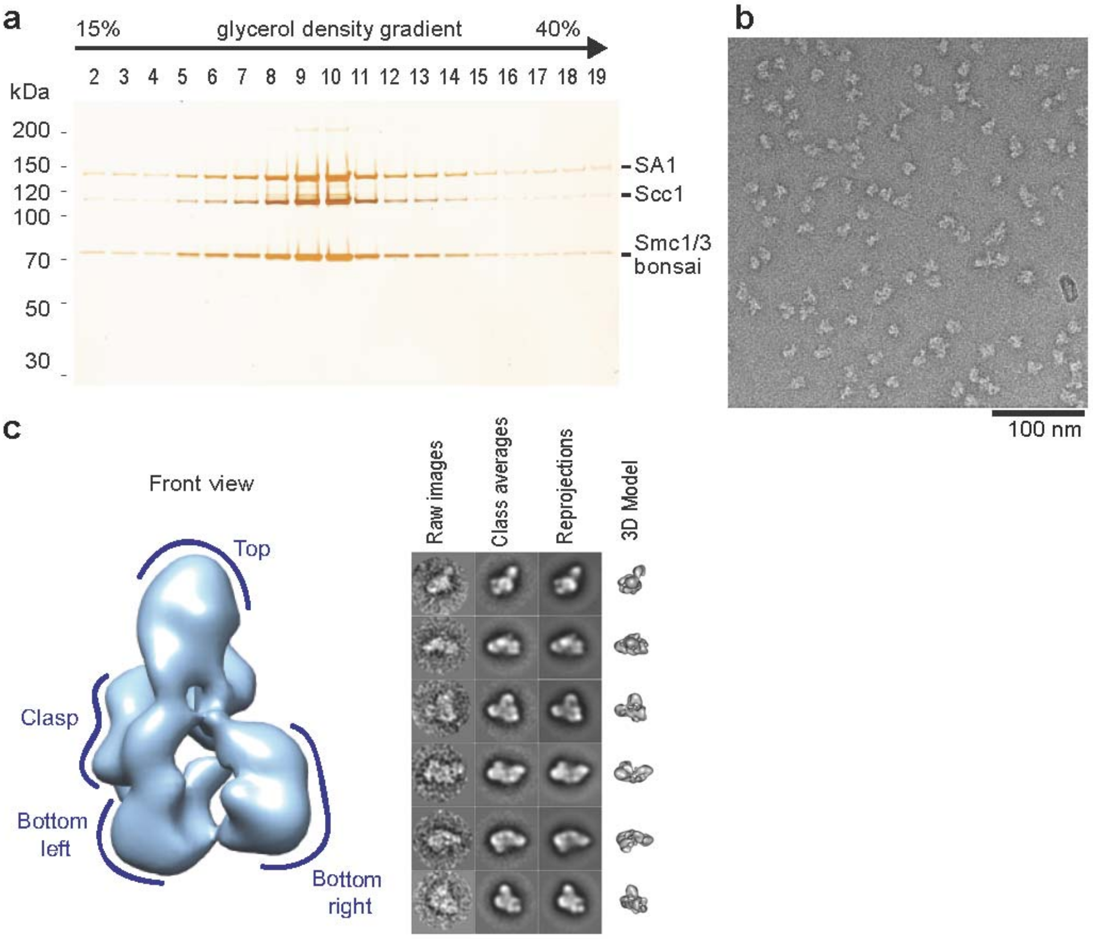

Supplementary Figure 7 Generation of the initial bonsai tetramer model.

(a) Tetrameric bonsai cohesin (Smc1^B-Smc3^B-Scc1-SA1) was sedimented on a glycerol density gradient. This gradient and the one shown in **Fig. 3b** were fractionated identically to allow a comparison of the sedimentation behavior in the presence and absence of Pds5B. The complex peaked in fractions 9 and 10, as analyzed by silver staining after SDS-PAGE. (**b)** Representative electron micrograph of negative-stained GraFix prepared tetrameric bonsai cohesin. Scale bar 100 nm. (**c, d)** Initial 3D model of bonsai cohesin tetramers and a comparison of representative reprojections to raw images and class averages.

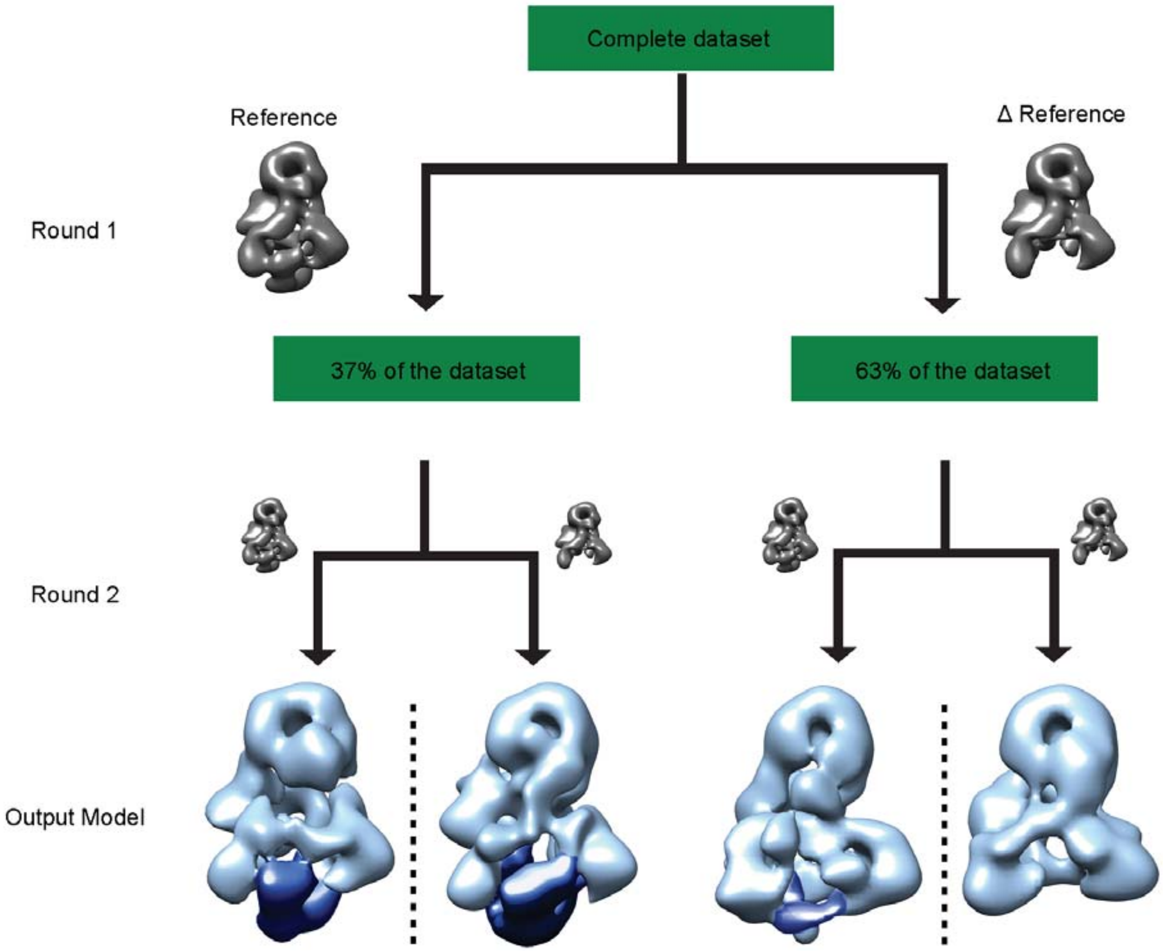

Supplementary Figure 8 Pds5B density reappears after manual modification of the reference model. Sorting the dataset for this density allowed us to improve the model, to identify a compositional heterogeneity based on Pds5B binding and to validate the authenticity of the extra density. The fraction of particles sorted for the Pds5B density could reproduce the extra density independent of the applied reference (left), whereas the second fraction allowed no reappearance of this extra density independent of the applied reference (right). This Pds5B-free fraction enabled the reconstruction of an improved model of bonsai cohesin tetramer (**Fig. 3**).

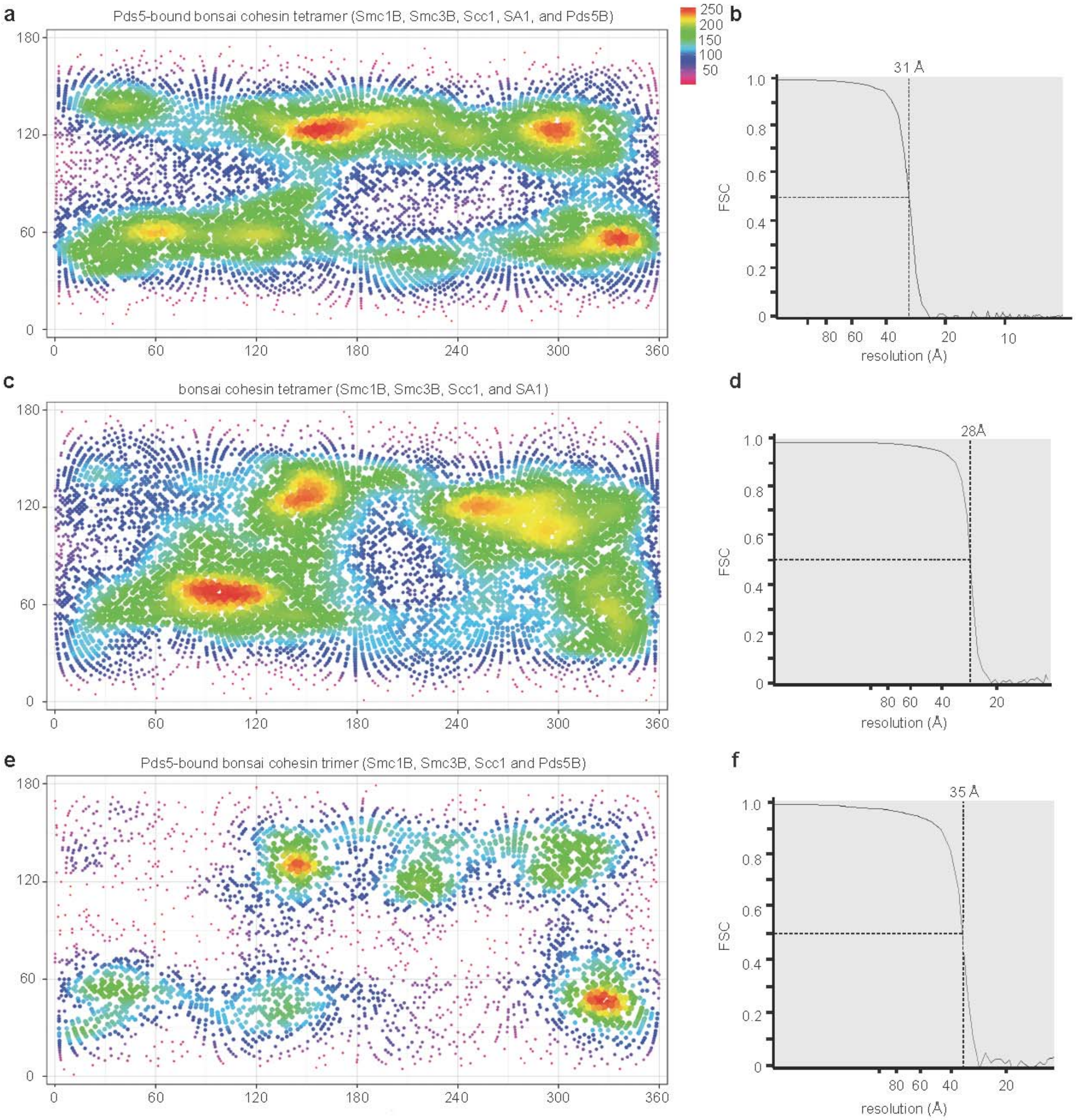

Supplementary Figure 9 Euler Plots and FSC curves of the three bonsai cohesin models.

Distribution of images according to euler angle β (y axis) and euler angle γ (x axis) of tetramer (**a**), pentamer (**c**) and Pds5B-bound trimer (**e**). The heatmap relates to the number of images assigned to each perspective. Representative perspectives are depicted in **Fig. 3d-f**. The resolution is highlighted according to the FSC 0.5 criterion for the tetramer (**b**), pentamer (**d**) and Pds5B-bound trimer (**f**).

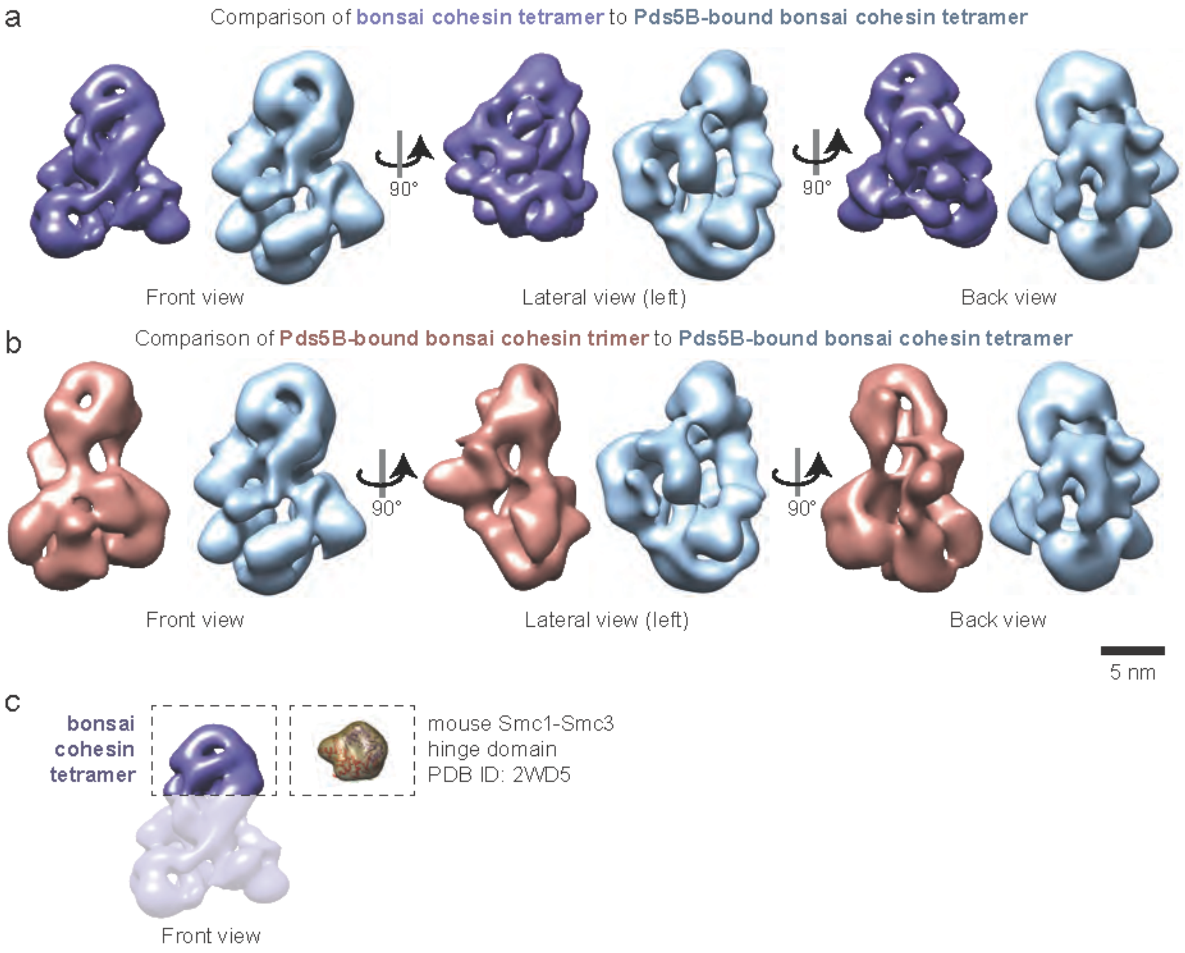

Supplementary Figure 10 Comparison of the different bonsai complexes in three orientations. (**a**) Side-by-side comparison of bonsai cohesin tetramers with (light blue) and without Pds5B (purple) in front view, left lateral view and back view. (**b**) Side-by-side comparison of bonsai cohesin with Pds5B bound in the presence (light blue) or absence of SA1 (light red) in front view, left lateral view and back view. (**c**) Comparison between bonsai cohesin tetramer and a down-filtered crystal structure of Smc1/3 hinge (PDB: 2WD5[1]).

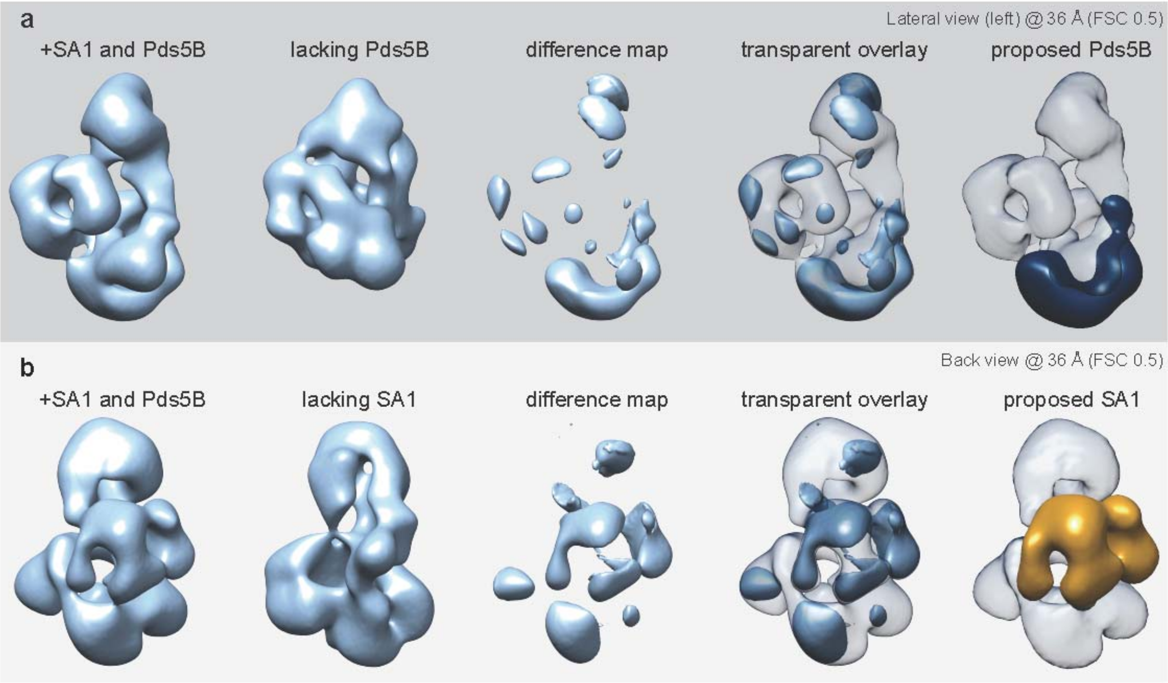

Supplementary Figure 11 Density assignment and the topology of engineered cohesin complexes. (**a**) Difference mapping between bonsai cohesin tetramers with and without Pds5B. Aligned and equally down-filtered versions of bonsai cohesion tetramer with and without Pds5B are normalized and subtracted. The most prominent different density is proposed to correspond to Pds5B. (**b**) Difference mapping between Pds5B-bound bonsai cohesin complexes with and without SA1. Aligned and equally down-filtered versions of both models are normalized and subtracted. The most prominent difference was identified in the backside clasp and proposed to correspond to SA1. The remaining density in the clasp-region in the absence of SA1 (termed clasp fragment in **Fig. 3f**) probably contains the region of Scc1 ($Scc1^{middle}$) that binds to SA1.

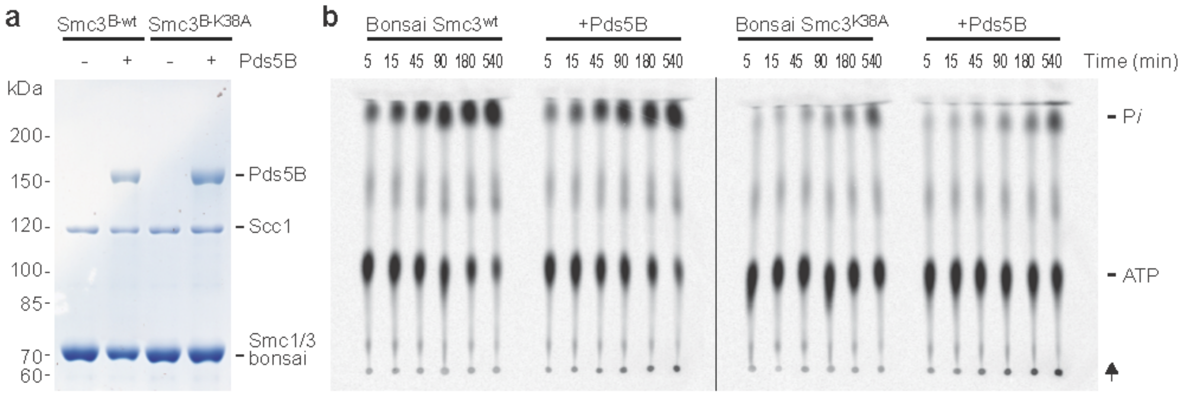

Supplementary Figure 12 Pds5 does not directly modulate cohesin's ATPase activity.

(**a**) Pds5B was bound to bonsai trimers containing $Smc3^{B}$ or $Smc3^{B\text{-}K38A}$ and analyzed by Coomassie staining after SD-PAGE. (**b**) Reaction mixtures including radiolabeled γ-[^{32}P]-ATP and equal amounts of cohesin were incubated for the times indicated. Thin layer chromatography was used to separate γ-[^{32}P]-ATP and the released [^{32}P]-P_i. The Smc3 K38A largely abolished bonsai cohesin's ATPase activity. The presence of Pds5 did not influence cohesin's enzymatic activity.

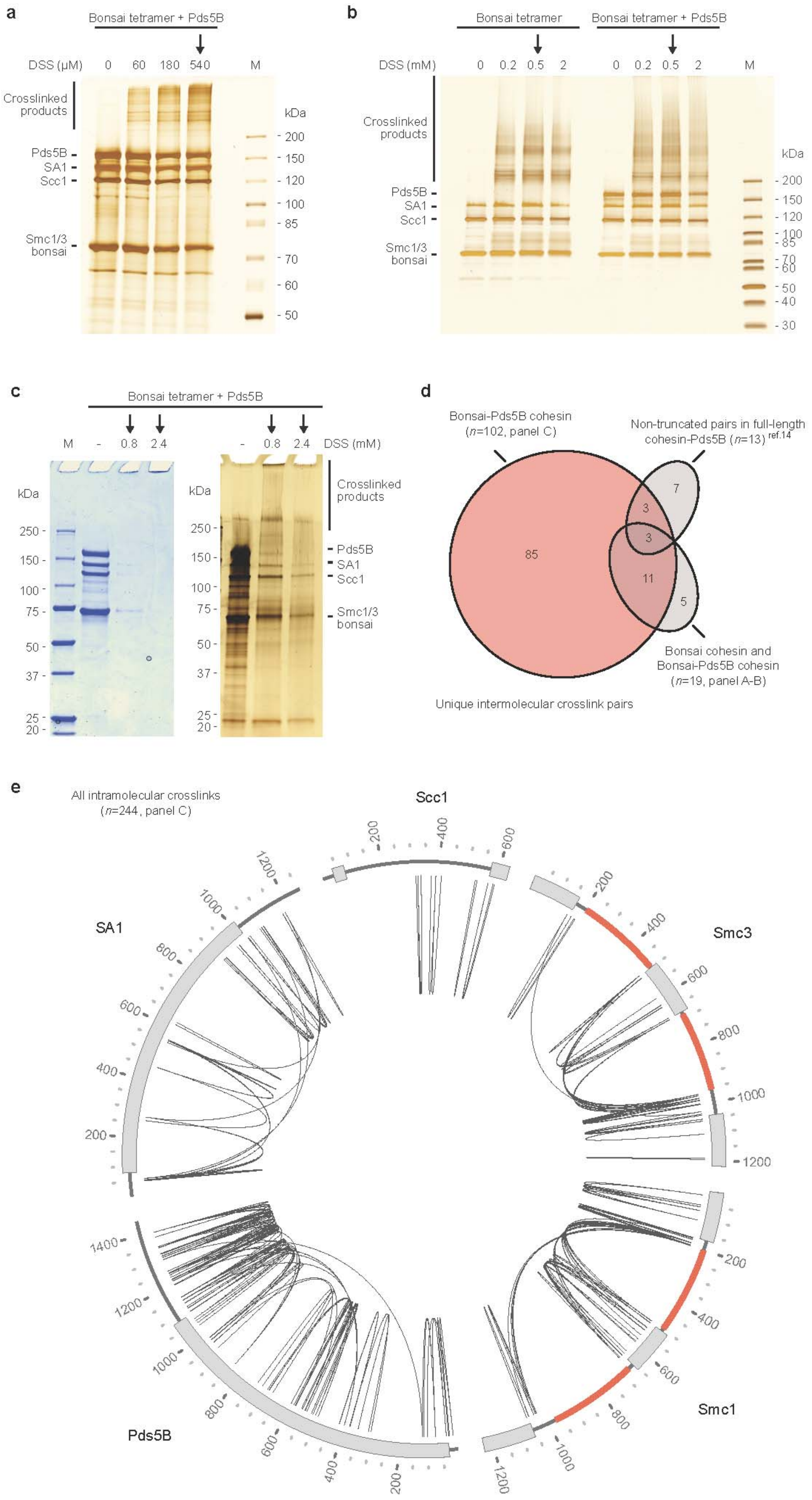
a
Bonsai tetramer + Pds5B
DSS (µM) 0 60 180 540 M
Crosslinked products
Pds5B
SA1
Scc1
Smc1/3 bonsai
kDa
200
150
120
100
85
70
60
50
b
Bonsai tetramer
Bonsai tetramer + Pds5B
DSS (mM) 0 0.2 0.5 2 0 0.2 0.5 2 M
c
Bonsai tetramer + Pds5B
M - 0.8 2.4 - 0.8 2.4 DSS (mM)
d
Bonsai-Pds5B cohesin (n=102, panel C)
Non-truncated pairs in full-length cohesin-Pds5B (n=13) ref.14
85
3
3
11
7
5
Bonsai cohesin and Bonsai-Pds5B cohesin (n=19, panel A-B)
Unique intermolecular crosslink pairs
e
All intramolecular crosslinks (n=244, panel C)
Scc1
SA1
Smc3
Smc1
Pds5B

Supplementary Figure 13 Overview of crosslinking experiments and intramolecular crosslinks

(**a, b**) Overview of crosslinking reactions performed at 4 degrees. Fractions of the cross-linked material was analyzed by SDS-PAGE for the presence of crosslinked cohesin products. The arrows indicate samples analyzed by mass spectrometry. (**c**) An increase in protein amounts and in reaction temperature increased the amount of crosslinked material. When analyzed by mass spectrometry, these samples provided a much more complete proximity map. (**d**) The unique intermolecular crosslinks of the different datasets are compared in a Venn diagram. The 102 crosslinks from the samples in panel c were used for further analysis. (**e**) A Circos plot of the 244 intramolecular crosslinks that were identified in bonsai cohesin bound to Pds5B. The truncated regions in $Smc1^{B}$ and $Smc3^{B}$ are shown in orange. Grey boxes illustrate NBDs and hinge domains of Smc1 and Smc3, alpha-helical regions in Scc1 and the alpha-helical repeats of SA1 and Pds5B.

a

biologically interesting protein-protein interfaces			
Panel	Suppl. Table 1 no.	Id	uxID
b	49	KAKYLLADCNEAFIK-EKINELLK-a3-b2	Scc1:72:x:Smc3_bonsai_FLAG:188
c	71	NKVSHIDVITAEMAK-LVQEQKPKGSQR-a2-b6	PDS5B:1242:x:Smc3_bonsai_FLAG:640
d	77	MSVNSGSSSSKTSSVR-KLIVDSVK-a11-b1	SA1:1071:x:Scc1:323
e	82	ITDGSPSKEDLLVLR-KGGEADNLDEFLK-a8-b1	SA1:759:x:Scc1:406
f	82	ITDGSPSKEDLLVLRK-KGGEADNLDEFLK-a8-b1	SA1:759:x:Scc1:406
g	83	KGGEADNLDEFLK-QIDKIQCAK-a1-b4	SA1:916:x:Scc1:406
h	84	FALTFGLDQIKTR-KGGEADNLDEFLK-a11-b1	SA1:969:x:Scc1:406
i	84	RFALTFGLDQIKTR-KGGEADNLDEFLK-a12-b1	SA1:969:x:Scc1:406
j	87	LLKLFTR-SKQAATK-a3-b2	PDS5B:1397:x:Scc1:387

b

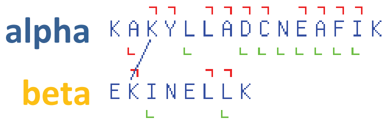

Protein1	Protein2	AbsPos1	AbsPos2	Mz	z	Error_rel[ppm]	ld-Score
SCC1	SMC3_B	72	188	582.326	5	2.2	30.94

type	position	ion th	peak	delta mz	delta ppm	intensity
alpha_common_y_standard_plus1	2	260.197	260.125	0.073	280	4.100
alpha_common_y_standard_plus1	3	407.266	407.255	0.011	28	16.334
alpha_common_y_standard_plus1	4	478.303	478.283	0.020	41	53.649
alpha_common_y_standard_plus1	5	607.346	607.225	0.121	199	23.848
alpha_common_y_standard_plus1	6	721.388	721.292	0.097	134	25.366
alpha_common_y_standard_plus1	8	996.446	996.333	0.113	113	3.451
alpha_common_y_standard_plus2	11	647.330	647.353	-0.024	-36	5.473
alpha_common_y_standard_plus2	6	361.198	361.334	-0.136	-376	5.145
alpha_common_y_standard_plus2	7	441.213	441.286	-0.073	-165	9.927
alpha_common_y_standard_plus3	6	241.135	241.074	0.061	252	0.616
alpha_xlink_b_standard_plus2	3	726.446	726.346	0.100	137	11.529
alpha_xlink_b_standard_plus2	4	807.977	808.080	-0.103	-127	12.808
alpha_xlink_b_standard_plus3	12	834.449	834.583	-0.133	-160	28.243
alpha_xlink_b_standard_plus3	13	883.472	883.756	-0.284	-321	37.796
alpha_xlink_b_standard_plus3	6	614.377	614.591	-0.214	-349	53.155
alpha_xlink_b_standard_plus3	7	638.056	638.148	-0.092	-143	43.901
alpha_xlink_b_standard_plus3	8	676.398	676.683	-0.284	-420	65.036
alpha_xlink_b_standard_plus3	9	729.742	729.743	-0.002	-2	32.173
alpha_xlink_b_standard_plus4	11	608.330	608.561	-0.231	-380	100.000
alpha_xlink_b_standard_plus4	12	626.089	626.004	0.085	136	13.445
alpha_xlink_b_standard_plus4	13	662.856	663.079	-0.223	-337	29.872
alpha_xlink_b_standard_plus4	7	478.794	479.120	-0.326	-681	21.856
alpha_xlink_b_standard_plus4	8	507.551	507.882	-0.332	-653	14.325
alpha_xlink_b_standard_plus4	9	547.558	547.849	-0.290	-530	25.710
alpha_xlink_b_standard_plus5	14	553.103	553.566	-0.463	-837	18.488
alpha_xlink_y_standard_plus5	14	556.705	557.096	-0.390	-701	14.711
beta_common_y_standard_plus1	2	260.197	260.125	0.073	280	4.100
beta_common_y_standard_plus2	6	365.229	365.286	-0.056	-154	2.866
beta_xlink_b_standard_plus3	2	727.054	727.328	-0.274	-376	9.529
beta_xlink_b_standard_plus3	6	883.472	883.756	-0.284	-321	37.796
beta_xlink_b_standard_plus4	6	662.856	663.079	-0.223	-337	29.872
beta_xlink_b_standard_plus5	7	553.103	553.566	-0.463	-837	18.488

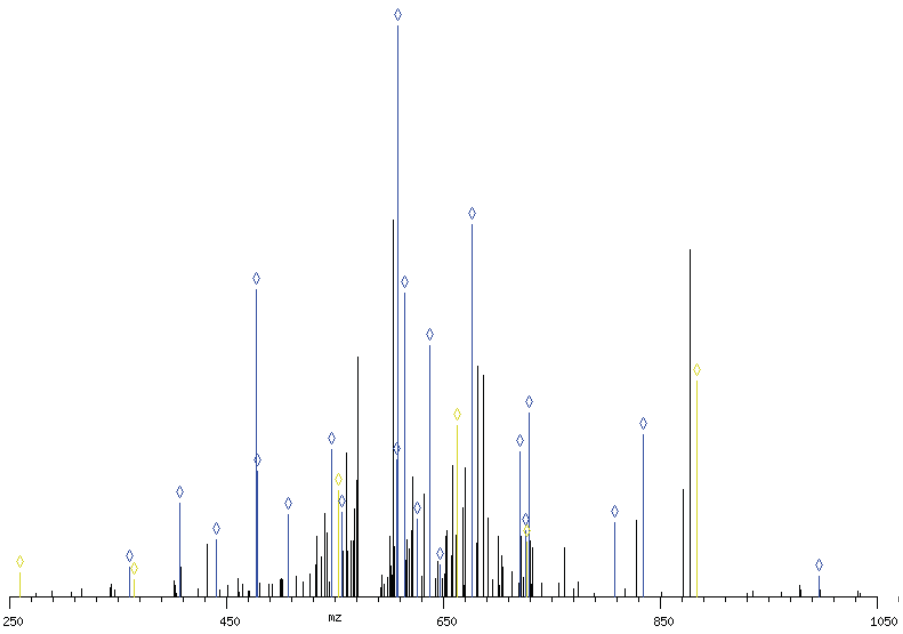

C

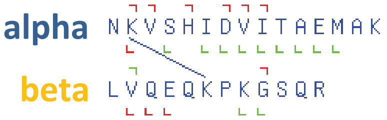

Protein1	Protein2	AbsPos1	AbsPos2	Mz	z	Error_rel[ppm]	Id-Score
SMC3_B	PDS5B	640	1242	638.952	5	0.9	33.7

type	position	ion th	peak	delta mz	delta ppm	intensity
alpha_common_y_standard_plus1	10	1090.582	1090.584	-0.003	-2	1.165
alpha_common_y_standard_plus1	3	349.191	349.325	-0.134	-384	1.884
alpha_common_y_standard_plus1	4	478.234	478.254	-0.020	-43	4.691
alpha_common_y_standard_plus1	5	549.271	549.346	-0.076	-138	7.797
alpha_common_y_standard_plus1	6	650.318	650.317	0.002	2	26.319
alpha_common_y_standard_plus1	7	763.402	763.456	-0.054	-70	8.419
alpha_common_y_standard_plus1	8	862.471	862.475	-0.004	-5	3.091
alpha_common_y_standard_plus1	9	977.498	977.541	-0.043	-44	3.417
alpha_common_y_standard_plus2	12	657.840	657.961	-0.120	-183	8.890
alpha_common_y_standard_plus2	6	325.663	325.753	-0.090	-275	3.527
alpha_xlink_b_standard_plus3	2	593.336	593.566	-0.230	-388	42.367
alpha_xlink_b_standard_plus3	3	626.359	626.503	-0.144	-230	9.644
alpha_xlink_b_standard_plus3	5	701.056	701.561	-0.505	-720	7.186
alpha_xlink_b_standard_plus3	7	777.093	777.317	-0.224	-288	28.307
alpha_xlink_b_standard_plus3	8	810.116	810.483	-0.367	-453	100.000
alpha_xlink_b_standard_plus3	9	847.810	848.117	-0.307	-362	28.921
alpha_xlink_b_standard_plus4	7	583.072	583.268	-0.196	-337	30.031
alpha_xlink_b_standard_plus4	8	607.839	608.176	-0.338	-555	22.729
alpha_xlink_y_standard_plus4	14	769.927	770.141	-0.214	-278	6.225
alpha_xlink_y_standard_plus5	14	616.143	616.457	-0.314	-510	6.079
beta_common_b_standard_plus1	2	213.160	213.193	-0.033	-155	2.066
beta_common_y_standard_plus1	4	447.232	447.311	-0.080	-178	1.042
beta_common_y_standard_plus1	5	575.327	575.242	0.084	147	1.001
beta_xlink_b_standard_plus4	9	701.137	701.561	-0.424	-605	7.186
beta_xlink_y_standard_plus4	10	745.399	745.693	-0.294	-395	58.143
beta_xlink_y_standard_plus4	11	770.166	770.141	0.025	33	6.225
beta_xlink_y_standard_plus4	9	713.385	713.636	-0.251	-352	11.752
beta_xlink_y_standard_plus5	10	596.521	596.836	-0.315	-528	14.880
beta_xlink_y_standard_plus5	11	616.335	616.457	-0.123	-199	6.079

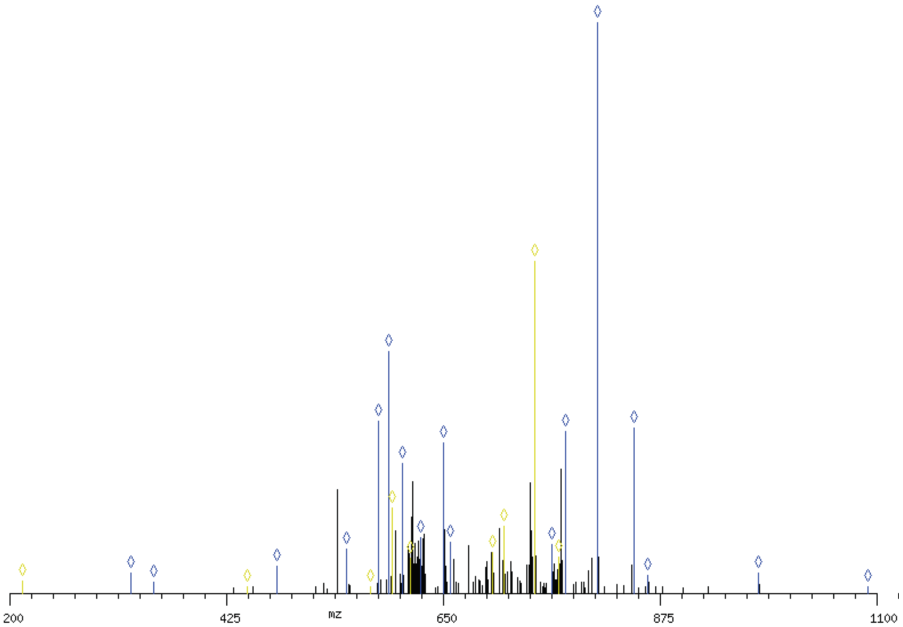

d

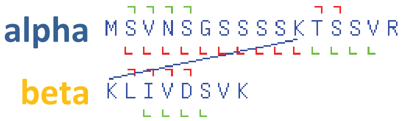

Protein1	Protein2	AbsPos1	AbsPos2	Mz	z	Error_rel[ppm]	ld-Score
SA1	SCC1	1071	323	660.604	4	-0.1	35.48

type	position	ion th	peak	delta mz	delta ppm	intensity
alpha_common_b_standard_plus1	2	219.080	219.095	-0.015	-69	2.826
alpha_common_b_standard_plus1	3	318.149	318.235	-0.086	-271	2.073
alpha_common_b_standard_plus1	4	432.192	432.156	0.036	84	6.881
alpha_common_b_standard_plus1	5	519.224	519.260	-0.036	-69	5.151
alpha_common_y_standard_plus1	2	274.188	274.204	-0.017	-60	4.281
alpha_common_y_standard_plus1	3	361.220	361.248	-0.028	-76	8.476
alpha_common_y_standard_plus1	4	448.252	448.220	0.032	71	21.165
alpha_common_y_standard_plus1	5	549.300	549.344	-0.045	-82	18.523
alpha_xlink_b_standard_plus3	12	731.388	731.749	-0.361	-494	7.704
alpha_xlink_b_standard_plus3	13	760.399	760.996	-0.597	-785	6.320
alpha_xlink_y_standard_plus2	12	1104.608	1104.757	-0.149	-134	6.955
alpha_xlink_y_standard_plus2	7	902.033	902.251	-0.217	-241	19.491
alpha_xlink_y_standard_plus2	8	945.549	945.733	-0.184	-195	9.395
alpha_xlink_y_standard_plus2	9	989.066	989.155	-0.090	-91	11.196
alpha_xlink_y_standard_plus3	10	688.724	688.785	-0.061	-89	20.410
alpha_xlink_y_standard_plus3	11	707.731	707.959	-0.228	-323	90.520
alpha_xlink_y_standard_plus3	12	736.741	737.013	-0.272	-369	74.413
alpha_xlink_y_standard_plus3	13	774.756	774.994	-0.238	-307	100.000
alpha_xlink_y_standard_plus3	14	807.779	808.005	-0.227	-281	78.300
alpha_xlink_y_standard_plus3	15	836.789	837.025	-0.236	-282	62.185
alpha_xlink_y_standard_plus3	6	572.681	572.974	-0.293	-511	13.081
alpha_xlink_y_standard_plus3	7	601.692	601.949	-0.257	-428	53.573
alpha_xlink_y_standard_plus3	8	630.702	630.817	-0.115	-182	24.018
alpha_xlink_y_standard_plus4	14	606.086	606.381	-0.295	-486	29.787
alpha_xlink_y_standard_plus4	15	627.844	628.137	-0.293	-467	5.906
beta_common_y_standard_plus1	3	333.214	333.130	0.084	251	7.348
beta_common_y_standard_plus1	4	448.241	448.220	0.021	46	21.165
beta_common_y_standard_plus1	5	547.309	547.314	-0.005	-9	15.569
beta_common_y_standard_plus2	5	274.158	274.204	-0.046	-168	4.281
beta_common_y_standard_plus2	6	330.701	330.697	0.003	11	3.442
beta_xlink_b_standard_plus2	2	990.508	990.260	0.247	250	7.028
beta_xlink_b_standard_plus3	3	698.369	698.374	-0.005	-7	12.116
beta_xlink_b_standard_plus3	4	731.392	731.749	-0.357	-489	7.704
beta_xlink_b_standard_plus3	5	769.734	769.801	-0.067	-86	17.549

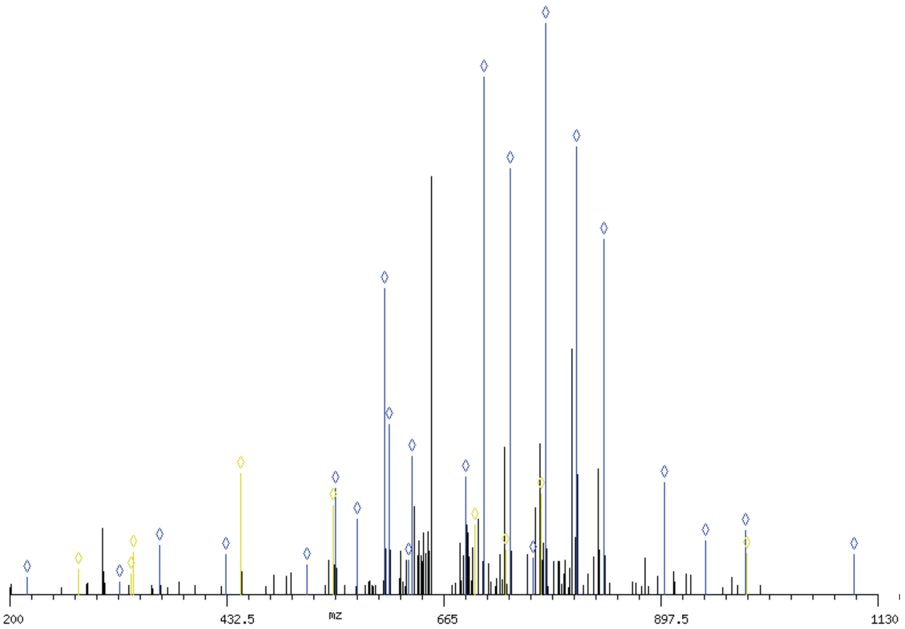

e

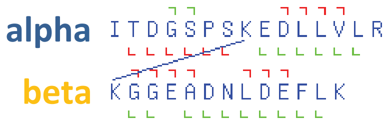

Protein1	Protein2	AbsPos1	AbsPos2	Mz	z	Error_rel[ppm]	Id-Score
SA1	SCC1	759	406	804.673	4	-0.4	39.97

type	position	ion th	peak	delta mz	delta ppm	intensity
alpha_common_b_standard_plus1	4	387.188	387.302	-0.114	-295	6.305
alpha_common_b_standard_plus1	5	474.220	474.302	-0.082	-172	3.889
alpha_common_y_standard_plus1	2	288.204	288.221	-0.018	-62	4.380
alpha_common_y_standard_plus1	3	387.272	387.302	-0.030	-78	6.305
alpha_common_y_standard_plus1	4	500.356	500.430	-0.074	-147	6.835
alpha_common_y_standard_plus1	5	613.440	613.403	0.037	60	6.256
alpha_common_y_standard_plus1	6	728.467	728.467	0.000	0	8.180
alpha_common_y_standard_plus1	7	857.510	857.534	-0.025	-29	12.582
alpha_xlink_b_standard_plus3	10	868.417	868.669	-0.252	-290	5.175
alpha_xlink_b_standard_plus3	11	906.112	905.955	0.157	173	4.144
alpha_xlink_b_standard_plus3	12	943.807	943.805	0.002	2	10.951
alpha_xlink_b_standard_plus3	13	976.830	977.462	-0.632	-647	3.870
alpha_xlink_b_standard_plus4	10	651.565	651.363	0.202	310	14.333
alpha_xlink_y_standard_plus3	10	914.824	915.125	-0.301	-328	100.000
alpha_xlink_y_standard_plus3	11	943.835	943.805	0.030	31	10.951
alpha_xlink_y_standard_plus3	12	962.842	963.255	-0.413	-429	12.144
alpha_xlink_y_standard_plus3	13	1001.184	1001.517	-0.333	-332	55.831
alpha_xlink_y_standard_plus3	14	1034.867	1035.337	-0.470	-454	13.087
alpha_xlink_y_standard_plus3	9	882.473	882.856	-0.383	-434	3.115
alpha_xlink_y_standard_plus4	13	751.140	751.542	-0.402	-535	4.320
alpha_xlink_y_standard_plus4	14	776.402	776.780	-0.378	-487	12.467
beta_common_y_standard_plus1	11	1250.590	1250.552	0.038	31	2.101
beta_common_y_standard_plus1	12	1307.612	1307.612	-0.000	-0	2.687
beta_common_y_standard_plus1	2	260.197	260.246	-0.048	-186	1.476
beta_common_y_standard_plus1	3	407.266	407.326	-0.060	-147	2.102
beta_common_y_standard_plus1	4	536.308	536.407	-0.098	-184	2.348
beta_common_y_standard_plus1	5	651.335	651.363	-0.028	-42	14.333
beta_common_y_standard_plus1	6	764.419	764.461	-0.042	-55	9.767
beta_common_y_standard_plus1	7	878.462	878.420	0.042	48	13.792
beta_common_y_standard_plus1	8	993.489	993.490	-0.001	-1	20.069
beta_common_y_standard_plus1	9	1064.526	1064.477	0.049	46	18.606
beta_common_y_standard_plus2	12	654.310	654.471	-0.162	-247	1.720
beta_xlink_b_standard_plus2	2	983.547	983.784	-0.237	-241	8.174
beta_xlink_b_standard_plus2	3	1012.058	1012.586	-0.528	-522	5.994
beta_xlink_b_standard_plus2	4	1076.579	1076.872	-0.293	-272	11.185
beta_xlink_b_standard_plus3	10	937.142	937.542	-0.399	-426	5.645
beta_xlink_b_standard_plus3	4	718.055	718.393	-0.338	-471	3.453
beta_xlink_b_standard_plus3	5	741.734	741.694	0.041	55	4.859
beta_xlink_b_standard_plus3	8	855.786	856.101	-0.315	-368	8.056
beta_xlink_b_standard_plus3	9	894.128	894.620	-0.492	-551	6.041

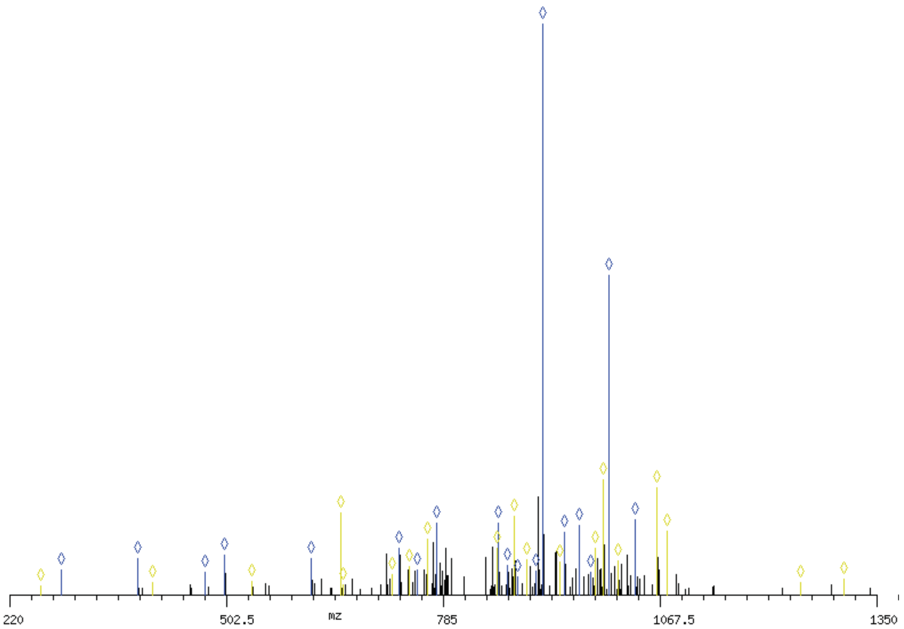

f

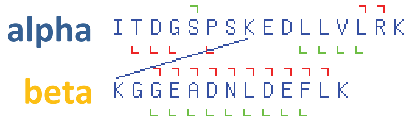

Protein1	Protein2	AbsPos1	AbsPos2	Mz	z	Error_rel[ppm]	Id-Score
SA1	SCC1	759	406	836.697	4	-0.3	33.79

type	position	ion th	peak	delta mz	delta ppm	intensity
alpha_common_b_standard_plus1	5	474.220	474.228	-0.008	-16	5.047
alpha_common_y_standard_plus1	3	416.299	416.328	-0.030	-72	5.258
alpha_common_y_standard_plus1	4	515.367	515.349	0.018	35	8.473
alpha_common_y_standard_plus1	5	628.451	628.602	-0.151	-241	7.073
alpha_common_y_standard_plus1	6	741.535	741.436	0.099	133	23.009
alpha_xlink_b_standard_plus3	15	1066.558	1066.819	-0.262	-245	85.151
alpha_xlink_b_standard_plus4	14	761.145	761.582	-0.437	-575	32.850
alpha_xlink_y_standard_plus3	11	957.522	957.896	-0.373	-390	64.190
alpha_xlink_y_standard_plus3	13	1005.540	1005.804	-0.264	-262	25.318
alpha_xlink_y_standard_plus3	14	1043.883	1044.255	-0.372	-357	92.813
alpha_xlink_y_standard_plus3	15	1077.565	1077.876	-0.311	-288	58.091
alpha_xlink_y_standard_plus4	14	783.164	783.543	-0.379	-484	20.281
alpha_xlink_y_standard_plus4	15	808.426	808.811	-0.386	-477	18.333
beta_common_y_standard_plus1	10	1193.569	1193.682	-0.113	-95	4.529
beta_common_y_standard_plus1	11	1250.590	1250.658	-0.067	-54	5.931
beta_common_y_standard_plus1	3	407.266	407.263	0.003	7	5.696
beta_common_y_standard_plus1	4	536.308	536.394	-0.086	-160	19.598
beta_common_y_standard_plus1	5	651.335	651.360	-0.024	-38	65.897
beta_common_y_standard_plus1	6	764.419	764.455	-0.036	-47	42.050
beta_common_y_standard_plus1	7	878.462	878.529	-0.067	-76	64.983
beta_common_y_standard_plus1	8	993.489	993.306	0.183	184	87.704
beta_common_y_standard_plus1	9	1064.526	1064.502	0.024	23	61.589
beta_common_y_standard_plus2	11	625.799	625.974	-0.175	-280	9.679
beta_xlink_b_standard_plus2	3	1076.105	1076.857	-0.752	-698	16.132
beta_xlink_b_standard_plus2	4	1140.626	1140.992	-0.366	-321	41.718
beta_xlink_b_standard_plus3	10	979.840	980.445	-0.604	-617	32.459
beta_xlink_b_standard_plus3	11	1028.863	1029.147	-0.283	-276	24.852
beta_xlink_b_standard_plus3	12	1066.558	1066.819	-0.262	-245	85.151
beta_xlink_b_standard_plus3	4	760.754	760.816	-0.062	-82	100.000
beta_xlink_b_standard_plus3	5	784.433	784.993	-0.560	-714	68.775
beta_xlink_b_standard_plus3	6	822.775	822.972	-0.197	-240	62.022
beta_xlink_b_standard_plus3	7	860.789	860.864	-0.075	-87	23.967
beta_xlink_b_standard_plus3	8	898.484	898.828	-0.344	-383	53.801
beta_xlink_b_standard_plus3	9	936.826	936.798	0.029	30	31.911

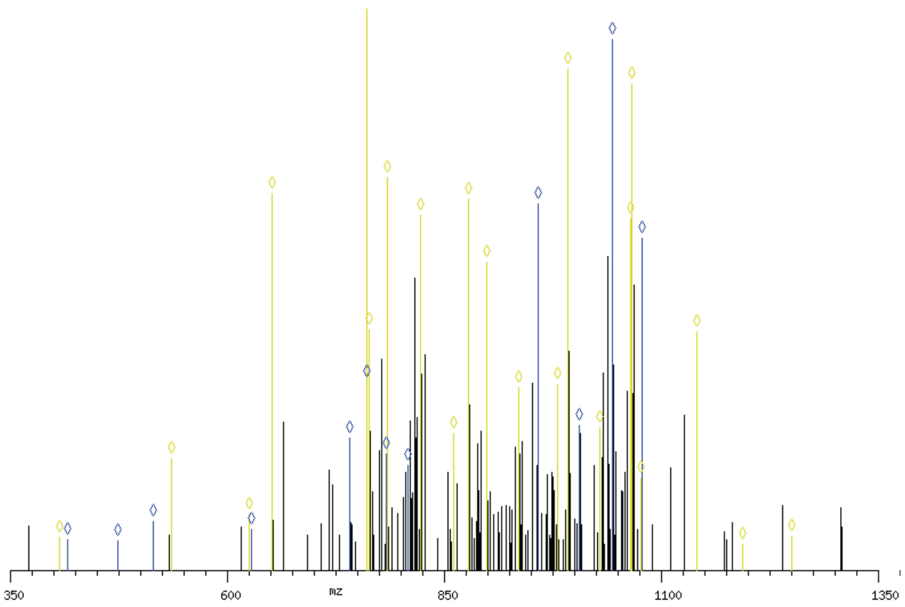

g

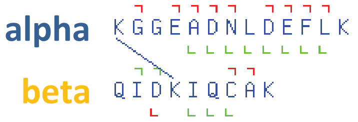

Protein1	Protein2	AbsPos1	AbsPos2	Mz	z	Error_rel[ppm]	Id-Score
SCC1	SA1	406	916	669.844	4	-1.8	35.48

type	position	ion th	peak	delta mz	delta ppm	intensity
alpha_common_y_standard_plus1	2	260.197	260.265	-0.068	-260	2.540
alpha_common_y_standard_plus1	3	407.266	407.285	-0.019	-48	6.169
alpha_common_y_standard_plus1	4	536.308	536.277	0.032	59	14.406
alpha_common_y_standard_plus1	5	651.335	651.331	0.004	6	100.000
alpha_common_y_standard_plus1	6	764.419	764.403	0.016	21	37.998
alpha_common_y_standard_plus1	7	878.462	878.443	0.020	22	23.974
alpha_common_y_standard_plus1	8	993.489	993.497	-0.007	-7	23.716
alpha_common_y_standard_plus1	9	1064.526	1064.466	0.060	56	13.809
alpha_common_y_standard_plus2	8	497.249	497.122	0.126	254	3.537
alpha_common_y_standard_plus2	9	532.767	532.722	0.045	84	36.649
alpha_xlink_b_standard_plus2	2	713.890	713.843	0.047	66	8.974
alpha_xlink_b_standard_plus2	4	806.922	806.962	-0.040	-49	51.111
alpha_xlink_b_standard_plus2	6	899.954	899.854,900.643	0.101,-0.688	112,-765	9.653,13.797
alpha_xlink_b_standard_plus3	10	757.371	757.544	-0.173	-228	16.178
alpha_xlink_b_standard_plus3	11	806.394	806.302,806.962	0.092,-0.568	114,-705	16.917,51.111
alpha_xlink_b_standard_plus3	12	844.089	844.425	-0.336	-398	37.405
alpha_xlink_b_standard_plus3	4	538.284	538.298	-0.014	-27	13.903
alpha_xlink_b_standard_plus3	5	561.963	562.238	-0.275	-489	32.564
alpha_xlink_b_standard_plus3	6	600.306	600.291	0.014	23	19.373
alpha_xlink_b_standard_plus3	7	638.320	638.553	-0.234	-366	49.808
alpha_xlink_b_standard_plus3	9	714.357	714.540	-0.184	-257	29.622
beta_common_b_standard_plus1	2	242.150	242.174	-0.024	-98	2.281
beta_common_b_standard_plus1	3	357.177	357.151	0.027	74	2.087
beta_common_y_standard_plus1	3	378.181	378.150	0.031	83	12.503
beta_common_y_standard_plus1	4	506.240	506.223	0.017	34	29.814
beta_common_y_standard_plus1	5	619.324	619.286	0.037	60	19.695
beta_xlink_b_standard_plus3	7	820.409	820.119	0.290	354	10.718
beta_xlink_b_standard_plus3	8	844.089	844.425	-0.336	-398	37.405
beta_xlink_b_standard_plus4	7	615.559	615.360	0.199	323	9.570
beta_xlink_y_standard_plus3	7	812.409	812.491	-0.082	-100	23.916

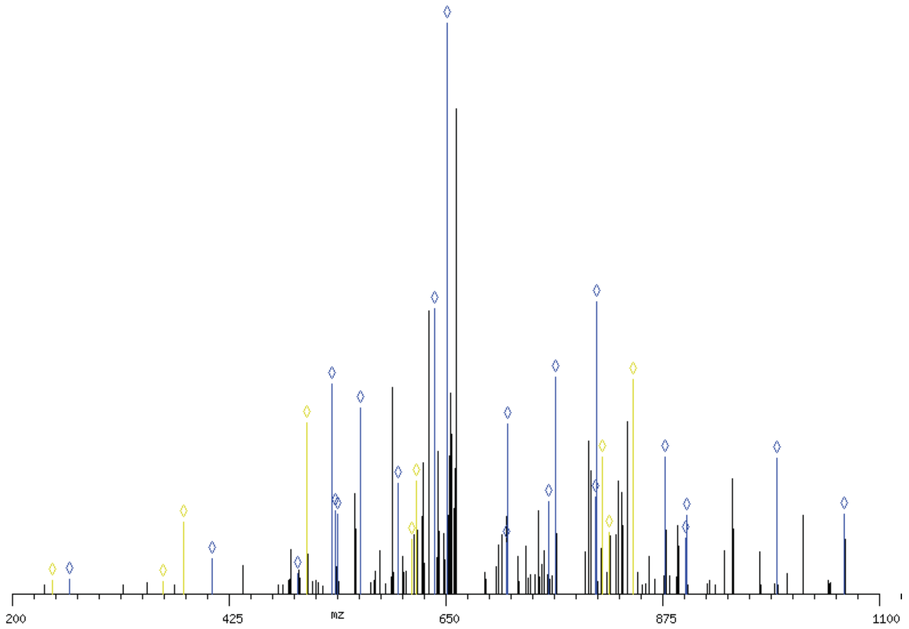

h

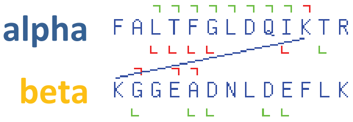

Protein1	Protein2	AbsPos1	AbsPos2	Mz	z	Error_rel[ppm]	Id-Score
SA1	SCC1	969	406	771.408	4	-0.6	29.5

type	position	ion th	peak	delta mz	delta ppm	intensity
alpha_common_b_standard_plus1	10	1106.589	1106.772	-0.183	-165	7.801
alpha_common_b_standard_plus1	3	332.197	332.152	0.046	138	4.342
alpha_common_b_standard_plus1	4	433.245	433.273	-0.028	-64	5.333
alpha_common_b_standard_plus1	5	580.314	580.378	-0.065	-111	1.413
alpha_common_b_standard_plus1	6	637.335	637.495	-0.161	-252	2.673
alpha_common_b_standard_plus1	7	750.419	750.522	-0.103	-138	17.161
alpha_common_b_standard_plus1	9	993.505	993.563	-0.059	-59	16.744
alpha_common_b_standard_plus2	8	433.227	433.273	-0.046	-106	5.333
alpha_common_y_standard_plus1	2	276.167	276.151	0.017	60	2.667
alpha_xlink_b_standard_plus3	11	936.489	936.580	-0.092	-98	10.944
alpha_xlink_y_standard_plus3	10	917.812	918.137	-0.325	-354	100.000
alpha_xlink_y_standard_plus3	11	955.507	955.859	-0.353	-369	49.788
alpha_xlink_y_standard_plus3	4	697.376	697.628	-0.251	-361	14.366
alpha_xlink_y_standard_plus3	8	835.107	835.150	-0.043	-51	39.734
alpha_xlink_y_standard_plus3	9	884.129	884.378	-0.249	-281	83.431
alpha_xlink_y_standard_plus4	11	716.882	717.075	-0.193	-269	15.679
beta_common_y_standard_plus1	12	1307.612	1307.544	0.068	52	1.391
beta_common_y_standard_plus1	4	536.308	536.384	-0.076	-142	5.202
beta_common_y_standard_plus1	5	651.335	651.367	-0.032	-48	14.920
beta_common_y_standard_plus1	8	993.489	993.563	-0.074	-75	16.744
beta_common_y_standard_plus1	9	1064.526	1064.591	-0.065	-61	6.992
beta_common_y_standard_plus2	9	532.767	532.747	0.020	37	2.553
beta_xlink_b_standard_plus2	2	917.018	917.035	-0.017	-19	13.323
beta_xlink_b_standard_plus2	4	1010.050	1010.381	-0.331	-328	10.940
beta_xlink_b_standard_plus3	2	611.681	611.492	0.190	310	7.761
beta_xlink_b_standard_plus3	5	697.381	697.628	-0.246	-353	14.366

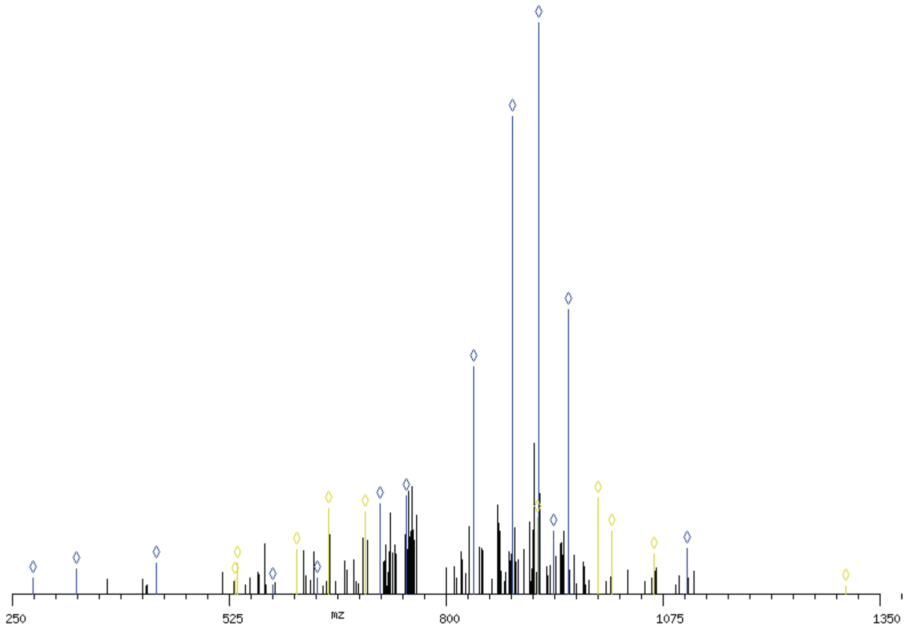

i

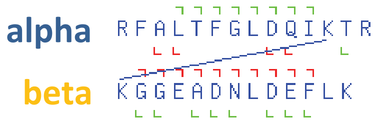

Protein1	Protein2	AbsPos1	AbsPos2	Mz	z	Error_rel[ppm]	Id-Score
SA1	SCC1	969	406	810.434	4	0.8	34.31

type	position	ion th	peak	delta mz	delta ppm	intensity
alpha_common_b_standard_plus1	10	1149.606	1149.755	-0.149	-130	17.695
alpha_common_b_standard_plus1	11	1262.690	1262.739	-0.050	-39	10.386
alpha_common_b_standard_plus1	4	488.299	488.400	-0.102	-209	6.255
alpha_common_b_standard_plus1	5	589.346	589.491	-0.145	-245	11.243
alpha_common_b_standard_plus1	6	736.415	736.569	-0.155	-210	10.999
alpha_common_b_standard_plus1	7	793.436	793.422	0.014	18	37.111
alpha_common_b_standard_plus1	8	906.520	906.591	-0.071	-78	17.585
alpha_common_b_standard_plus1	9	1021.547	1021.585	-0.037	-37	25.213
alpha_common_b_standard_plus2	9	511.277	511.279	-0.001	-2	8.922
alpha_common_y_standard_plus1	2	276.167	276.174	-0.007	-27	7.307
alpha_xlink_y_standard_plus2	5	1109.590	1109.865	-0.275	-248	13.763
alpha_xlink_y_standard_plus3	6	778.405	778.799	-0.394	-506	30.634
alpha_xlink_y_standard_plus4	11	716.882	716.594	0.288	402	19.957
alpha_xlink_y_standard_plus4	12	734.641	734.464	0.178	242	37.877
beta_common_y_standard_plus1	11	1250.590	1250.708	-0.117	-94	11.606
beta_common_y_standard_plus1	12	1307.612	1307.720	-0.108	-83	10.123
beta_common_y_standard_plus1	3	407.266	407.326	-0.061	-149	9.068
beta_common_y_standard_plus1	4	536.308	536.428	-0.119	-223	9.961
beta_common_y_standard_plus1	5	651.335	651.398	-0.063	-97	43.157
beta_common_y_standard_plus1	7	878.462	878.508	-0.045	-52	50.149
beta_common_y_standard_plus1	8	993.489	993.598	-0.109	-110	54.715
beta_common_y_standard_plus1	9	1064.526	1064.555	-0.029	-27	48.269
beta_xlink_b_standard_plus2	2	995.068	995.780	-0.712	-716	42.086
beta_xlink_b_standard_plus2	3	1023.579	1023.837	-0.258	-252	21.419
beta_xlink_b_standard_plus2	4	1088.100	1088.420	-0.320	-294	32.826
beta_xlink_b_standard_plus3	10	944.823	945.168	-0.346	-366	51.545
beta_xlink_b_standard_plus3	11	993.846	993.598	0.248	249	54.715
beta_xlink_b_standard_plus3	2	663.715	663.977	-0.263	-396	21.833
beta_xlink_b_standard_plus3	3	682.722	682.984	-0.262	-383	46.734
beta_xlink_b_standard_plus3	4	725.736	725.890	-0.154	-213	77.073
beta_xlink_b_standard_plus3	5	749.415	749.814	-0.399	-532	87.616
beta_xlink_b_standard_plus3	6	787.757	788.055	-0.297	-377	100.000
beta_xlink_b_standard_plus3	7	825.772	825.944	-0.172	-208	18.947
beta_xlink_b_standard_plus3	8	863.466	863.839	-0.373	-432	53.445
beta_xlink_b_standard_plus3	9	901.809	901.939	-0.130	-144	13.844

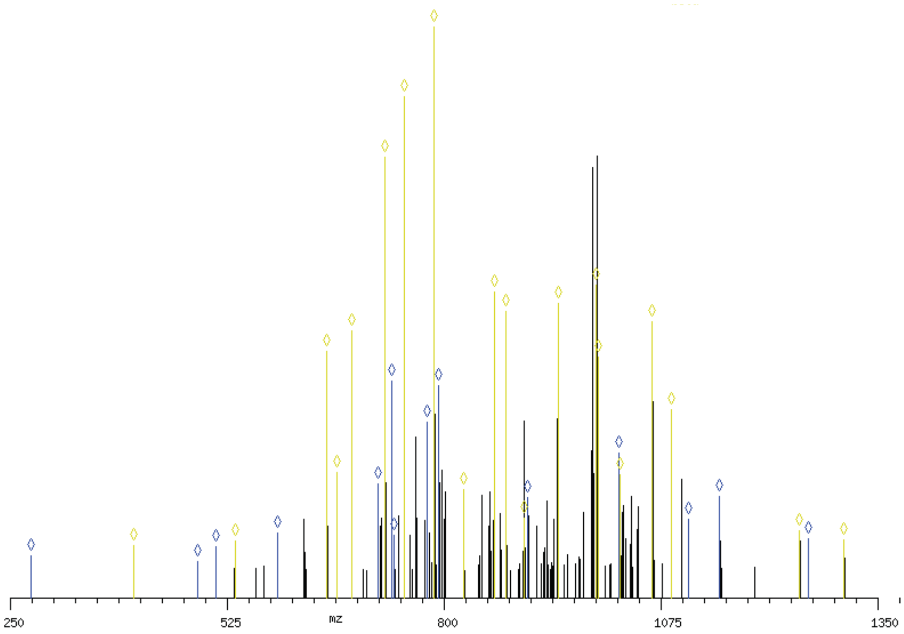

j

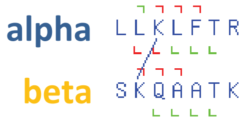

Protein1	Protein2	AbsPos1	AbsPos2	Mz	z	Error_rel[ppm]	Id-Score
SCC1	PDS5B	387	1397	441.021	4	-2.1	38.91

type	position	ion th	peak	delta mz	delta ppm	intensity
alpha_common_b_standard_plus1	2	227.176	227.132	0.044	196	6.100
alpha_common_y_standard_plus1	2	276.167	276.097	0.070	254	5.177
alpha_common_y_standard_plus1	3	423.236	423.210	0.026	61	19.382
alpha_common_y_standard_plus1	4	536.320	536.301	0.019	35	16.690
alpha_common_y_standard_plus2	4	268.664	268.730	-0.067	-248	2.167
alpha_xlink_b_standard_plus2	3	613.380	613.507	-0.127	-207	38.104
alpha_xlink_b_standard_plus3	3	409.256	409.289	-0.033	-80	37.103
alpha_xlink_b_standard_plus3	4	446.951	447.458	-0.507	-1135	1.609
alpha_xlink_b_standard_plus3	5	495.973	496.061	-0.087	-176	6.470
alpha_xlink_y_standard_plus3	5	512.304	512.469	-0.166	-323	100.000
alpha_xlink_y_standard_plus3	6	549.998	550.070	-0.072	-130	6.787
alpha_xlink_y_standard_plus4	5	384.480	384.540	-0.060	-156	13.377
beta_common_y_standard_plus1	2	248.161	248.189	-0.028	-111	0.378
beta_common_y_standard_plus1	3	319.198	319.238	-0.040	-126	1.150
beta_common_y_standard_plus1	4	390.235	390.198	0.037	96	3.753
beta_common_y_standard_plus1	5	518.294	518.230	0.064	123	16.119
beta_xlink_b_standard_plus2	2	622.393	622.571	-0.178	-286	17.654
beta_xlink_b_standard_plus3	2	415.264	415.273	-0.009	-21	7.961
beta_xlink_b_standard_plus3	3	457.951	457.782,458.437	0.169,-0.487	369,-1063	1.501,2.740
beta_xlink_b_standard_plus3	4	481.630	481.663	-0.033	-68	1.608

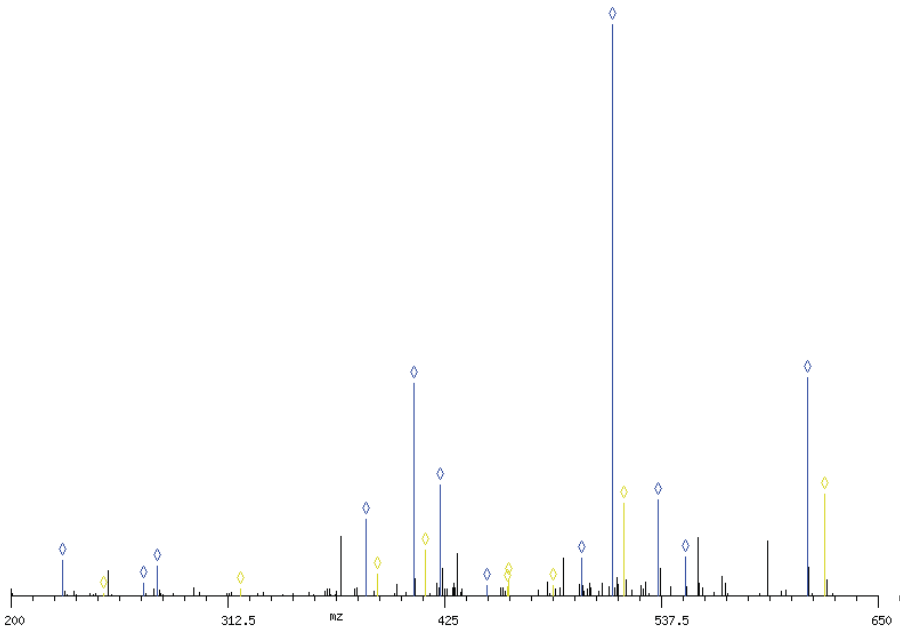

Supplementary Figure 14 Representative ms/ms spectra of high-scoring and low-scoring intermolecular crosslinks. (**a**) Nine crosslinks were selected for their occurrence at biologically interesting protein-protein interfaces. The ms/ms spectra are shown to provide insight into the spectral quality of both high-scoring and low-scoring crosslinks. The position of the particular crosslinks in **Supplementary Table 1** are indicated. (**b-j**) Every panel displays the amino acid sequences of peptide alpha and peptide beta on the top left. Linked lysines are indicated. Red edges depict the cross-link fragment ions while the green edges depict the linear fragment ions. The table on the top right provides the respective protein names, the absolute sequence positions of the indicated linked lysines, the mass over charge ratio of the precursor (m/z), the charge state (z), the relative mass error in ppm and the cross-link identification score calculated by xQuest[2]. Diamonds in the fragment ion spectrum indicate assigned fragment ions. The table on the left shows the matched cross-link (red) and linear (green) fragment ions, the charge state of the fragment ion (e.g. _plus2), the peak intensity, as well as the theoretical and experimental m/z values. All fragment ion spectra were manually evaluated as described[3].

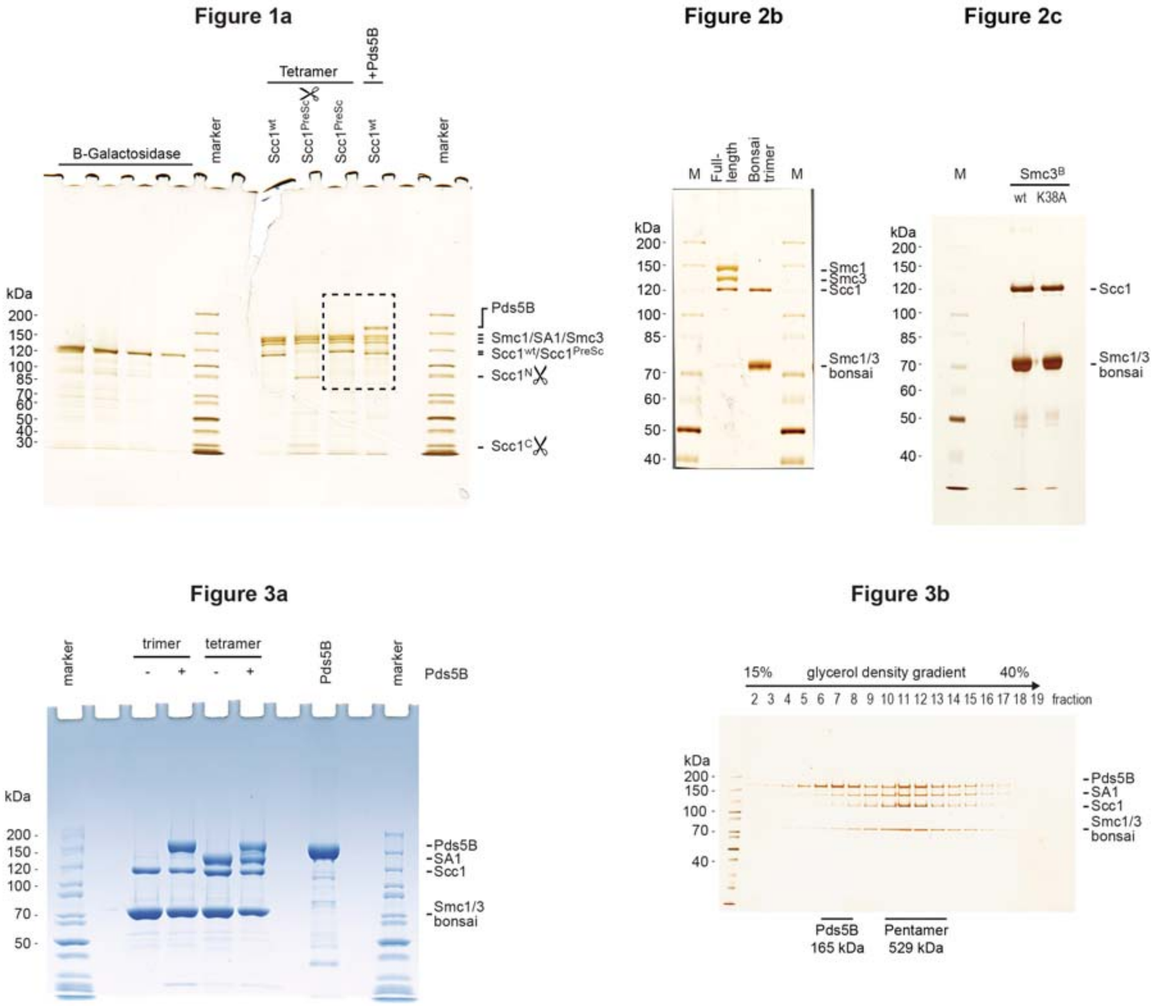

Supplementary Figure 15 Uncropped gels and blots used in the main figures

Supplementary Table 1 All peptides containing intermolecular crosslinks that were identified in Pds5B-bound bonsai cohesin. An asterisk in the column "Swap" indicates that the crosslinked peptide fragments a and b belong to protein 2 and protein 1, respectively.

	Unique peptide	Protein 1		Protein 2		Swap
1	VSFTGKQGEMR-VKTLR-a6-b2	SMC1_B	59	SMC3_B	1105	*
2	VIVGGSSEYKINNK-VSFTGKQGEMR-a10-b6	SMC1_B	106	SMC3_B	1105	
3	LHTLEGTKK-KNIAAER-a8-b1	SMC1_B	190	SMC3_B	492	*
4	KLEQCNTELKK-KNIAAER-a10-b1	SMC1_B	190	SMC3_B	977	*
5	LHTLEGTKK-KEAKPGR-a8-b1	SMC1_B	197	SMC3_B	492	*
	LHTLEGTKK-KEAKPGRK-a8-b1	SMC1_B	197	SMC3_B	492	*
6	KEAKPGR-KQQLLR-a1-b1	SMC1_B	197	SMC3_B	493	
7	AATGKAILNGIDSINK-KEAKPGR-a5-b1	SMC1_B	197	SMC3_B	503	*
8	KSRLELQK-KEAKPGR-a1-b1	SMC1_B	197	SMC3_B	673	*
9	LELQKDVR-KEAKPGR-a5-b1	SMC1_B	197	SMC3_B	680	*
	SRLELQKDVR-KEAKPGR-a7-b1	SMC1_B	197	SMC3_B	680	*
10	KLEQCNTELKK-KEAKPGR-a1-b1	SMC1_B	197	SMC3_B	968	*
11	KLEQCNTELKK-KEAKPGR-a10-b1	SMC1_B	197	SMC3_B	977	*
12	KEAKPGR-YSHVNKK-a1-b6	SMC1_B	197	SMC3_B	984	
13	KEAKPGR-EKLIKR-a1-b2	SMC1_B	197	SMC3_B	999	
	KEAKPGRK-EKLIK-a1-b2	SMC1_B	197	SMC3_B	999	
14	EAKPGR-VIGAKK-a3-b5	SMC1_B	200	SMC3_B	105	
15	LHTLEGTKK-EAKPGRK-a8-b3	SMC1_B	200	SMC3_B	492	*
	LHTLEGTKK-KEAKPGR-a8-b4	SMC1_B	200	SMC3_B	492	*
15	LHTLEGTKK-KEAKPGRK-a8-b4	SMC1_B	200	SMC3_B	492	*
16	EAKPGR-KQQLLR-a3-b1	SMC1_B	200	SMC3_B	493	
	KEAKPGR-KQQLLR-a4-b1	SMC1_B	200	SMC3_B	493	
17	AATGKAILNGIDSINK-EAKPGR-a5-b3	SMC1_B	200	SMC3_B	503	*
	AATGKAILNGIDSINK-KEAKPGR-a5-b4	SMC1_B	200	SMC3_B	503	*
18	KLEQCNTELK-KEAKPGR-a1-b4	SMC1_B	200	SMC3_B	968	*
	KLEQCNTELKK-KEAKPGR-a1-b4	SMC1_B	200	SMC3_B	968	*
19	KYSHVNKK-KEAKPGR-a1-b4	SMC1_B	200	SMC3_B	978	*
20	KEAKPGR-YSHVNKK-a4-b6	SMC1_B	200	SMC3_B	984	

21	ALDQFVNFSEQKEK-EAKPGR-a12-b3	SMC1_B	200	SMC3_B	997	*
	ALDQFVNFSEQKEK-EAKPGRK-a12-b3	SMC1_B	200	SMC3_B	997	*
22	KEAKPGR-EKLIK-a4-b2	SMC1_B	200	SMC3_B	999	
23	KAEIMESIKR-LHTLEGTKK-a1-b8	SMC1_B	500	SMC3_B	492	
24	KAEIMESIKR-KQQLLR-a1-b1	SMC1_B	500	SMC3_B	493	
25	VLGKNMDAIIVDSEK-KQQLLR-a4-b1	SMC1_B	540	SMC3_B	493	
26	AATGKAILNGIDSINK-VLGKNMDAIIVDSEK-a5-b4	SMC1_B	540	SMC3_B	503	*
27	VLGKNMDAIIVDSEK-HVFGKTLICR-a4-b5	SMC1_B	540	SMC3_B	629	
28	VLGKNMDAIIVDSEK-LELQKDVR-a4-b5	SMC1_B	540	SMC3_B	680	
29	DCIQYIKEQR-LHTLEGTKK-a7-b8	SMC1_B	561	SMC3_B	492	
	TGRDCIQYIKEQR-LHTLEGTKK-a10-b8	SMC1_B	561	SMC3_B	492	
30	DCIQYIKEQR-KQQLLR-a7-b1	SMC1_B	561	SMC3_B	493	
	TGRDCIQYIKEQR-KQQLLR-a10-b1	SMC1_B	561	SMC3_B	493	
31	DCIQYIKEQR-KSRLELQK-a7-b1	SMC1_B	561	SMC3_B	673	
	TGRDCIQYIKEQR-KSRLELQK-a10-b1	SMC1_B	561	SMC3_B	673	
32	DCIQYIKEQR-LELQKDVR-a7-b5	SMC1_B	561	SMC3_B	680	
	DCIQYIKEQR-LELQKDVRK-a7-b5	SMC1_B	561	SMC3_B	680	
32	DCIQYIKEQR-SRLELQKDVR-a7-b7	SMC1_B	561	SMC3_B	680	
	TGRDCIQYIKEQR-LELQKDVR-a10-b5	SMC1_B	561	SMC3_B	680	
32	TGRDCIQYIKEQR-LELQKDVRK-a10-b5	SMC1_B	561	SMC3_B	680	
33	TGRDCIQYIKEQR-YQTLSLKQLFR-a10-b7	SMC1_B	561	SMC3_B	963	
	YQTLSLKQLFR-DCIQYIKEQR-a7-b7	SMC1_B	561	SMC3_B	963	*
34	KLEQCNTELKK-DCIQYIKEQR-a1-b7	SMC1_B	561	SMC3_B	968	*
35	ALDQFVNFSEQKEK-DCIQYIKEQR-a12-b7	SMC1_B	561	SMC3_B	997	*
36	HKTVALDGTLFQK-HVFGKTLICR-a2-b5	SMC1_B	637	SMC3_B	629	
37	LHTLEGTKK-AVDKLK-a8-b4	SMC1_B	673	SMC3_B	492	*
38	AVDKLK-KQQLLR-a4-b1	SMC1_B	673	SMC3_B	493	
39	AVDKLK-EKLIK-a4-b2	SMC1_B	673	SMC3_B	999	
40	LLKLFTR-VKTLR-a3-b2	SMC1_B	59	SCC1	387	*
41	EKEKEDDEEEEDEDASGGDQDQEER-VKTLR-a4-b2	SMC1_B	59	SCC1	533	*
42	VIVGGSSEYKINNK-FFTQPDKNFSNTK-a10-b7	SMC1_B	106	PDS5B	1103	
43	LKSNDNEER-KEAKPGR-a2-b1	SMC1_B	197	PDS5B	282	*
44	KEAKPGR-TLDKR-a1-b4	SMC1_B	197	PDS5B	400	
45	KEAKPGR-TLDKR-a4-b4	SMC1_B	200	PDS5B	400	
46	WDEKAVDKLK-TLDKR-a8-b4	SMC1_B	673	PDS5B	400	
47	IWLAAHWDKK-KMVTK-a9-b1	SMC3_B	114	SCC1	25	*

48	AKYLLADCNEAFIK-ETEGKREK-a2-b5	SMC3_B	185	SCC1	72	*
	KAKYLLADCNEAFIK-ETEGKREK-a3-b5	SMC3_B	185	SCC1	72	*
49	KAKYLLADCNEAFIK-EKINELLK-a3-b2	SMC3_B	188	SCC1	72	*
50	IKMAFRPGVVDLPEENR-EKINELLK-a2-b2	SMC3_B	188	SCC1	86	*
51	KYEAIQLTFKQVSK-VKMALR-a10-b2	SMC3_B	1034	SCC1	50	
52	QVSKNFSEVFQK-VKMALR-a4-b2	SMC3_B	1038	SCC1	50	
53	VSFTGKQGEMR-LLKLFTR-a6-b3	SMC3_B	1105	SCC1	387	
54	INELLKYIEER-LELLLQKRK-a6-b7	SMC3_B	194	SA1	273	
55	SYRDQTIVDPFSSKHNVIVGR-EISDKISKEEMVR-a14-b5	SMC3_B	26	PDS5B	25	
56	QLKGLEDTK-KMVTK-a3-b1	SMC3_B	114	PDS5B	115	*
57	KKTPVTEQEEK-LKLLR-a2-b2	SMC3_B	157	PDS5B	1219	*
58	LHTLEGTKK-LKSNDNEER-a8-b2	SMC3_B	492	PDS5B	282	
	LKSNDNEERLQVVK-LHTLEGTKK-a2-b8	SMC3_B	492	PDS5B	282	*
59	LKSNDNEER-KQQLLR-a2-b1	SMC3_B	493	PDS5B	282	*
	LKSNDNEERLQVVK-KQQLLR-a2-b1	SMC3_B	493	PDS5B	282	*
60	DTAYPETNDAIPMISKLR-LVQEQKPKGSQR-a16-b6	SMC3_B	612	PDS5B	1242	
61	LKSNDNEER-LELQKDVR-a2-b5	SMC3_B	680	PDS5B	282	*
62	YQTLSLKQLFR-LKSNDNEER-a7-b2	SMC3_B	963	PDS5B	282	
63	YQTLSLKQLFR-DLTEYLKVR-a7-b7	SMC3_B	963	PDS5B	356	
64	KLEQCNTELKK-LKSNDNEER-a1-b2	SMC3_B	968	PDS5B	282	
65	KLEQCNTELKK-LKSNDNEER-a10-b2	SMC3_B	977	PDS5B	282	
66	LKSNDNEER-KYSHVNK-a2-b1	SMC3_B	978	PDS5B	282	*
	LKSNDNEER-KYSHVNKK-a2-b1	SMC3_B	978	PDS5B	282	*
66	LKSNDNEERLQVVK-KYSHVNK-a2-b1	SMC3_B	978	PDS5B	282	*
67	LKSNDNEER-KYSHVNKK-a2-b7	SMC3_B	984	PDS5B	282	*
	LKSNDNEER-YSHVNKK-a2-b6	SMC3_B	984	PDS5B	282	*
67	LKSNDNEERLQVVK-KYSHVNKK-a2-b7	SMC3_B	984	PDS5B	282	*
	LKSNDNEERLQVVK-YSHVNKK-a2-b6	SMC3_B	984	PDS5B	282	*
68	KALDQFVNFSEQK-LKSNDNEER-a1-b2	SMC3_B	985	PDS5B	282	
69	FYGVKFR-SELEKPR-a5-b5	SMC3_B	1190	PDS5B	1213	
70	FYGVKFR-SKQHR-a5-b2	SMC3_B	1190	PDS5B	1344	
71	NKVSHIDVITAEMAK-LVQEQKPKGSQR-a2-b6	SMC3_B	1194	PDS5B	1242	
72	GRPGRPPSTNKKPR-ETKAK-a11-b3	SCC1	317	SA1	48	*
73	KGRPPLHK-ETKAK-a1-b3	SCC1	317	SA1	1079	*
74	GRPGRPPSTNKKPR-KLIVDSVK-a11-b1	SCC1	323	SA1	48	*
75	KLIVDSVK-KSPGEKSR-a1-b6	SCC1	323	SA1	57	

76	KTVHSYLEK-KLIVDSVK-a1-b1	SCC1	323	SA1	1020	*
77	MSVNSGSSSSKTSSVR-KLIVDSVK-a11-b1	SCC1	323	SA1	1071	*
	NSLVTGGEDDRMSVNSGSSSSKTSSVR-KLIVDSVK-a22-b1	SCC1	323	SA1	1071	*
78	GRPPLHKK-KLIVDSVK-a7-b1	SCC1	323	SA1	1086	*
79	ELDSKTIR-GRPPLHKK-a5-b7	SCC1	335	SA1	1086	
80	LLKLFTR-MIGKR-a3-b4	SCC1	387	SA1	261	
81	LLKLFTR-GTGKR-a3-b4	SCC1	387	SA1	549	
82	ITDGSPSKEDLLVLR-KGGEADNLDEFLK-a8-b1	SCC1	406	SA1	759	*
	ITDGSPSKEDLLVLR-RKGGEADNLDEFLK-a8-b2	SCC1	406	SA1	759	*
	ITDGSPSKEDLLVLRK-KGGEADNLDEFLK-a8-b1	SCC1	406	SA1	759	*
	ITDGSPSKEDLLVLRK-RKGGEADNLDEFLK-a8-b2	SCC1	406	SA1	759	*
83	KGGEADNLDEFLK-QIDKIQCAK-a1-b4	SCC1	406	SA1	916	
84	FALTFGLDQIKTR-KGGEADNLDEFLK-a11-b1	SCC1	406	SA1	969	*
	RFALTFGLDQIKTR-KGGEADNLDEFLK-a12-b1	SCC1	406	SA1	969	*
85	IKMAFRPGVVDLPEENR-LKSNDNEER-a2-b2	SCC1	86	PDS5B	282	
86	GRPPKPLGGGTPK-ELDSKTIR-a5-b5	SCC1	335	PDS5B	1295	*
87	LLKLFTR-SKQAATK-a3-b2	SCC1	387	PDS5B	1397	
88	NNHSKSGTSTLR-EKEKEK-a5-b4	SCC1	527	PDS5B	845	*
89	GRPGRPPSTNKKPRK-QVFAQKLHKGLSR-a11-b6	SA1	48	PDS5B	925	
90	GRPGRPPSTNKKPR-LVQEQKPKGSQR-a11-b8	SA1	48	PDS5B	1244	
91	GRPGRPPSTNKKPR-SKQHR-a11-b2	SA1	48	PDS5B	1344	
92	GRPGRPPSTNKKPR-SKQAATK-a11-b2	SA1	48	PDS5B	1397	
93	GRPGRPPSTNKKPR-SKQAATK-a12-b2	SA1	49	PDS5B	1397	
94	NNHSKSGTSTLR-KSPGEK-a5-b1	SA1	52	PDS5B	845	*
95	KKTPVTEQEEK-KSPGEK-a2-b1	SA1	52	PDS5B	1219	*
96	KSPGEKSR-SKQAATK-a1-b2	SA1	52	PDS5B	1397	
	SKQAATK-KSPGEK-a2-b1	SA1	52	PDS5B	1397	*
97	KKTPVTEQEEK-KSPGEKSR-a2-b6	SA1	57	PDS5B	1219	*
98	KSPGEKSR-SKQAATK-a6-b2	SA1	57	PDS5B	1397	
99	HDPQAEEALAKR-TLDKR-a11-b4	SA1	453	PDS5B	400	
100	HDPQAEEALAKR-SKQAATK-a11-b2	SA1	453	PDS5B	1397	
101	ETLSKTRQIDK-SELEKPR-a5-b5	SA1	910	PDS5B	1213	
102	QIDKIQCAK-SELEKPRGR-a4-b5	SA1	916	PDS5B	1213	

Supplementary Table 2 All peptides containing intramolecular crosslinks that were identified in Pds5B-bound bonsai cohesin. An asterisk in the column “Swap” indicates that the crosslinked peptide fragments a and b belong to residue 2 and residue 1, respectively.

	Unique peptide	Protein	Res 1	Res 2	Swap
1	TLRDLIHGAPVGKPAANR-SYKGR-a13-b3	SMC1_B	16	72	*
	DLIHGAPVGKPAANR-SYKGR-a10-b3	SMC1_B	16	72	*
2	VIVGGSSEYKINNK-SYKGR-a10-b3	SMC1_B	16	106	*
3	SNLMDAISFVLGEKTSNLR-VKTLR-a14-b2	SMC1_B	52	59	
4	DLIHGAPVGKPAANR-VKTLR-a10-b2	SMC1_B	59	72	*
5	VIVGGSSEYKINNK-VKTLR-a10-b2	SMC1_B	59	106	*
6	DLIHGAPVGKPAANR-VIVGGSSEYKINNK-a10-b10	SMC1_B	72	106	
7	EMVKAEEDTQFNYHR-SGELAQEYDKR-a4-b10	SMC1_B	170	177	*
8	LIDLCQPTQKK-SGELAQEYDKR-a10-b10	SMC1_B	170	528	*
9	KEAKPGR-KKEMVK-a1-b1	SMC1_B	172	197	*
10	KEAKPGR-KKEMVK-a1-b2	SMC1_B	173	197	*
11	EMVKAEEDTQFNYHRK-KNIAAERK-a4-b1	SMC1_B	177	190	
12	AEEDTQFNYHRKK-KNIAAER-a12-b1	SMC1_B	189	190	
13	KKNIAAER-EAKPGR-a1-b3	SMC1_B	189	200	
14	KEAKPGR-KNIAAER-a1-b1	SMC1_B	190	197	*
	KKNIAAER-KEAKPGR-a2-b1	SMC1_B	190	197	
15	KNIAAER-EAKPGR-a1-b3	SMC1_B	190	200	
16	AEIMESIKR-KKNIAAER-a8-b2	SMC1_B	190	508	*
	AEIMESIKR-KNIAAER-a8-b1	SMC1_B	190	508	*
17	TGRDCIQYIKEQR-KNIAAERK-a10-b1	SMC1_B	190	561	*
	TGRDCIQYIKEQR-KNIAAER-a10-b1	SMC1_B	190	561	*
18	SGVISGGASDLKAK-KNIAAER-a12-b1	SMC1_B	190	660	*
19	KNIAAER-AVDKLK-a1-b4	SMC1_B	190	673	
20	IAAPNMKAMEK-KNIAAER-a7-b1	SMC1_B	190	1026	*
21	AMEKLESVRDK-KNIAAERK-a4-b1	SMC1_B	190	1030	*
22	DKFQETSDEFEAAR-KKNIAAER-a2-b2	SMC1_B	190	1037	*
23	AKQAFEQIKK-KNIAAERK-a2-b1	SMC1_B	190	1056	*
24	KAEIMESIK-KEAKPGR-a1-b1	SMC1_B	197	500	*

	KAEIMESIKR-KEAKPGR-a1-b1	SMC1_B	197	500	*
25	KAEIMESIKR-KEAKPGR-a9-b1	SMC1_B	197	508	*
26	LIDLCQPTQKK-KEAKPGR-a10-b1	SMC1_B	197	528	*
27	VLGKNMDAIIVDSEK-KEAKPGR-a4-b1	SMC1_B	197	540	*
28	TGRDCIQYIKEQR-KEAKPGR-a10-b1	SMC1_B	197	561	*
	DCIQYIKEQR-KEAKPGR-a7-b1	SMC1_B	197	561	*
	TGRDCIQYIKEQR-KEAKPGRK-a10-b1	SMC1_B	197	561	*
29	KEAKPGRK-AVDKLK-a1-b4	SMC1_B	197	673	
	KEAKPGR-AVDKLK-a1-b4	SMC1_B	197	673	
30	AMEKLESVR-KEAKPGR-a4-b1	SMC1_B	197	1030	*
31	LESVRDKFQETSDEFEAAR-KEAKPGR-a7-b1	SMC1_B	197	1037	*
	DKFQETSDEFEAAR-KEAKPGR-a2-b1	SMC1_B	197	1037	*
32	KAEIMESIK-KEAKPGR-a1-b4	SMC1_B	200	500	*
33	KAEIMESIKR-EAKPGR-a9-b3	SMC1_B	200	508	*
34	LIDLCQPTQKK-KEAKPGR-a10-b4	SMC1_B	200	528	*
35	DCIQYIKEQR-KEAKPGR-a7-b4	SMC1_B	200	561	*
	DCIQYIKEQR-EAKPGR-a7-b3	SMC1_B	200	561	*
36	SGVISGGASDLKAK-KEAKPGR-a12-b4	SMC1_B	200	660	*
37	AVDKLK-EAKPGR-a4-b3	SMC1_B	200	673	*
	KEAKPGR-AVDKLK-a4-b4	SMC1_B	200	673	
	KEAKPGRK-AVDKLK-a4-b4	SMC1_B	200	673	
38	LESVRDKFQETSDEFEAAR-KEAKPGR-a7-b4	SMC1_B	200	1037	*
	DKFQETSDEFEAAR-KEAKPGR-a2-b4	SMC1_B	200	1037	*
39	VLGKNMDAIIVDSEK-KAEIMESIK-a4-b1	SMC1_B	500	540	*
40	KAEIMESIKR-WDEKAVDK-a1-b4	SMC1_B	500	669	
	KAEIMESIKR-RWDEKAVDK-a1-b5	SMC1_B	500	669	
41	KAEIMESIK-AVDKLK-a1-b4	SMC1_B	500	673	
	KAEIMESIKR-AVDKLK-a1-b4	SMC1_B	500	673	
42	VLGKNMDAIIVDSEK-KAEIMESIKR-a4-b9	SMC1_B	508	540	*
43	KAEIMESIKR-WDEKAVDK-a9-b4	SMC1_B	508	669	
	KAEIMESIKR-RWDEKAVDK-a9-b5	SMC1_B	508	669	
44	AEIMESIKR-AVDKLK-a8-b4	SMC1_B	508	673	
	KAEIMESIKR-AVDKLK-a9-b4	SMC1_B	508	673	
45	SGVISGGASDLKAK-LIDLCQPTQKK-a12-b10	SMC1_B	528	660	*
46	LIDLCQPTQKK-WDEKAVDK-a10-b4	SMC1_B	528	669	
47	LIDLCQPTQKK-AVDKLK-a10-b4	SMC1_B	528	673	

48	KYQIAVTK-LRELKGAK-a1-b5	SMC1_B	529	589	
	KYQIAVTK-ELKGAK-a1-b3	SMC1_B	529	589	
49	GAKLVIDVIR-KYQIAVTK-a3-b1	SMC1_B	529	592	*
	GAKLVIDVIRYEPPHIK-KYQIAVTK-a3-b1	SMC1_B	529	592	*
50	SGVISGGASDLKAK-KYQIAVTK-a12-b1	SMC1_B	529	660	*
51	VLGKNMDAIIVDSEK-HKTVALDGTLFQK-a4-b2	SMC1_B	540	637	
52	GEPETFLPLDYLEVKPTDEK-HKTVALDGTLFQK-a15-b2	SMC1_B	579	637	
	EQRGEPETFLPLDYLEVKPTDEK-HKTVALDGTLFQK-a18-b2	SMC1_B	579	637	
53	EQRGEPETFLPLDYLEVKPTDEK-TVALDGTLFQKSGVISGGASDLK-a18-b11	SMC1_B	579	648	
	TVALDGTLFQKSGVISGGASDLK-GEPETFLPLDYLEVKPTDEK-a11-b15	SMC1_B	579	648	*
54	SGVISGGASDLKAK-AVDKLK-a12-b4	SMC1_B	660	673	
55	DKFQETSDEFEAARK-IAAPNMKAMEK-a2-b7	SMC1_B	1026	1037	*
	DKFQETSDEFEAAR-IAAPNMKAMEK-a2-b7	SMC1_B	1026	1037	*
	LESVRDKFQETSDEFEAAR-IAAPNMKAMEK-a7-b7	SMC1_B	1026	1037	*
56	DKFQETSDEFEAAR-AMEKLESVR-a2-b4	SMC1_B	1030	1037	*
57	AKQAFEQIK-KERFDR-a2-b1	SMC1_B	1056	1064	
58	DFVEDDTTHGDYKDDDDK-SNPYYIVKQGK-a13-b8	SMC3_B	140	1220	*
59	VYDERKEESISLMK-LKLLR-a6-b2	SMC3_B	157	172	*
60	KYEAIQLTFK-LKLLR-a1-b2	SMC3_B	157	1025	*
61	EKINELLK-ETEGKR-a2-b5	SMC3_B	185	188	*
62	AATGKAILNGIDSINK-LHTLEGTKK-a5-b8	SMC3_B	492	503	*
63	LHTLEGTKK-KSRLELQK-a8-b1	SMC3_B	492	673	
64	LHTLEGTKK-LELQKDVR-a8-b5	SMC3_B	492	680	
	SRLELQKDVR-LHTLEGTKK-a7-b8	SMC3_B	492	680	*
65	YQTLSLKQLFR-LHTLEGTKK-a7-b8	SMC3_B	492	963	*
66	KLEQCNTELKK-LHTLEGTKK-a1-b8	SMC3_B	492	968	*
	KLEQCNTELK-LHTLEGTKK-a1-b8	SMC3_B	492	968	*
67	KLEQCNTELKK-LHTLEGTKK-a10-b8	SMC3_B	492	977	*
68	LHTLEGTKK-KYSHVNK-a8-b1	SMC3_B	492	978	
	LHTLEGTKK-KYSHVNKK-a8-b1	SMC3_B	492	978	
69	LHTLEGTKK-KYSHVNKK-a8-b7	SMC3_B	492	984	
	LHTLEGTKK-YSHVNKK-a8-b6	SMC3_B	492	984	
70	KALDQFVNFSEQK-LHTLEGTKK-a1-b8	SMC3_B	492	985	*
71	ALDQFVNFSEQKEK-LHTLEGTKK-a12-b8	SMC3_B	492	997	*
72	AATGKAILNGIDSINK-KQQLLR-a5-b1	SMC3_B	493	503	*
73	LELQKDVR-KQQLLR-a5-b1	SMC3_B	493	680	*

	SRLELQKDVR-KQQLLR-a7-b1	SMC3_B	493	680	*
74	YQTLSLKQLFR-KQQLLR-a7-b1	SMC3_B	493	963	*
75	KLEQCNTELK-KQQLLR-a1-b1	SMC3_B	493	968	*
	KLEQCNTELKK-KQQLLR-a1-b1	SMC3_B	493	968	*
76	KLEQCNTELKK-KQQLLR-a10-b1	SMC3_B	493	977	*
77	KYSHVNK-KQQLLR-a1-b1	SMC3_B	493	978	*
	KYSHVNKK-KQQLLR-a1-b1	SMC3_B	493	978	*
78	YSHVNKK-KQQLLR-a6-b1	SMC3_B	493	984	*
	KYSHVNKK-KQQLLR-a7-b1	SMC3_B	493	984	*
79	KALDQFVNFSEQK-KQQLLR-a1-b1	SMC3_B	493	985	*
80	ALDQFVNFSEQKEK-KQQLLR-a12-b1	SMC3_B	493	997	*
81	AATGKAILNGIDSINK-YQTLSLKQLFR-a5-b7	SMC3_B	503	963	
82	AATGKAILNGIDSINK-KLEQCNTELK-a5-b1	SMC3_B	503	968	
83	AATGKAILNGIDSINK-KLEQCNTELKK-a5-b10	SMC3_B	503	977	
84	AATGKAILNGIDSINK-YSHVNKK-a5-b6	SMC3_B	503	984	
85	MNLPGEVTFLPLNKLDVR-FDKAFK-a14-b3	SMC3_B	592	621	
86	HVFGKTLICR-FDKAFK-a5-b3	SMC3_B	621	629	*
87	HVFGKTLICR-KSRLELQK-a5-b1	SMC3_B	629	673	
88	HVFGKTLICR-LELQKDVR-a5-b5	SMC3_B	629	680	
89	YQTLSLKQLFR-LELQKDVR-a7-b5	SMC3_B	680	963	*
	YQTLSLKQLFR-SRLELQKDVR-a7-b7	SMC3_B	680	963	*
90	KLEQCNTELKK-SRLELQKDVR-a1-b7	SMC3_B	680	968	*
	KLEQCNTELKK-LELQKDVR-a1-b5	SMC3_B	680	968	*
	KLEQCNTELK-LELQKDVR-a1-b5	SMC3_B	680	968	*
91	KYSHVNKK-LELQKDVR-a1-b5	SMC3_B	680	978	*
92	SRLELQKDVR-YSHVNKK-a7-b6	SMC3_B	680	984	
93	KLEQCNTELKK-YQTLSLKQLFR-a1-b7	SMC3_B	963	968	*
	YQTLSLKQLFR-KLEQCNTELK-a7-b1	SMC3_B	963	968	
94	KLEQCNTELKK-YQTLSLKQLFR-a10-b7	SMC3_B	963	977	*
95	KALDQFVNFSEQK-YQTLSLKQLFR-a1-b7	SMC3_B	963	985	*
96	ALDQFVNFSEQKEK-YQTLSLKQLFR-a12-b7	SMC3_B	963	997	*
97	YQTLSLKQLFR-EKLIK-a7-b2	SMC3_B	963	999	
98	KLEQCNTELK-KYSHVNK-a1-b1	SMC3_B	968	978	
	KLEQCNTELK-KYSHVNKK-a1-b1	SMC3_B	968	978	
99	KLEQCNTELKK-YSHVNKK-a1-b6	SMC3_B	968	984	
100	ALDQFVNFSEQKEK-KLEQCNTELK-a12-b1	SMC3_B	968	997	*

	ALDQFVNFSEQKEK-KLEQCNTELKK-a12-b1	SMC3_B	968	997	*
101	KLEQCNTELKK-YSHVNKK-a10-b6	SMC3_B	977	984	
102	KALDQFVNFSEQK-KLEQCNTELKK-a1-b10	SMC3_B	977	985	*
103	KALDQFVNFSEQK-KYSHVNK-a1-b1	SMC3_B	978	985	*
104	KALDQFVNFSEQK-YSHVNKK-a1-b6	SMC3_B	984	985	*
105	KGDVEGSQSQDEGEGSGESER-KYEAIQLTFKQVSK-a1-b1	SMC3_B	1025	1059	*
106	KYEAIQLTFKQVSK-LVPGGKATLVMK-a10-b6	SMC3_B	1034	1052	
107	KGDVEGSQSQDEGEGSGESER-KYEAIQLTFKQVSK-a1-b10	SMC3_B	1034	1059	*
108	LVPGGKATLVMK-QVSKNFSEVFQK-a6-b4	SMC3_B	1038	1052	*
109	LVPGGKATLVMK-VSFTGKQGEMR-a6-b6	SMC3_B	1052	1105	
110	DFVEDDTTHGDYKDDDDK-VSFTGKQGEMR-a13-b6	SMC3_B	1105	1220	*
111	NKVSHIDVITAEMAK-FYGVKFR-a2-b5	SMC3_B	1190	1194	*
112	DFVEDDTTHGDYKDDDDK-FYGVKFR-a13-b5	SMC3_B	1190	1220	*
113	DFVEDDTTHGDYKDDDDK-NKVSHIDVITAEMAK-a13-b2	SMC3_B	1194	1220	*
114	KLIVDSVK-ETKAK-a1-b3	SCC1	317	323	*
	KLIVDSVK-ETKAKR-a1-b3	SCC1	317	323	*
	RKLIVDSVK-ETKAK-a2-b3	SCC1	317	323	*
115	ELDSKTIR-KLIVDSVK-a5-b1	SCC1	323	335	*
116	ETGGVEKLFSLPAQPLWNNR-LLKLFTR-a7-b3	SCC1	371	387	
117	KGGEADNLDEFLK-LLKLFTR-a1-b3	SCC1	387	406	*
118	EKEDDEEEEDEDASGGDQDQEERR-EKEKEK-a2-b4	SCC1	527	533	*
119	ALAKTGAESISLLELCR-KQAAAKFYSFLVLK-a4-b1	SCC1	573	591	
120	ALAKTGAESISLLELCR-QAAAKFYSFLVLK-a4-b5	SCC1	573	596	
	ALAKTGAESISLLELCR-KQAAAKFYSFLVLK-a4-b6	SCC1	573	596	
	ALAKTGAESISLLELCR-QAAAKFYSFLVLKK-a4-b5	SCC1	573	596	
121	GRPGRPPSTNKKPR-KSPGEK-a11-b1	SA1	48	52	
	RGRPGRPPSTNKKPR-KSPGEK-a12-b1	SA1	48	52	
122	GRPGRPPSTNKKPR-KSPGEKSR-a11-b6	SA1	48	57	
	RGRPGRPPSTNKKPR-KSPGEKSR-a12-b6	SA1	48	57	
	GRPGRPPSTNKKPR-SPGEKSR-a11-b5	SA1	48	57	
123	RGRPGRPPSTNKKPR-LELLLQKR-a12-b7	SA1	48	273	
	GRPGRPPSTNKKPR-ANERLELLLQKR-a11-b11	SA1	48	273	
	GRPGRPPSTNKKPR-LELLLQKR-a11-b7	SA1	48	273	
124	GRPGRPPSTNKKPR-VLTAKER-a11-b5	SA1	48	555	
125	GRPGRPPSTNKKPR-KTQIDDR-a11-b1	SA1	48	558	
126	GRPGRPPSTNKKPR-KTVHSYLEK-a11-b1	SA1	48	1020	

127	GRPGRPPSTNKKPR-KSPGEK-a12-b1	SA1	49	52	
	RGRPGRPPSTNKKPR-KSPGEK-a13-b1	SA1	49	52	
128	GRPGRPPSTNKKPR-KSPGEKSR-a12-b6	SA1	49	57	
129	RGRPGRPPSTNKKPR-LELLLQKR-a13-b7	SA1	49	273	
130	GRPGRPPSTNKKPR-KTVHSYLEK-a12-b1	SA1	49	1020	
131	LELLLQKR-KSPGEK-a7-b1	SA1	52	273	*
	ANERLELLLQKR-KSPGEK-a11-b1	SA1	52	273	*
132	KSPGEKSR-LELLLQKR-a6-b7	SA1	57	273	
133	GRPPLHKK-KSPGEKSR-a7-b6	SA1	57	1086	*
134	HDPQAEEALAKR-MIGKR-a11-b4	SA1	261	453	*
135	TGMNYMKVR-LELLLQKR-a7-b7	SA1	273	1177	*
136	HDPQAEEALAKR-VLTAKER-a11-b5	SA1	453	555	
137	HDPQAEEALAKR-KTQIDDR-a11-b1	SA1	453	558	
138	LEDLNRKDR-GTGKR-a7-b4	SA1	549	1168	*
139	KTQIDDR-VLTAKER-a1-b5	SA1	555	558	*
	KTQIDDRNK-VLTAKER-a1-b5	SA1	555	558	*
	KTQIDDRNK-RVLTAKER-a1-b6	SA1	555	558	*
	RVLTAKER-KTQIDDR-a6-b1	SA1	555	558	
140	FVVEKHVESDVLEACSK-HLDALLKQIK-a5-b7	SA1	618	626	*
141	YYNDYGDIIKETLSK-QIDKIQCAK-a10-b4	SA1	905	916	
142	YYNDYGDIIKETLSK-RFALTFGLDQIKTR-a10-b12	SA1	905	969	
143	QIDKIQCAK-ETLSKTR-a4-b5	SA1	910	916	*
144	FALTFGLDQIKTR-QIDKIQCAK-a11-b4	SA1	916	969	*
	RFALTFGLDQIKTR-QIDKIQCAK-a12-b4	SA1	916	969	*
	RFALTFGLDQIKTR-TRQIDKIQCAK-a12-b6	SA1	916	969	*
	FALTFGLDQIKTR-TRQIDKIQCAK-a11-b6	SA1	916	969	*
145	EAVATLHKDGIEFAFK-QIDKIQCAK-a8-b4	SA1	916	979	*
146	TVHSYLEKFLTEQMMER-EAVATLHKDGIEFAFK-a8-b8	SA1	979	1028	*
147	GRPPLHKK-LLRQDKK-a7-b6	SA1	1019	1086	*
148	MSVNSGSSSSKTSSVR-KTVHSYLEK-a11-b1	SA1	1020	1071	*
149	KGRPPLHKK-KTVHSYLEK-a1-b1	SA1	1020	1079	*
150	KTVHSYLEK-GRPPLHKK-a1-b7	SA1	1020	1086	
151	KRVEDESLDNTWLNR-KGRPPLHK-a1-b1	SA1	1079	1087	*
152	LEDLNRKDR-TGMNYMKVR-a7-b7	SA1	1168	1177	
153	EISDKISKEEMVR-TNDGKITYPPGVK-a5-b5	PDS5B	12	25	*
154	IYAPEAPYTSPDKLK-TNDGKITYPPGVK-a13-b5	PDS5B	12	102	*

155	ISKEEMVRR-LKMVVK-a3-b2	PDS5B	28	36	
	EISDKISKEEMVR-RLKMVVK-a8-b3	PDS5B	28	36	
	ISKEEMVR-LKMVVK-a3-b2	PDS5B	28	36	
156	HPDKDVR-LKMVVK-a4-b2	PDS5B	36	74	*
	HPDKDVR-RLKMVVK-a4-b3	PDS5B	36	74	*
157	QLKGLEDTK-HPDKDVR-a3-b4	PDS5B	74	115	*
158	GLEDTKSPQFNR-HPDKDVR-a6-b4	PDS5B	74	121	*
159	GRPPKPLGGGTPK-GLEDTKSPQFNR-a5-b6	PDS5B	121	1295	*
160	MFGAKDSELASQNKPLWQCYLGR-KDILLVNDHLLNFVR-a5-b1	PDS5B	303	380	
161	FASHCLMNHPDLAKDLTEYLK-DSELASQNKPLWQCYLGR-a14-b9	PDS5B	312	349	*
162	SHDPEEAIRHDVIVSIVTAAKK-EAMMGLAQIYKK-a21-b11	PDS5B	379	417	
163	KDILLVNDHLLNFVR-KYALQSAAGK-a1-b1	PDS5B	380	418	
164	TTNVLGAVNKPLSSAGKQSQTK-TLDKR-a10-b4	PDS5B	400	1136	*
	TTNVLGAVNKPLSSAGK-TLDKR-a10-b4	PDS5B	400	1136	*
165	SELEKPR-TLDKR-a5-b4	PDS5B	400	1213	*
166	LVQEQKPKGSQR-TLDKR-a6-b4	PDS5B	400	1242	*
167	LVQEQKPKGSQR-TLDKR-a8-b4	PDS5B	400	1244	*
168	VAEAALQIFKNTGSK-ALNEMWKCQNLLR-a10-b7	PDS5B	497	693	*
169	VAEAALQIFKNTGSKIEEDFPHIR-ALNEMWKCQNLLR-a15-b7	PDS5B	497	698	*
170	KQLEVLVSPTCSCK-HQVKDLLDLIK-a1-b4	PDS5B	507	561	*
171	NTGSKIEEDFPHIR-HQVKDLLDLIK-a5-b4	PDS5B	507	698	*
172	QPKTDASVK-AQDFMKK-a3-b6	PDS5B	517	547	
173	TDASVKAIFSK-AQDFMKK-a6-b6	PDS5B	523	547	
174	NLPDPGKAQDFMK-KFTQVLEDDEK-a7-b1	PDS5B	541	548	
175	EYLKQHAAVSEK-AQDFMKK-a4-b6	PDS5B	547	974	*
	REYLKQHAAVSEK-AQDFMKK-a5-b6	PDS5B	547	974	*
176	KFTQVLEDDEK-KLGNPK-a1-b1	PDS5B	548	586	
177	LGNPKQPTNPFLEMIK-KFTQVLEDDEK-a5-b1	PDS5B	548	591	*
	KLGNPKQPTNPFLEMIK-KFTQVLEDDEK-a6-b1	PDS5B	548	591	*
178	KFTQVLEDDEKIR-EITKK-a11-b4	PDS5B	558	585	
	FTQVLEDDEKIR-EITKK-a10-b4	PDS5B	558	585	
179	FTQVLEDDEKIR-KLGNPK-a10-b1	PDS5B	558	586	
	FTQVLEDDEKIRK-KLGNPK-a10-b1	PDS5B	558	586	
180	LGNPKQPTNPFLEMIK-EITKK-a5-b4	PDS5B	585	591	*
181	QPTNPFLEMIKFLLER-EITKK-a11-b4	PDS5B	585	602	*
182	EITKKLGNPK-DPVKER-a4-b4	PDS5B	585	952	

183	VLSFTHPISFHSAETFESLLACLKMDDEK-KGPPR-a24-b1	PDS5B	678	720	
184	YAIHCIHAIFSSKETQFAQIFEPLHK-VAEAALQIFKNTGSK-a13-b10	PDS5B	693	740	*
185	NNHSKSGTSTLR-KKTPVTEQEEK-a5-b2	PDS5B	845	1219	
186	LVQEQKPKGSQR-NNHSKSGTSTLR-a8-b5	PDS5B	845	1244	*
187	NNHSKSGTSTLR-SKQHR-a5-b2	PDS5B	845	1344	
188	QCLVKNINVR-LHKGLSR-a5-b3	PDS5B	928	964	*
189	SFFTPGKPK-DPVKER-a7-b4	PDS5B	952	1124	*
190	TTNVLGAVNKPLSSAGK-DPVKER-a10-b4	PDS5B	952	1136	*
191	QSQTKSSR-DPVKERR-a5-b4	PDS5B	952	1148	*
192	REYLKQHAAVSEK-QCLVKNINVR-a5-b5	PDS5B	964	974	*
193	QCLVKNINVR-QSQTKSSR-a5-b5	PDS5B	964	1148	
194	STTYSLESPKDPVLPAR-REYLKQHAAVSEK-a10-b5	PDS5B	974	1089	*
	STTYSLESPKDPVLPAR-EYLKQHAAVSEK-a10-b4	PDS5B	974	1089	*
195	ECLWFVLEILMAKNENNSHAFIR-STTYSLESPKDPVLPAR-a13-b10	PDS5B	1031	1089	
196	FFTQPDKNFSNTK-KMVENIK-a7-b1	PDS5B	1042	1103	*
197	QSQTKSSR-KMVENIK-a5-b1	PDS5B	1042	1148	*
198	FFTQPDKNFSNTK-QTKDAQGPDDAK-a7-b3	PDS5B	1051	1103	*
199	NFSNTKNYLPPEMK-QTKDAQGPDDAK-a6-b3	PDS5B	1051	1109	*
200	NFSNTKNYLPPEMK-DAQGPDDAKMNEK-a6-b9	PDS5B	1060	1109	*
201	DAQGPDDAKMNEK-SKQHR-a9-b2	PDS5B	1060	1344	
202	STTYSLESPKDPVLPAR-QSQTKSSR-a10-b5	PDS5B	1089	1148	
203	FFTQPDKNFSNTK-QSQTKSSR-a7-b5	PDS5B	1103	1148	
204	TTNVLGAVNKPLSSAGK-SFFTPGKPK-a10-b7	PDS5B	1124	1136	*
205	SFFTPGKPK-QSQTKSSR-a7-b5	PDS5B	1124	1148	
206	TTNVLGAVNKPLSSAGK-QSQTKSSR-a10-b5	PDS5B	1136	1148	
207	KKTPVTEQEEK-QSQTKSSR-a2-b5	PDS5B	1148	1219	*
208	GRPPKPLGGGTPK-QSQTKSSR-a5-b5	PDS5B	1148	1295	*
209	KTPVTEQEEK-KSDKR-a1-b1	PDS5B	1197	1219	*
	KKTPVTEQEEK-KSDKR-a2-b1	PDS5B	1197	1219	*
210	LVQEQKPKGSQR-KSDKR-a8-b1	PDS5B	1197	1244	*
211	SDKRDDSDLVR-SELEKPR-a3-b5	PDS5B	1200	1213	
	SELEKPR-KSDKR-a5-b4	PDS5B	1200	1213	*
	DDSDLVRSELEKPR-KSDKR-a12-b4	PDS5B	1200	1213	*
212	KKTPVTEQEEK-SDKRDDSDLVR-a2-b3	PDS5B	1200	1219	*
	KKTPVTEQEEK-KSDKR-a2-b4	PDS5B	1200	1219	*
	SDKRDDSDLVR-KTPVTEQEEK-a3-b1	PDS5B	1200	1219	

213	KKTPVTEQEEK-SELEKPR-a1-b5	PDS5B	1213	1218	*
214	KKTPVTEQEEK-SELEKPR-a2-b5	PDS5B	1213	1219	*
	KTPVTEQEEK-SELEKPR-a1-b5	PDS5B	1213	1219	*
	DDSDLVRSELEKPR-KTPVTEQEEK-a12-b1	PDS5B	1213	1219	
	RDDSDLVRSELEKPR-KKTPVTEQEEK-a13-b2	PDS5B	1213	1219	
	DDSDLVRSELEKPR-KKTPVTEQEEK-a12-b2	PDS5B	1213	1219	
	RDDSDLVRSELEKPR-KTPVTEQEEK-a13-b1	PDS5B	1213	1219	
215	LVQEQKPKGSQR-SELEKPR-a6-b5	PDS5B	1213	1242	*
216	LVQEQKPKGSQR-SELEKPR-a8-b5	PDS5B	1213	1244	*
217	GRPPKPLGGGTPK-SELEKPR-a5-b5	PDS5B	1213	1295	*
218	KKTPVTEQEEK-LVQEQKPK-a1-b6	PDS5B	1218	1242	
	LVQEQKPKGSQR-KKTPVTEQEEK-a6-b1	PDS5B	1218	1242	*
219	LVQEQKPKGSQR-KKTPVTEQEEK-a8-b1	PDS5B	1218	1244	*
220	LGMDDLTKLVQEQKPK-KKTPVTEQEEK-a8-b2	PDS5B	1219	1236	*
221	KKTPVTEQEEK-LVQEQKPK-a2-b6	PDS5B	1219	1242	
	LVQEQKPKGSQR-KKTPVTEQEEK-a6-b2	PDS5B	1219	1242	*
	KTPVTEQEEK-LVQEQKPK-a1-b6	PDS5B	1219	1242	
222	LVQEQKPKGSQR-KKTPVTEQEEK-a8-b2	PDS5B	1219	1244	*
223	KKTPVTEQEEK-SKQHR-a2-b2	PDS5B	1219	1344	
224	KKTPVTEQEEK-SKQAATK-a2-b2	PDS5B	1219	1397	
225	TPVTEQEEKLGMDDLTK-LVQEQKPK-a9-b6	PDS5B	1228	1242	
	TPVTEQEEKLGMDDLTK-LVQEQKPKGSQR-a9-b6	PDS5B	1228	1242	
226	TPVTEQEEKLGMDDLTK-LVQEQKPKGSQR-a9-b8	PDS5B	1228	1244	
227	KRGHTASESDEQQWPEEK-LVQEQKPK-a1-b6	PDS5B	1242	1251	*
228	GRPPKPLGGGTPK-LVQEQKPK-a5-b6	PDS5B	1242	1295	*
229	LVQEQKPK-SKQAATK-a6-b2	PDS5B	1242	1397	
230	RGRPPKPLGGGTPK-LVQEQKPKGSQR-a6-b8	PDS5B	1244	1295	*
	GRPPKPLGGGTPK-LVQEQKPKGSQR-a5-b8	PDS5B	1244	1295	*
231	GRPPKPLGGGTPKEEPTMK-LVQEQKPKGSQR-a13-b8	PDS5B	1244	1303	*
232	LVQEQKPKGSQR-SKQHR-a8-b2	PDS5B	1244	1344	
233	LVQEQKPKGSQR-SKQAATK-a8-b2	PDS5B	1244	1397	
234	GRPPKPLGGGTPK-EEPTMKTSK-a5-b6	PDS5B	1295	1309	
	RGRPPKPLGGGTPK-EEPTMKTSK-a6-b6	PDS5B	1295	1309	
235	SGPPAPEEEEEEEERQSGNTEQKSK-GRPPKPLGGGTPK-a22-b5	PDS5B	1295	1340	*
236	GRPPKPLGGGTPK-SKQHR-a5-b2	PDS5B	1295	1344	
237	GRPPKPLGGGTPK-SKQAATK-a5-b2	PDS5B	1295	1397	

238	GRPPKPLGGGTPKEEPTMK-SKQHR-a13-b2	PDS5B	1303	1344	
239	GRPPKPLGGGTPKEEPTMKTSK-SKQHR-a19-b2	PDS5B	1309	1344	
240	KSGPPAPEEEEEEER-GSKKK-a1-b3	PDS5B	1316	1318	*
	KSGPPAPEEEEEEERQSGNTEQK-GSKKK-a1-b3	PDS5B	1316	1318	*
241	KSGPPAPEEEEEEERQSGNTEQK-SKQAATK-a1-b2	PDS5B	1318	1397	
242	SGPPAPEEEEEEERQSGNTEQKSK-SKQHR-a22-b2	PDS5B	1340	1344	
	QSGNTEQKSK-SKQHR-a8-b2	PDS5B	1340	1344	
243	SGPPAPEEEEEEERQSGNTEQKSK-SKQAATK-a22-b2	PDS5B	1340	1397	
244	GRPSKTPSPSQPK-SKQAATK-a5-b2	PDS5B	1380	1397	

Supplementary Table 3. Smc1 homologs used for sequence analysis

NCBI Accession	Organism
NP_006297.2	Homo sapiens
XP_001362224.1	Monodelphis domestica
XP_003216957.1	Anolis carolinensis
XP_002935560.1	Xenopus (Silurana) tropicalis
NP_989847.1	Gallus gallus
XP_004070486.1	Oryzias latipes
XP_003448214.1	Oreochromis niloticus
AAC15582.1	Takifugu rubripes
NP_001155103.1	Danio rerio
XP_786064.2	Strongylocentrotus purpuratus
XP_002735243.1	Saccoglossus kowalevskii
XP_009053511.1	Lottia gigantea
ELT90865.1	Capitella teleta
XP_005104984.1	Aplysia californica
XP_009027794.1	Helobdella robusta
XP_003748034.1	Metaseiulus occidentalis
NP_651211.2	Drosophila melanogaster
XP_001862294.1	Culex quinquefasciatus
XP_002430265.1	Pediculus humanus corporis
XP_001948129.1	Acyrthosiphon pisum
XP_006567381.1	Apis mellifera
XP_004923679.1	Bombyx mori
EFX81640.1	Daphnia pulex
CCD79777.1	Schistosoma mansoni
CDS34006.1	Hymenolepis microstoma
CDW56074.1	Trichuris trichiura

T34063	Caenorhabditis elegans
XP_001894059.1	Brugia malayi
CDJ96909.1	Haemonchus contortus
EYC11487.1	Ancylostoma ceylanicum
CBY22577.1	Oikopleura dioica
Q6Q1P4.2	Arabidopsis thaliana
XP_001767264.1	Physcomitrella patens
XP_005645407.1	Coccomyxa subellipsoidea C-169
XP_003055298.1	Micromonas pusilla CCMP1545
XP_001416713.1	Ostreococcus lucimarinus CCE9901
XP_007511160.1	Bathycoccus prasinos
XP_004340652.1	Acanthamoeba castellanii str. Neff
XP_002682487.1	Naegleria gruberi
ETO86191.1	Phytophthora parasitica P1976
CCI44657.1	Albugo candida
ETV87594.1	Aphanomyces astaci
CBN77803.1	Ectocarpus siliculosus
EFW46900.2	Capsaspora owczarzaki ATCC 30864
XP_002177808.1	Phaeodactylum tricornutum CCAP 1055/1
XP_004991317.1	Salpingoeca rosetta
NP_116647.1	Saccharomyces cerevisiae S288c
NP_596049.2	Schizosaccharomyces pombe 972h-
XP_005820384.1	Guillardia theta CCMP2712
XP_629977.1	Dictyostelium discoideum AX4
XP_656581.1	Entamoeba histolytica HM-1:IMSS
CBK21095.2	Blastocystis hominis
ETO34086.1	Reticulomyxa filosa

Supplementary Table 4 Smc3 homologs used for sequence analysis

NCBI Accession	Organism
NP_005436.1	Homo sapiens
NP_989848.1	Gallus gallus
O93309.2	Xenopus laevis
XP_004066378.1	Oryzias latipes
NP_001027798.1	Takifugu rubripes
NP_999854.1	Danio rerio
XP_007901837.1	Callorhinchus milii
XP_798572.3	Strongylocentrotus purpuratus
XP_005098268.1	Aplysia californica
XP_003739619.1	Metaseiulus occidentalis
AAC47078.1	Drosophila melanogaster
XP_004921724.1	Bombyx mori
XP_966409.1	Tribolium castaneum
XP_393700.2	Apis mellifera
EFN80015.1	Harpegnathos saltator
EFN66756.1	Camponotus floridanus
XP_002431125.1	Pediculus humanus corporis
EFX68226.1	Daphnia pulex
XP_002125440.1	Ciona intestinalis
XP_003389383.1	Amphimedon queenslandica
NP_001255118.1	Caenorhabditis elegans
XP_001899737.1	Brugia malayi
ERG82063.1	Ascaris suum
NP_001077968.1	Arabidopsis thaliana
XP_001784554.1	Physcomitrella patens
XP_003056807.1	Micromonas pusilla CCMP1545

XP_001419553.1	Ostreococcus lucimarinus CCE9901
CCA17167.1	Albugo laibachii Nc14
XP_002997684.1	Phytophthora infestans T30-4
CBN79764.1	Ectocarpus siliculosus
XP_002184568.1	Phaeodactylum tricornutum CCAP 1055/1
NP_593260.1	Schizosaccharomyces pombe 972h-
GAK66273.1	Pseudozyma antarctica
XP_005703178.1	Galdieria sulphuraria
XP_005712974.1	Chondrus crispus
XP_005536696.1	Cyanidioschyzon merolae strain 10D
NP_012461.1	Saccharomyces cerevisiae S288c
XP_655216.2	Entamoeba histolytica HM-1:IMSS
XP_643274.1	Dictyostelium discoideum AX4
XP_001328416.1	Trichomonas vaginalis G3
CBK23979.2	Blastocystis hominis
XP_557814.2	Anopheles gambiae str. PEST

Supplementary Methods

Sequence analysis

We predicted which segments of the antiparallel coiled coils in Smc1 and in Smc3 interact based on chemical crosslinking data, coiled coil prediction, structural information, and sequence conservation. Starting from human Smc1 (Smc1A; NCBI protein accession NP_006297.2) and Smc3 (NP_005436.1), we collected homologous sequences from a wide taxonomic range (see **Supplementary Table 3** and **Supplementary Table 4**), within the NCBI non redundant protein database using NCBI blastp[4] and with highly significant values below 1e-50. Sequences were aligned with MAFFT (L-INS-i method[5]) and the per residue conservation score was calculated using Jalview[6]. Coiled coil regions were predicted with ncoils, paircoils2 and marcoils [7-9] using default parameters (ncoils and paircoils: windows size 21). To combine all three coiled coil prediction algorithms, we applied a scoring system in which we assigned for each residue two points for a high significance (ncoils P-value >= 0.9, marcoils P-value >=0.9, paircoils score >= 10) and one point for low significance (ncoils P-value >= 0.8, marcoils P-value >=0.8, paircoils score >= 8). Two additional points were granted for an identical register position in all three programs, resulting in a maximum score of 8. For defining the terminal anchor points of the coiled coils, we considered X-ray crystallographic data deposited in the PDB database: 4UX3[10] (the budding yeast Smc3 head domain with long coiled coil regions in complex with $Scc1^N$), 2WD5[1] (the mouse Smc1/Smc3 heterodimeric hinge region), and 1W1W[11] (the budding yeast Smc1 head domain in complex with $Scc1^C$). The corresponding human Smc1 and Smc3 residues were mapped in a multiple alignment. The predicted coiled coil register and the high-resolution structural information were used to align the antiparallel coiled coils. Predicted interruptions were manually

positioned and proximity information from intramolecular crosslinks was included to validate and refine the alignment. Adobe Illustrator was used to generate a schematic illustration of our analysis. Phosphorylation sites that were experimentally observed in a number of studies without the use of phospho-specific antibodies were extracted from PhosphoSitePlus[12]. Missense and small in-frame deletions that were identified in Smc1 (*n*=27) and Smc3 (*n*=13) in Cornelia de Lange syndrome patients were collected from published data [13-17].

Differential Scanning Fluorimetry / ProteoPlex

Bonsai cohesin that included a 2xRFP tag (also described as tdimer[18]) on $Scc1^{C}$ was purified as described above (buffer B) and then applied to size-exclusion chromatography on a Superose 6 10/300 column to remove the excess of FLAG peptide. Cohesin was concentrated to 0.2-0.5 mg ml^{-1} and diluted in the screening conditions to a concentration of 0.1 mg ml^{-1}. Various buffer from the 96-well pH Screen (Jena Biosciences) were diluted 20x to final concentrations 50 mM (25 mM in case of CHES and CAPS) in the presence of 2mM $MgCl_2$ and 183 µM ATPγS (Jena Biosciences). Sypro orange (5000x stock in DMSO, Life Technologies) was used at a 5x concentration. Samples (20 µL) were premixed at 4°C in white 0.2 mL 96-well plates (Thermo Scientific), sealed with a clear 96-well microseal cover (Bio-Rad), centrifuged to remove possible air bubbles, and placed in a CFX96 real-time PCR machine (Bio-Rad). After a 5 minute incubation at 20°C, the temperature was increased by 2°C min^{-1} to 95°C. The fluorescent signal was measured in the FRET channel and analyzed as described[19,20]. All conditions were present in duplicate or in triplicate in a single experiment and average values were used for further analysis.

ATPase assays

ATPase assays were performed as described[21]. In brief, bonsai cohesin complexes were incubated in buffer B + 0.1 mg ml^{-1} BSA, 1 mM $MgCl_2$, 10 nM γ-[^{32}P] ATP and 50 μM non-radiolabelled ATP. Reactions were incubated at 37°C and stopped by adding 1% SDS and 10 mM EDTA. Reaction products were separated on polyethyleneimide plates (EMD Biosciences) by thin-layer-chromatography using 0.75 M KH_2PO_4 (pH 3.4) and analyzed by phosphor imaging with a Typhoon Trio Scanner (Amersham).

Crosslinking and mass spectrometry

Samples were prepared as above. FLAG peptide eluates were then immobilized on Ni-NTA beads and crosslinked. For the final dataset (see **Supplementary Fig. 13c**), an estimated amount of 500 μg of bonsai cohesin was bound to 250 μL Ni-NTA beads. The beads were then washed 5x with 4 bead volumes of buffer B + 183 μM ATPγS, 0.05% v/v Tween-20, and 35 mM imidazole and 3x with 4 bead volumes buffer B + 183 μM ATPγS. The beads were resuspended in 420 μL buffer B + 183 μM ATPγS. After a sample (20 μL) was taken for analysis by SDS-PAGE, the sample was divided and crosslinked at 23 °C with 0.8 and 2.4 mM of isotopically labeled disuccinimidyl suberate (DSS d_0-d_{12} (Creative Molecules Inc.), corresponding to an estimated 400 and 1200 fold-molar excess over cohesin (Pds5B-bound to bonsai cohesin tetramers contains 389 primary amines). The reaction was quenched after 20 minutes by adding ammonium bicarbonate at a final concentration of 75 mM. An aliquot of the reaction mixture (20 μL) was analyzed by SDS-PAGE after denaturing. Remaining material was digested on the Ni-NTA beads by Lys-C in 6 M urea followed by trypsin. Crosslinked peptides were enriched by size exclusion chromatography and analyzed by LC-MS/MS (liquid chromatography coupled to tandem mass spectrometry)[3,22]. Crosslinked

peptides were identified using xQuest software[23] and manually validated to a false discovery rate of ≤ 0.05. Representative ms/ms spectra of both high-scoring and low-scoring crosslinks are shown in **Supplementary Fig. 14.** Comprehensive circular diagrams of intermolecular and intramolecular crosslinks were generated using Circos[24]. Venn diagrams were generated using eulerAPE[25].

Supplementary References

1. Kurze, A. et al. A positively charged channel within the Smc1/Smc3 hinge required for sister chromatid cohesion. *EMBO J* **30**, 364-78 (2011).
2. Rinner, O. et al. Identification of cross-linked peptides from large sequence databases. *Nat Methods* **5**, 315-8 (2008).
3. Herzog, F. et al. Structural probing of a protein phosphatase 2A network by chemical cross-linking and mass spectrometry. *Science* **337**, 1348-52 (2012).
4. Altschul, S.F. et al. Gapped BLAST and PSI-BLAST: a new generation of protein database search programs. *Nucleic Acids Res* **25**, 3389-402 (1997).
5. Katoh, K. & Toh, H. Recent developments in the MAFFT multiple sequence alignment program. *Brief Bioinform* **9**, 286-98 (2008).
6. Waterhouse, A.M., Procter, J.B., Martin, D.M., Clamp, M. & Barton, G.J. Jalview Version 2--a multiple sequence alignment editor and analysis workbench. *Bioinformatics* **25**, 1189-91 (2009).
7. Delorenzi, M. & Speed, T. An HMM model for coiled-coil domains and a comparison with PSSM-based predictions. *Bioinformatics* **18**, 617-25 (2002).
8. Lupas, A., Van Dyke, M. & Stock, J. Predicting coiled coils from protein sequences. *Science* **252**, 1162-4 (1991).
9. McDonnell, A.V., Jiang, T., Keating, A.E. & Berger, B. Paircoil2: improved prediction of coiled coils from sequence. *Bioinformatics* **22**, 356-8 (2006).
10. Gligoris, T.G. et al. Closing the cohesin ring: structure and function of its Smc3-kleisin interface. *Science* **346**, 963-7 (2014).
11. Haering, C.H. et al. Structure and stability of cohesin's Smc1-kleisin interaction. *Mol Cell* **15**, 951-64 (2004).
12. Hornbeck, P.V. et al. PhosphoSitePlus, 2014: mutations, PTMs and recalibrations. *Nucleic Acids Res* **43**, D512-20 (2015).
13. Ansari, M. et al. Genetic heterogeneity in Cornelia de Lange syndrome (CdLS) and CdLS-like phenotypes with observed and predicted levels of mosaicism. *J Med Genet* **51**, 659-68 (2014).
14. Boyle, M.I., Jespersgaard, C., Brondum-Nielsen, K., Bisgaard, A.M. & Tumer, Z. Cornelia de Lange syndrome. *Clin Genet* **88**, 1-12 (2015).

15. Gervasini, C. et al. Cornelia de Lange individuals with new and recurrent SMC1A mutations enhance delineation of mutation repertoire and phenotypic spectrum. *Am J Med Genet A* **161A**, 2909-19 (2013).
16. Gil-Rodriguez, M.C. et al. De novo heterozygous mutations in SMC3 cause a range of Cornelia de Lange syndrome-overlapping phenotypes. *Hum Mutat* **36**, 454-62 (2015).
17. Mannini, L., Cucco, F., Quarantotti, V., Krantz, I.D. & Musio, A. Mutation spectrum and genotype-phenotype correlation in Cornelia de Lange syndrome. *Hum Mutat* **34**, 1589-96 (2013).
18. Campbell, R.E. et al. A monomeric red fluorescent protein. *Proc Natl Acad Sci U S A* **99**, 7877-82 (2002).
19. Chari, A. et al. ProteoPlex: stability optimization of macromolecular complexes by sparse-matrix screening of chemical space. *Nat Methods* (2015).
20. Niesen, F.H., Berglund, H. & Vedadi, M. The use of differential scanning fluorimetry to detect ligand interactions that promote protein stability. *Nat Protoc* **2**, 2212-21 (2007).
21. Ladurner, R. et al. Cohesin's ATPase Activity Couples Cohesin Loading onto DNA with Smc3 Acetylation. *Curr Biol* **24**, 2228-37 (2014).
22. Leitner, A. et al. Expanding the chemical cross-linking toolbox by the use of multiple proteases and enrichment by size exclusion chromatography. *Mol Cell Proteomics* **11**, M111 014126 (2012).
23. Walzthoeni, T. et al. False discovery rate estimation for cross-linked peptides identified by mass spectrometry. *Nat Methods* **9**, 901-3 (2012).
24. Krzywinski, M. et al. Circos: an information aesthetic for comparative genomics. *Genome Res* **19**, 1639-45 (2009).
25. Micallef, L. & Rodgers, P. eulerAPE: drawing area-proportional 3-Venn diagrams using ellipses. *PLoS One* **9**, e101717 (2014).
